# Bioprospecting of desert actinobacteria with special emphases on griseoviridin, mitomycin C and a new bacterial metabolite producing *Streptomyces* sp. PU-KB10–4

**DOI:** 10.1186/s12866-023-02770-8

**Published:** 2023-03-15

**Authors:** Maira Saleem, Ashba Hassan, Feina Li, Qinpei Lu, Larissa V. Ponomareva, Sean Parkin, Chenghang Sun, Jon S. Thorson, Khaled A. Shaaban, Imran Sajid

**Affiliations:** 1 Institute of Microbiology and Molecular Genetics (MMG), University of the Punjab, Lahore, 54590 Pakistan; 2 Center for Pharmaceutical Research and Innovation (CPRI), College of Pharmacy, University of Kentucky, Lexington, Kentucky 40536 USA; 3 Department of Pharmaceutical Sciences, College of Pharmacy, University of Kentucky, Lexington, Kentucky 40536 USA; 4 Institute of Medicinal Biotechnology (IMB), Chinese Academy of Medical Sciences & Peking Union Medical College, Beijing, China; 5 Department of Chemistry, University of Kentucky, Lexington, Kentucky 40506 USA

**Keywords:** Kubuqi desert, Actinobacteria, Natural products, Antibacterial, Anticancer, *Streptomyces*, Mitomycin, 4-hydroxycinnamide

## Abstract

**Background:**

Bioprospecting of actinobacteria isolated from Kubuqi desert, China for antibacterial, antifungal and cytotoxic metabolites production and their structure elucidation.

**Results:**

A total of 100 actinobacteria strains were selectively isolated from Kubuqi desert, Inner Mongolia, China. The taxonomic characterization revealed *Streptomyces* as the predominant genus comprising 37 different species, along with the rare actinobacterial genus *Lentzea*. The methanolic extracts of 60.8% of strains exhibited potent antimicrobial activities against *Staphylococcus aureus*, *Micrococcus luteus*, *Bacillus subtilis*, *Escherichia coli*, *Salmonella enterica*, *Saccharomyces cerevisiae* and high to mild in vitro cytotoxicity against PC3 (prostate cancer) and A549 (lung carcinoma) cell lines. The metabolomics analysis by TLC, HPLC-UV/vis, HPLC-MS and NMR showed the presence of compounds with molecular weights ranging from 100 to 1000 Da. The scale-up fermentation of the prioritized anti-Gram-negative strain PU-KB10–4 (*Streptomyces griseoviridis*), yielded three pure compounds including; griseoviridin (**1**; 42.0 mgL^− 1^) with 20 fold increased production as compared to previous reports and its crystal structure as monohydrate form is herein reported for the first time, mitomycin C (**2**; 0.3 mgL^− 1^) and a new bacterial metabolite 4-hydroxycinnamide (**3**; 0.59 mgL^− 1^).

**Conclusions:**

This is the first report of the bioprospecting and exploration of actinobacteria from Kubuqi desert and the metabolite 4-hydroxycinnamide (**3**) is first time isolated from a bacterial source. This study demonstrated that actinobacteria from Kubuqi desert are a potential source of novel bioactive natural products. Underexplored harsh environments like the Kubuqi desert may harbor a wider diversity of actinobacteria, particularly *Streptomyces*, which produce unique metabolites and are an intriguing source to develop medicinally valuable natural products.

**Supplementary Information:**

The online version contains supplementary material available at 10.1186/s12866-023-02770-8.

## Introduction

As the twenty-first century unfolds, the threat of antimicrobial resistance (AMR) to human health gets more pervasive. In 2019, an estimated 4.95 million deaths were attributed to bacterial AMR [1]. Antibiotic-resistant illnesses cause around 2.8 million hospitalizations and approximately 35,000 fatalities each year in the United States [2]. As a result, ensuring the efficacy of antimicrobials is essential for human health in the years to come [3]. Antibiotic research in the pharmaceutical business has taken many forms over the last two decades. Random screening for new active compounds followed by chemical optimization employing simple antibiotic activity as primary selection has been the winning approach [4]. Novel culture techniques boosted throughput with minimum resources, solving one of the conventional fermentation’s primary limits. Similarly, advanced bacterial isolation processes have also helped discover new species of actinomycetes [5].

*Streptomyces* is the largest bacterial genus in Actinomycetota, a dominating bacterial phylum [6]. This is the prominent source of natural compounds and their synthetic analogues make up a considerable fraction of clinically useful antibiotics. Over 70% of actinobacterial metabolites show antibacterial activity [7], and 64% of the known antibiotic families are produced by filamentous actinomycetes [8], making this an extremely important source of natural products. Actinobacteria are a group of filamentous, Gram-positive bacteria known as efficient producers of secondary metabolites with a wide range of biological activities including antibacterial [9, 10], anti-biofilm [11, 12], antioxidant [13], antitumor [14], antifungal [15], and anti-inflammatory [16] activities. The significant contribution of actinobacteria in drug discovery has encouraged scientists to explore these bacteria from diverse and untapped environments [17]. Many unique structures of natural products have been isolated from actinobacteria, particularly from the underexplored extreme environments [18].

**Fig. 1:**
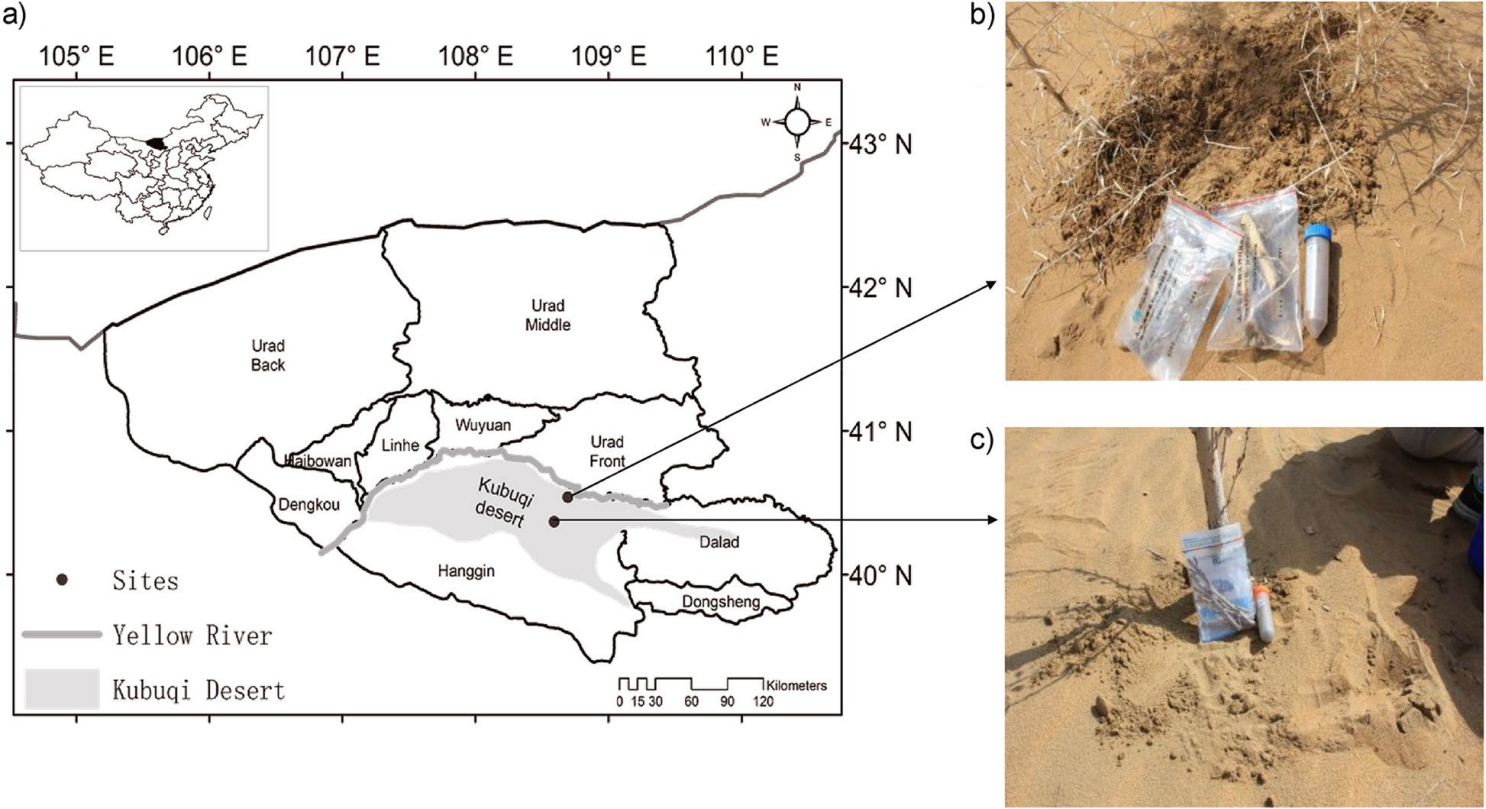
**A** Map of Kubuqi desert, Inner Mongolia, China (Map used with permission) [32]. **b** and **c** Samples collected from different areas of Kubuqi desert, China

Underexplored sites, such as desert environments, are believed to be abundant with novel actinobacteria due to unusual climatic conditions [19, 20]. Such arid and hyper-arid deserts cover almost one-quarter of the Earth’s land surface. Actinobacteria have been isolated from desert soils of Amargosa desert in Nevada [21], the Mojave [22], Mongolian [23], Sahara [24], Tataouine [25], Atacama Desert [26], Cholistan desert [27–29], and the Taklamakan desert in Xinjiang province, China [30]. In China, nearly 27% of the land area is desert [31]. The Ordos desert is located in the south of Inner Mongolia in the People’s Republic of China. It consists of two big deserts: Kubuqi desert and Muu-us desert (Fig. 1). The Kubuqi Desert is the seventh-largest in China, covering 18,600 km^2^ [33]. The diversity of actinobacteria from these environments and their metabolic capabilities are unexplored.

Herein we describe the taxonomy and metabolomics profiling of actinobacteria strains isolated from Kubuqi desert, China. Taxonomy was based on 16S rRNA sequencing, bioactivity analysis included in vitro antibacterial, antifungal and anticancer assays and metabolite screening employed standard chromatographic techniques (TLC, HPLC-UV/vis, and HPLC-MS). The scale-up fermentation of one representative strain named PU-KB10–4, selected based on metabolomics profile and bioactivity, is also reported. The isolation and structure elucidation revealed this strain to be the producer of a new bacterial metabolite.

## Results

### Taxonomy of the selected actinobacteria strains

A total of 100 strains were recovered from the Kubuqi desert soil samples. Most of the strains produced rounded, raised, embedded, and hard textured colonies with colony size ranging from 2 to 5 mm and a few produced white powder like spore mass on the agar plates (Additional file 1: Fig. S1). Some strains produced light brown (PU-KB1–4), dark brown (PU-KB10–4, PU-KB10–11, PU-KB12–15), and black (PU-KB2–2) diffused pigments. The substrate and aerial mycelia of certain strains also displayed various colors e.g., PU-KB10–4 displayed light yellow colored substrate mycelia and dark yellow colored aerial mycelia.

Most of the strains were melanin producers. The strains were able to use different sugars e.g. D-xylose (63.5%), Mannitol (84.7%), Mannose (96.4%), Sucrose (63.5%), Fructose (77.6%), and Arabinose (63.5%) as the sole source of carbon. Glucose (98.8%) was utilized by maximum number of the strains. The strains PU-KB3–3, PU-KB3–6, PU-KB5–1, PU-KB5–2, PU-KB5–7, PU-KB5–25, PU-KB10–1, PU-KB10–4, and PU-KB10–11 utilized all sugars that were tested as sole source of carbon. The esculin and urea were hydrolyzed by 77.6% and 96.4% strains respectively. Most of the strains utilized organic acids such as trisodium citrate (90.5%) and sodium malonate (85.8%). Likewise, most of the strains exhibited organic acid formation (92.9%) including PU-KB9–8, PU-KB9–11, and PU-KB12–8 being the prominent ones. Most of the strains were also positive for oxalate utilization (89.4%) and exhibited clear zone on the agar supplemented with oxalate (Additional file 2: Table S1).

**Fig. 2:**
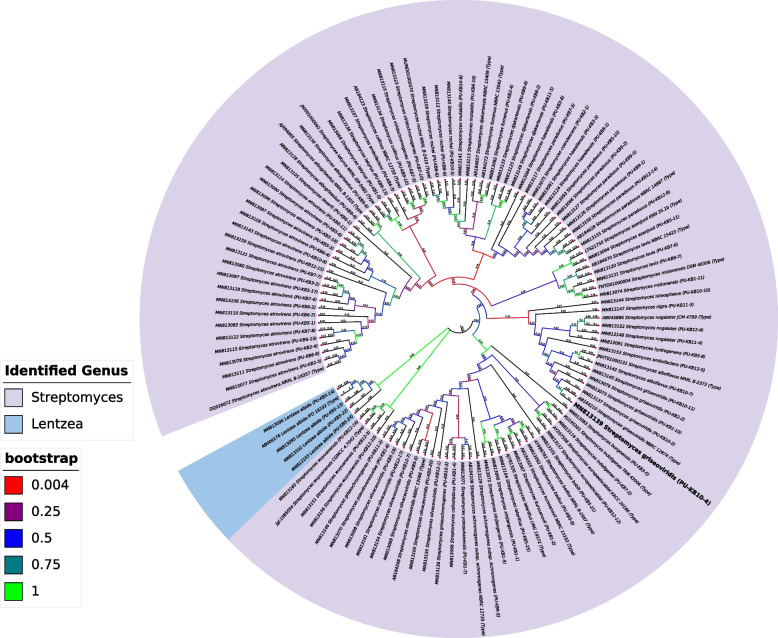
The Neighbor-Joining phylogenetic tree of Kubuqi desert actinobacteria [34]. The percentage of replicates in which the associated taxa clustered together in the bootstrap test (1000 replicates) are shown next to the branches [35]. The tree is drawn to scale, with branch lengths in the same units as those of the evolutionary distances used to infer the phylogenetic tree. The evolutionary distances were computed using the Maximum Composite Likelihood method [36] and are in the units of the number of base substitutions per site. This analysis involved 94 nucleotide sequences. All ambiguous positions were removed for each sequence pair (pairwise deletion option). There were a total of 1416 positions in the final dataset. Evolutionary analyses were conducted in MEGA11 [37].

The Neighbor-Joining phylogenetic tree of Kubuqi desert actinobacteria was constructed (Fig. 2). The 16S rRNA gene sequencing data of 96 strains showed percentage similarity with actinobacteria, the term which has recently been updated to phylum Actinomycetota [38]. The similarity index was obtained from BLAST [39] analysis on EzBioCloud [40]. Among all the strains, 27 strains displayed 100% similarity with different species of *Streptomyces*, while 68 strains exhibited > 99% but < 100% similarity with the genus *Streptomyces*. Only one strain PU-KB10–7 displayed < 99% similarity; it was 98.97% similar to the type strain *Streptomyces rochei* (SI, Table S2). The strain PU-KB6–1 exhibited 100% similarity with *Streptomyces deserti*, similarly the strains PU-KB7–6 and PU-KB9–7 displayed 100% similarity with *Streptomyces levis* (Additional file 3: Table S2). The strains PU-KB1–5 and PU-KB9–13 exhibited 100% similarity with *Streptomyces iakyrus*. Strain PU-KB5–9 exhibited 99.47% similarity with *Streptomyces badius*. The strains PU-KB6–6 and PU-KB6–9 displayed 100% while PU-KB10–5 exhibited 99.89% similarity with *Streptomyces rochei*. Four strains including PU-KB5–13, PU-KB5–14, PU-KB5–22, and PU-KB5–24 were closely related to the rare actinobacteria genus *Lentzea* and exhibited close similarity to *Lentzea albida* with 99.19%, 99.24%, 99.25% and 99.29%, respectively.

### Antimicrobial activity

**Fig. 3:**
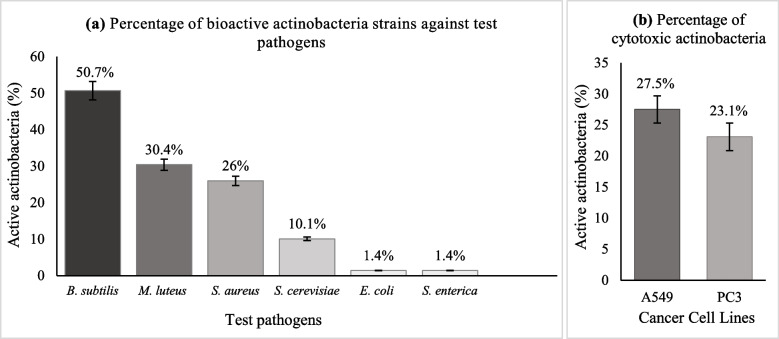
**a** Percentage of actinobacteria strains which showed activity against Gram-positive, Gram-negative bacteria and yeast **b** Percentage of actinobacteria strains which showed cytotoxicity against cancer cell lines

The average OD_600_ values after normalizing with controls showed significant antimicrobial actinobacteria strains. Among all the strains, 35 showed inhibitory activity against *B. subtilis*. The most active strains were PU-KB2–3 and PU-KB12–7 with 94.2% and 94.3% inhibition respectively. While, 21 strains showed activity against *M. luteus* with 95–98% inhibition. The 18 actinobacterial strains inhibited *S. aureus*. Interestingly, only one strain PU-KB10–4 showed significant anti-Gram-negative activity against *E. coli* and antifungal activity against *S. cerevisiae* along with other five strains including PU-KB2–4, PU-KB3–2, PU-KB3–3, PU-KB10–7, and PU-KB11–4. The percentage of actinobacteria strains active against test pathogens is given in Fig. 3a. The percentage inhibition of each strain is given in Additional file 4: Table S3.

### In-vitro cytotoxicity assay

The methanolic crude extracts of actinobacterial strains were tested for cytotoxicity by resazurin florescent assay. The strains PU-KB5–19, PU-KB9–5, PU-KB10–9, and PU-KB12–12 exhibited 0–2% cell viability (98–100% cytotoxicity) against both PC3 and A549 cell lines which indicated that these strains are highly cytotoxic. The extracts of 11 strains showed up to 15–20% cell viability (80–85% cytotoxicity) in both cell lines. Some strains exhibited interesting results, like methanolic extract of PU-KB2–2 exhibited approximately 50–55% cell viability (45–50% cytotoxicity) in A549 but did not show any significant cell viability in a PC3 cell line. The extract of strain PU-KB5–11 resulted in 60% of viable cells (40% cytotoxicity) in the PC3 cell line. The extract of strain PU-KB6–10 showed almost 55% cell viability (45% cytotoxicity) in PC3 but 80% cell viability (20% cytotoxicity) in the A549 cell line, which means it was more cytotoxic against PC3 cell lines. The percentage of mild to high cytotoxic actinobacteria against cancer cell line is given in Fig. 3b. The cytotoxicity percentage of each strain against both cell lines is given in Additional file 4: Table S3.

### Chemical profiling

The thin-layer chromatography (TLC) analysis was done by visualizing the TLC plates under UV (254 nm and 366 nm) and staining with anisaldehyde/H_2_SO_4_ reagent. The methanolic extracts of the strains PU-KB1–6, PU-KB5–11, PU-KB6–10, PU-KB6–13, PU-KB8–2, PU-KB10–4, and PU-KB12–3 exhibited prominent bands under UV. The strains PU-KB5–11, PU-KB5–15, PU-KB6–10, and PU-KB9–10. PU-KB2–3, PU-KB2–4, PU-Kb3–2, PU-KB5–17, PU-KB6–7, PU-KB7–4, PU-KB7–7, and PU-KB7–8 showed yellow-orange-colored bands on TLC, indicating the presence of some actinomycin-like compounds (Additional file 5: Fig. S2).

The HPLC-UV/vis analysis exhibited very diverse and exciting peaks at different retention times with characteristic UV signature of various bioactive compounds. The strain with selective activity against *Bacillus subtilis*, PU-KB5–11 showed three significant peaks at *t*_R_ 10.344 min, *t*_R_ 17.903 min, and *t*_R_ 19.163 min (Additional file 6: Fig. S3). The crude extract of the anti-Gram-negative strain PU-KB10–4 produced one prominent peak at *t*_R_ 13.018 min and two minor peaks at *t*_R_ 9.968 min and *t*_R_ 22.782, respectively (Additional file 7: Fig. S4). The crude extracts of other active strains like PU-KB5–19, PU-KB6–10, PU-KB8–2, PU-KB9–11, PU-KB12–3, and PU-KB12–13 showed the presence of more than 3–4 significant compounds in the extracts at different retention times (Table 1).

**Table 1:** Metabolomics profile of the bioactive actinobacterial strains originated from Kubuqi desert China

Strains	Thin-layer chromatography (TLC) profile		HPLC- retention time (*t*_R_/min)	(+) and (−)-ESI-MS: (m/z)	Molecular Weight	Antimicrobial, antifungal, and cytotoxic activity
	UV visualization	Anisaldehyde/H_**2**_SO_**4**_ Staining				
PU-KB2–3	254 nm 3 bands 366 nm 3 bands	3 orange bands	33.201	1255[M + H]^+^, 1253[M-H]^−^	1254	Approx. 65% cytotoxic against A549 and PC3 cell lines
PU-KB2–4	254 nm 3 bands 366 nm 3 bands	3 orange bands	33.201	1255[M + H]^+^, 1253[M-H]^−^	1254	Approx. 65% cytotoxic against A549 and PC3 cell lines
PU-KB3–2	254 nm 2 bands 366 nm 2 bands	2 orange bands	33.201	1255[M + H]^+^, 1253[M-H]^−^	1254	Approx. 65% cytotoxic against A549 and PC3 cell lines
PU-KB5–11	254 nm 4 bands 366 nm 4 bands	1 dark blue band, 3 light blue bands	13.953 22.222 23.676	587[M + H]^+^, 585 [M-H]^−^ 743[M + H]^+^, 777 [M + Cl]^−^ 743[M + H]^+^, 777 [M + Cl]^−^	586 742 742	Highly active only against *B. subtilis*. Approx. 40% cytotoxic against PC3 cell line
PU-KB5–17	254 nm 1 band 366 nm 4 bands	2 orange bands	33.201	1255[M + H]^+^, 1253[M-H]^−^	1254	Approx. 65% cytotoxic against A549 and PC3 cell lines
PU-KB5–19	254 nm 1 band 366 nm 3 bands	3 light purple bands	24.393	423[M + H]^+^, 421 [M-Cl]^−^	422	Highly active only against *B. subtilis*
PU-KB6–2	254 nm 3 bands 366 nm 3 bands	3 orange bands	33.201	1255[M + H]^+^, 1253[M-H]^−^	1254	Approx. 65% cytotoxic against A549 and PC3 cell lines
PU-KB6–7	254 nm 1 band 366 nm 1 band	1 orange band	33.201	1255[M + H]^+^, 1253[M-H]^−^	1254	Approx. 65% cytotoxic against A549 and PC3 cell lines
PU-KB6–10	254 nm 4 bands 366 nm 3 bands	1 yellow band, 1 blue band, 1 purple band	11.904	518[M + H]^+^, 516 [M-H]^−^	517	Highly active against only *M. luteus*. Approx. 40% cytotoxic against A549 and 65% against PC3 cell line
PU-KB7–4	254 nm 3 bands 366 nm 6 bands	3 orange bands	33.201	1255[M + H]^+^, 1253[M-H]^−^	1254	Approx. 65% cytotoxic against A549 and PC3 cell lines
PU-KB7–7	254 nm 3 bands 366 nm 5 bands	3 orange bands, 1 yellow band	33.201	1255[M + H]^+^, 1253[M-H]^−^	1254	Approx. 70% cytotoxic against A549 and PC3 cell lines
PU-KB7–8	254 nm 6 bands 366 nm 5 bands	4 orange bands	33.201	1255[M + H]^+^, 1253[M-H]^−^	1254	Approx. 70% cytotoxic against A549 and PC3 cell lines
PU-KB8–2	254 nm 3 bands 366 nm 3 bands	2 yellow bands, 1blue band	21.387 24.852	1137[M + H]^+^, 1135[M-H]^−^ 1119[M + H]^+^, 1117[M-H]^−^	1136 1118	Potent activity against *B. subtilis, M. luteus* and *S. aureus.* Approx. 70% cytotoxic against A549 and PC3 cell lines
PU-KB9–11	254 nm 1 band 366 nm 1 band	4 pale yellow bands	15.354	299[M + H]^+^, 343[M + HCOO]^−^	298	Highly active only against *B. subtilis* and *M. luteus*. Approx. 20% cytotoxic against PC3 cell line
PU-KB10–4	254 nm 2 bands 366 nm 4 bands	0 bands	16.934	460[M + H]^+^, 476[M-OH]^−^	459	Potent activity against *B. subtilis, M. luteus* and *S. aureus.* Highly active against Gram-negative *E. coli*. Approx. 40% cytotoxic against PC3 cell line
PU-KB11–5	254 nm 3 bands 366 nm 3 bands	2 pale yellow bands, 1 light purple band	15.423 21.385	292[M + H]^+^, 290[M-H]^−^ 1137[M + H]^+^, 1135[M-H]^−^	291 1136	No preliminary antimicrobial activity. Approx. 30% cytotoxic against PC3 cell line
PU-KB12–3	254 nm 1 band 366 nm 0 band	0 band	24.078 22.982	373[(M-2H_2_O) + H]^+^, 407[M-H]^−^ 466[M + H]^+^, 464[2 M-H]^−^	408 465	Potent activity against *Bacillus subtilis, M. luteus* and *S. aureus*
PU-KB12–13	254 nm 1 band 366 nm 0 band	0 band	19.617	304[M + H]^+^, 302[M-H]^−^	303	Approx. 40% inhibition of *S. cerevisiae*

The HPLC-MS analysis showed the presence of different compounds with various molecular masses such as the strain PU-KB5–11 showed three major peaks, at *t*_R_ 13.953 min with 586 Da, interestingly the peaks at *t*_R_ 22.222 min and 23.676 min had the same mass of 742 Da. The strain PU-KB8–2 had two significant peaks and the calculated mass for both was 1136 Da and 1118 Da, respectively (Additional file 8: Fig. S5). The strain PU-KB9–11 had one prominent peak of molecular mass 298 Da (Additional file 9: Fig. S6). The only anti-Gram-negative strain PU-KB10–4 showed various peaks, but the most significant one had a molecular mass of 459 Da (Additional file 10: Fig. S7). The chromatogram of strain PU-KB11–5 showed two significant peaks with the molecular mass of 291 Da and 1136 Da, respectively. Similarly, the strain PU-KB12–3 also showed two significant peaks with mass 465 Da and 408 Da, respectively (Additional file 11: Fig. S8). Nine strains PU-KB2–3, PU-KB2–4, PU-KB3–2, PU-KB5–17, PU-KB6–2, PU-KB6–7, PU-KB7–4, PU-KB7–7, and PU-KB7–8 all were cytotoxic and showed many different peaks, but one peak was identical in all at *t*_R_ 33.201 min, with the molecular weight of 1254 Da (Additional files 12, 13, 14 and 15: Fig. S9-S12) consistent with actinomycin D.

### Structure elucidation, X-ray crystallography and bioactivity of metabolites from *Streptomyces* sp. PU-KB10–4

**Fig. 4:**
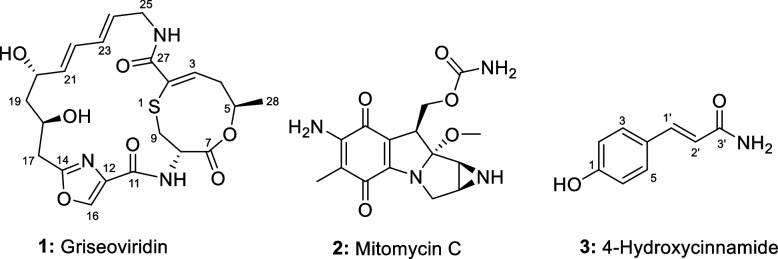
Chemical structures of compounds **1–3**

The chemical structures of the known compounds **1–3** (Fig. 4) were established based on 1D and 2D NMR spectroscopy, mass spectrometry, HPLC-UV/vis, and HPLC-MS data and by comparing with literature data. Compounds **1–3** were identified and established as griseoviridin (**1**; also known as F-1370-B), mitomycin C (**2**) and 4-hydroxycinnamide (**3**).

**Fig. 5:**
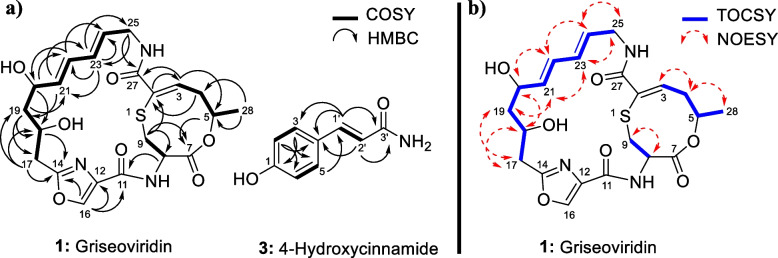
**a** ^1^H,^1^H-COSY (▬) and selected HMBC (→) correlations of compounds **1** and **3**. **b** TOCSY (  ) and selected NOESY (  ) correlations of compound **1**

These compounds belong to different classes of natural products, which include the modified cyclic peptide/23-membered macrolactone antibiotic **(1)** (Additional files 16, 17, 18, 19, 20, 21, 22, 23, 24 and 25, Fig. S13-S22), a quinone/aziridine **(2)** (Additional files 26, 27, 28, 29 and 30, Fig. S23-S27) and phenylpropanoid **(3)** (Additional files 27, 28, 29, 30, 31, 32, 33, 34, 35, 36, 37, 38, 39 and 40, Fig. S28-S37) (Fig. 5). The physicochemical properties of compounds **1–3** are given in Additional file 41.

**Fig. 6:**
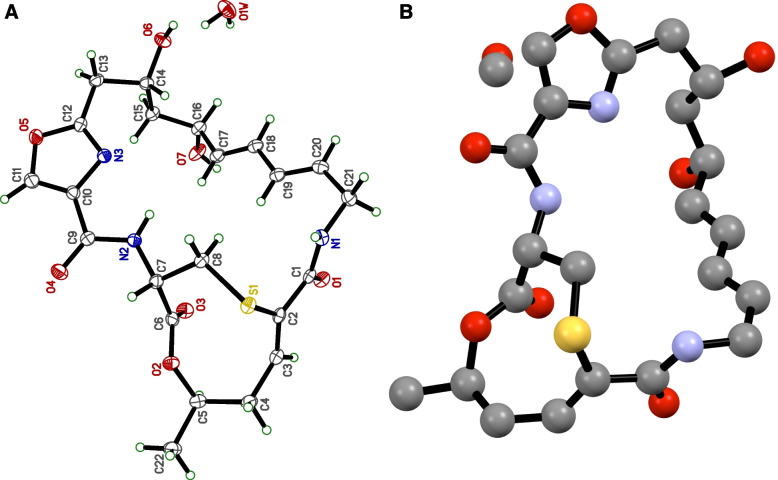
**a** Crystal structure of griseoviridin **(1)** in monohydrate form; **b** The reported crystal structure of griseoviridin **(1)** as a methanol solvate [55]

Mitomycin C **(2)** belongs to the quinone/aziridine-mitomycin structural class, and this class of compounds played an essential role as a chemotherapeutic drug and have been used frequently in the treatment of various types of cancers [41–52]. It is pertinent to mention here that, while the molecular structure of griseoviridin **(1)** has been published previously as a methanol solvate [53–55] we report herein for the first time the crystal structure of **1** as a monohydrate form (Fig. 6). It is also important to note that this is the first report of 4-hydroxycinnamide **(3)** production from bacteria. Crystallographic data for the structure of compound **1** have been submitted to the Cambridge Crystallographic Data Centre (CCDC) as supplementary publication 2,055,325.

**Fig. 7:**
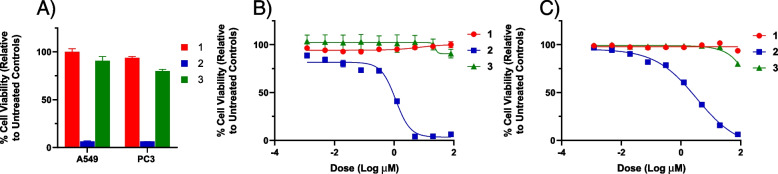
**a** % Viability of A549 (non-small lung) and PC3 (prostate) human cancer cell lines (after 72 h) at 80 μM concentration of compounds **1**–**3**. **b** Dose-response of compounds **1**–**3** against A549 (non-small cell lung) human cancer cell line (72 h). (c) Dose-response of compounds **1**–**3** against PC3 (prostate) human cancer cell line (72 h). A549: IC_50_ for the compounds 1 ((> 80 μM), 2 (1.06 ± 0.04 μM) and 3 (> 80 μM). PC3: IC_50_ for the compounds 1 (> 80 μM), 2 (3.04 ± 0.25 μM) and 3 (> 80 μM)

All the isolated pure compounds from *Streptomyces* sp. PU-KB10–4 (**1–3**) were tested for standard antimicrobial, antifungal, and cytotoxicity against cancer cell lines. The IC50 value was calculated against the cancerous cell lines; A549 and PC3. Among them, only compound **2** exhibited pronounced activity at the concentrations ≤80 μ mol in the cytotoxicity assays (A549, IC_50_ 1.06 μM; PC3, IC_50_ 3.04 μM) (Fig. 7).

## Discussion

To the best of our knowledge, there is no documented study about the actinobacterial diversity and isolation from the Kubuqi desert, Inner Mongolia, China. In this study, 100 actinobacteria strains were isolated, identified, and profiled for bioactivity and metabolomics. It was determined that majority of the strains belonged to the Streptomycetacae family, specifically distinct species of the genus *Streptomyces*, and that a few strains belonged to the rare actinobacterial genus *Lentzea*, from Pseudonocardiaceae family. Isolating rare actinobacterial genera is significant since they are slow growers and difficult to retrieve from mixed cultures, hence less often explored for bioactivity screening. *Lentzea* species have produced six novel diene glycosides with anti-HIV integrase activity [56, 57]*.* The high frequency of *Streptomyces* genus isolation from desert soils has also been reported in earlier investigations [27, 58]. Although the *Streptomyces* genus has an enormous variety of species, as 38 different *Streptomyces* species have been identified from this location, only a few have been studied extensively for bioactivity screening and metabolomics analysis.

The argument that exploring unique and untapped ecological niche might yield better diversity of actinobacteria and unique metabolites is supported by the outcome of this study. As the results showed, about 60.8% of the strains exhibited activity against pathogenic test organisms. Among the active strains, 83.3% were anti-Gram-positive, i.e. 50.7% of the strains exhibited activity against *B. subtilis,* 30.4% were active against *M. luteus* and 26.0% exhibited activity against *S. aureus*. This threshold of bioactivity is significant as compared to previous studies where desert actinobacteria were screened and about 43.7% of the strains were active against Gram-positive pathogens [59]. In case of activity against Gram-negative bacteria, the strain PU-KB10–4 was found to be the only strain exhibiting prominent growth inhibition of *E. coli* and *S. enterica*. In a previous study [60], five out of 134 isolates from desert soil showed anti-Gram-negative activity, this is in accordance with the routine antibiotics screening outcomes that the ratio of obtaining anti-Gram-negative strains is usually low as compared to the anti-Gram positive strains. Similarly, seven strains exhibited significant activity against yeast (*S. cerevisiae*), this activity might be due to the non-selective cytotoxicity of the strains but interestingly four of these antifungal strains including PU-KB3–3, PU-KB10–7, PU-KB10–8 and PU-KB11–4 exhibited low cytotoxicity against human cell lines, which indicated that these strains could be promising candidates for antifungal compounds screening. In the in-vitro cytotoxicity, determined against two human cancer cell lines, the extracts of some strains were found to be highly cytotoxic, leaving only 0–2% viable cells. Among all the strains tested for in-vitro cytotoxicity, about 16% strains showed mild cytotoxicity, up to 20–30% cell viability. The high to mild or low cytotoxicity of these desert actinobacteria along with a kind of broad spectrum antimicrobial activity predict that devilling conditions in the desert might have enabled these bacteria to develop unique or unusual biosynthetic pathways to produce diverse secondary metabolites.

The combined bioactivity data and metabolomic profiles (TLC, HPLC-UV/vis, and HPLC-MS) of the strains helped to predict the ability of the strains to produce unique or common compounds, which in turn helped to exercise dereplication and to prioritize the strains for preparative screening. The seven strains including PU-KB5–11, PU-KB6–10, PU-KB8–2, PU-KB9–9, PU-KB10–4, PU-KB12–3 and PU-KB12–13 were designated as priority strains for future studies by scale up studies. Among these, the strain PU-KB5–11, designated as *Streptomyces asenjonii*, exhibited antibacterial activity against *B. subtilis* and cytotoxic activity against PC3, while in a previous study [61] *Streptomyces asenjonii* exhibited mild anti-Gram-negative activity as well. The strain PU-KB8–2 and PU-KB9–9, identified as *Streptomyces djakartensis* exhibited cytotoxicity and growth inhibition of *B. subtilis, M. luteus* and *S. aureus* which is in accordance with the already reported study [62], in which they isolated two antibacterial compounds ((*E*)-2-methoxy-1,4 naphthoquinone-1-oxime and *N*-acetyltryptamine) from the fermented broth of *Streptomyces djakartensis* NW35. The strain PU-KB12–3, identified as *Streptomyces wuyuanensis* exhibited only anti-Gram-positive activity against *B. subtilis, M. luteus* and *S. aureus*, as previously reported [63] *Streptomyces wuyuanensis* inhibited only Gram-positive bacteria.

The nine strains in the collection including PU-KB2–3, PU-KB2–4, PU-KB3–2, PU-KB5–17, PU-KB6–2, PU-KB6–7, PU-KB7–4, PU-KB7–7 and PU-KB7–8 identified as *Streptomyces atrovirens* showed high cytotoxicity against PC3 and A549 cell lines. The methanolic extracts of these strains exhibited characteristics yellowish spot on TLC and the HPLC-MS spectra showed the presence of an ion peak with molecular mass of *m/z* 1254, hence it was concluded that all these strains are actinomycin D producing strains. The dark brown culture of strain PU-KB6–7 was due to high concentration of metal-complex siderophores (Fe – complex), which was consistent with the detected peak in HPLC/UV analysis at 18.8 min, and the low concentration of actinomycin D at 25.3 min. However, the studies have reported [64] that *Streptomyces atrovirens* produce the compound 4-hydroxy-3-(3-methylbut-2-enyl) benzaldehyde (C_12_H_14_O_2_; MW = 190) which exhibited broad spectrum antimicrobial activity and low cytotoxicity against human epithelial HL cell lines.

The preparative screening of one priority strain (PU-KB10–4), which showed anti-Gram-negative activity, yielded three pure compounds, including griseoviridin **(1)**, mitomycin C **(2)**, and 4-hydroxycinnamide **(3)**. Compound **1** was the major compound of the strain extract > 42 mgL^˗1^, and compound **3** was identified as a new bacterial metabolite. Griseoviridin **(1)**, originally isolated and reported from *Streptomyces griseus* in 1954, and was the first representative of the streptogramins (group A), [65]. The streptogramin antibiotics are a family of natural products that have been isolated from soil *Streptomyces* [66]. The Previously reported production levels of griseoviridin **(1)** were in the range of 1.3–2.7 mgL^˗1^ [67, 68], while PU-KB10–4 under our growth conditions afforded > 20-fold improvement in **1** production. There are reports, which showed the production of griseoviridin from *Streptomyces griseoviridis* [69], so the high production of griseoviridin by this strain could be due to different media components and the specific growth conditions employed in its fermentation. In addition, mitomycin C **(2)** was also identified as the PU-KB10–4 metabolite responsible for the anti-Gram-negative activity, in accordance with the previously reported mitomycin C activity [70–72]. Mitomycin C **(2)** is an aziridine-containing natural product reported previously from several bacterial strains, including *Streptomyces caespitosus*, *Streptomyces ardus*, *Streptomyces verticillatus*, *Streptomyces fervens-phenomyceticus*, and *Micromonospora* sp. So, this seems to be the first report of production of mitomycin C **(2)** from the *Streptomyces griseoviridis*. Mitomycin C has been reported with various types of bioactivities, including anticancer, antibacterial and antifungal [41, 43, 51, 70–72]. Compound **3** has also been isolated and identified as the PU-KB10–4 metabolite and is reported herein for the first time as new bacterial metabolite. 4-Hydroxycinnamamide has been reported previously as plant metabolite from the leaves of *Hosta longipes* [73] and *Tetrastigma hemsleyanum* [74], and by synthesis [75]. The free acid forms of compound **3** (cinnamic and 4-hydroxycinnamic acids) have also been reported from engineered microbes [76].

We report herein the isolation, diversity and metabolomics profiling of actinobacteria from Kubuqi desert, China, as a new and previously untapped site for the isolation of actinobacteria. The genus *Streptomyces* was found to be the most frequently harboring actinobacterial genus for which 38 different species were identified. Based on the bioactivity screening and metabolomics profiling, 9 strains were selected as priority strains which are potential candidates for future scale up studies for compound purification and identification. The scale-up fermentation of one of the priority strains PU-KB10–4 yielded three bioactive compounds including; griseoviridin, mitomycin C and the new bacterial metabolite 4-hydroxycinnamid. Overall the study shows, that Kubuqi desert China harbors a remarkable diversity of actinobacteria, especially of *Streptomyces*, which could be the efficient producers of unique metabolites and are an attractive source for natural products discovery.

## Methods

### Sample collection and selective isolation of actinobacteria

The soil and sand samples were collected from different locations in the Kubuqi desert, Inner Mongolia, China (Longitude 109°19′46.1“E and Latitude 40°19’32.6”N). The samples were collected from the rhizosphere of various plants and the barren areas in the desert in sterile vials. A total of 12 soil samples were collected from different locations in the desert. The actinobacterial strains were recovered by the crowded plate technique on two different media, including glycerol casein KNO_3_ agar (gL^− 1^: glycerol 10, KNO_3_ 2, casein 0.3, NaCl 2, K_2_HPO_4_ 2, MgSO_4_.7H_2_O 0.05, CaCO_3_ 0.02, FeSO_4_.7H_2_O 0.01, agar 18) and actinomycete isolation agar (gL^− 1^: glycerol 5, sodium caseinate 2, sodium propionate 4, asparagine 0.1, K_2_HPO_4_ 0.5, MgSO_4_.7H_2_O 0.1, FeSO_4_.7H_2_O 0.01, agar 18). The purification of isolated actinobacteria strains was done on glucose yeast malt extract agar (gL^− 1^: glucose 4, yeast extract 4, malt extract 10, CaCO_3_ 2, agar 15).

### Taxonomic characterization of actinobacteria strains

The pure cultures of the isolated strains were obtained for taxonomic characterization. The morphological characteristics (color of substrate, aerial mycelia, colony size, and consistency) were analyzed on 10 d old cultures grown at 28 °C on M2 agar (gL^− 1^: glucose 4, yeast extract 4, malt extract 10, CaCO_3_ 2, agar 15). The biochemical and physiological characteristics were studied by following the reported procedures [77]. These included melanin formation, utilization of different sugars as sole carbon source, organic acid formation, hydrolysis of urea, esculin hydrolysis, and arbutin hydrolysis.

The DNA extraction was performed by using the methods described by Li et al. [78]. The 16S rRNA gene sequence was amplified using universal primers (27f: AGAGTTTGATCCTG GCTCAG) and (1492r: 5′-TACGGYTACCTTGTTACGAC-3′) following the method described by Jordan et al. [79]. Sequencing was performed using an ABI PRISMTM 3730XL DNA Analyzer and the pEASY-T1 Cloning Kit (TransGen Biotech) as per the manufacturer’s instructions [30]. EzBioCloud [40, 80] and NCBI (BLAST) [39] was used to compare 16S rRNA gene sequences with the type strains and deposited in GenBank to get the accession numbers.

### Shake flask cultivation and methanolic extraction

The actinobacterial strains were grown in 50 mL of liquid A-medium (gL^− 1^: starch 20, glucose 10, peptone 5, yeast extract 5, NaCl 4, K_2_HPO_4_ 0.5, MgSO_4_.7H_2_O 0.5). The flasks were incubated at 28 °C, 200 rpm in the dark for 10 d. After 10 d, XAD-16 N resin (4%; 2 g per 50 mL) was added to each flask, and the flasks were kept on the shaker (200 rpm) overnight at 28 °C. The cultures containing resin were transferred to the 50 mL vials and centrifuged at 4000 g for 15 min. The pellet (XAD-16/cells) was washed with deionized water, centrifuged, and methanol (20 mL) was added. The methanolic crude extracts were preserved at 4 °C and were used for further biological and chemical screening [28].

### Preliminary screening of actinobacterial methanolic extracts

#### Antimicrobial assay

Microtiter plate assays against Gram-positive bacteria (*Staphylococcus aureus* ATCC 6538, *Micrococcus luteus* NRRL B-287, *Bacillus subtilis* ATCC 6633) Gram-negative bacteria (*Escherichia coli* NRRL B-3708, *Salmonella enterica* ATCC 10708) and yeast (*Saccharomyces cerevisiae* ATCC 204508) test strains were performed in triplicate, following the procedure described by Wang et al. [81].

#### In vitro cytotoxicity assay

The cell line cytotoxicity assays against A549 (human adenocarcinoma non-small-cell lung carcinoma) and PC3 (human prostate cancer) cell lines (ATCC, Manassas, VA, USA) were performed in triplicate by following the previously described procedure [82].

### Chemical profiling

#### Thin-layer chromatography (TLC)

The production and nature of secondary metabolites produced by actinobacteria were analyzed by screening the crude extracts on Polygram SIL G/UV254 TLC plates (Macherey-Nagel & Co., Dueren, Germany), eluted using 10% methanol/dichloromethane. The plates were visualized under UV (254 nm and 365 nm), and were stained with anisaldehyde/H_2_SO_4_ for visualization [83].

#### High-performance liquid chromatography (HPLC-UV/Vis and HPLC-MS)

To visualize the number and concentration of secondary metabolites in the form of chromatographic peaks, HPLC-UV/vis analysis of actinobacteria crude extracts was accomplished using an Agilent 1260 Infinity HPLC system (Agilent Technologies, Santa Clara, CA, USA) equipped with a Luna (Phenomenex, CA, USA) 5 μm C18 (2) 100 Å, LC column (250 × 4.6 mm; solvent A: 0.1% trifluoroacetic acid (TFA)/water, solvent B: Acetonitrile, flow rate: 1.0 mL min^˗1^; 0–30 min, 95–0% A (linear gradient); 30–35 min 0% A; 35–36 min 0–95% A (linear gradient); 36–40 min 95% A. UV-vis inset of full wavelength scan (190–600 nm). The methanolic crude extracts were also analyzed by HPLC-MS via an Agilent 6120 Quadrupole MSD mass spectrometer (Agilent Technologies, Santa Clara, CA, USA) equipped with an Agilent 1200 Series quaternary LC system and an Eclipse XDB-CD18 column (5 μm, 150 × 4.6 mm; solvent A: 0.1% formic acid/water, solvent B: 0.1% formic acid/Acetonitrile); flow rate: 0.5 mL min^− 1^; 0–30 min, 5–100% B; 30–35 min, 100% B; 35–36 min, 100–5% B; 36–40 min, 5% B; Phenomenex NX-C18 column (250 × 4.6 mm, 5 *μ*m); 254 nm. The molecular masses were obtained for both positive and negative ion mode.

#### Scale-up fermentation of anti-gram-negative strain PU-KB10–4 (*Streptomyces griseoviridis*)

The strain PU-KB10–4 was selected as priority strain for scale-up fermentation based on TLC, HPLC, antimicrobial, and cytotoxicity analysis. Particularly because, PU-KB10–4 was the only strain among the 100 bacterial strains surveyed to produce metabolites with anti-Gram-negative activity. The strain was subjected to media optimization using three different media, including A, SG, and M2 (Additional file 42: Fig. S38). While all three media led to similar antimicrobial metabolites, HPLC-UV/vis analysis and antimicrobial activity (Additional file 43: Fig. S39). The seed culture (100 mL of A-media inoculated with freshly grown plate culture) was prepared for scale-up fermentation. Based on media optimization, 10 L of SG-media broth (100 flasks; 100 mL per flask) was prepared and autoclaved (121 °C for 30 min). The autoclaved SG-medium broth (gL^− 1^: glucose 20, yeast extract 5, soytone 10, CaCO_3_ 2, CoCl_2_.6H_2_O 1 mg) flasks were inoculated with an aliquot (1 mL per flask) of seed culture and incubated on shakers at 200 rpm, 28 °C in the dark for 8–10 d. Following growth, the reddish-brown culture broth was combined and centrifuged (RLC-6000 rotator) at 5000 g, 4 °C for 20 min. The supernatant was extracted by mixing with XAD-16 (4% w/v) resin overnight, followed by filtration. The resin was washed with water (5 × 1.2 L) to remove media components and was extracted with methanol (5 × 1 L). The methanolic extract was subsequently evaporated in vacuo to yield 19.7 g of reddish-brown oily extract. The biomass (mycelium) was extracted with methanol (5 × 800 mL) and then evaporated in vacuo and 15.1 g of reddish-brown oily extract was obtained. Both extracts showed a similar set of metabolites based on HPLC and TLC and were therefore combined to give a total of 34.8 g of crude extract.

#### Column fractionation and compound purification

**Fig. 8:**
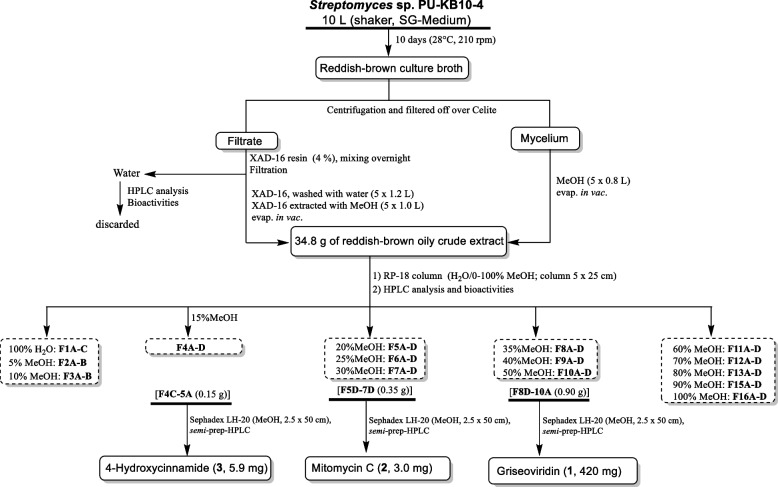
Work-up scheme of PU-KB10–4 (*Streptomyces griseoviridis*)

As highlighted in Fig. 8, the combined extract (34.8 g) was subjected to fractionation using a reverse-phase RP18-column (column 5.0 × 25 cm) eluted with a gradient of H_2_O/0–100%CH_3_OH to give 15 fractions.

All fractions were subjected to TLC and HPLC analysis and were tested for antimicrobial activities and in vitro cytotoxic activity (A549 and PC3) (Additional file 44: Fig. S40). Fractions F4C-5A (0.15 g) were combined based on their similar TLC and HPLC profiles, followed by purification using Sephadex LH-20 (MeOH, 2.5 × 50 cm) and *semi-*preparative HPLC to give compound **3** (5.9 mg). *Semi*-preparative HPLC was done on a Varian (Palo Alto, CA) ProStar Model 210 equipped with a photodiode diode array detector using a Supelco DiscoveryBio wide pore C18 column (21.2 × 250 mm, 10 μm); solvent A: H_2_O/0.025% TFA, solvent B: CH_3_CN; flow rate: 8.0 mL min^− 1^; 0–2 min, 25% B; 2–26 min, 25–100% B; 26–28 min, 100% B; 28–30 min, 100–25% B; 30–32 min, 25% B). Similarly, fractions F5D-7D (0.35 g) were combined to obtain compound **2** (3.0 mg) and fractions F8D-10A (0.90 g) were combined and subjected to purification to get compound **1** (420.0 mg) as the major metabolite (> 42 mg l^˗1^). The remaining fractions and sub-fractions lacked bioactivity and, based on HPLC-MS and TLC profiles, mainly contained media, fats/lipids components.

#### Structure elucidation

Structure elucidation of purified fractions was performed through nuclear magnetic resonance (NMR) spectroscopy. All NMR spectra were recorded using a Varian Inova NMR spectrometers (^1^H, 500 MHz or 400 MHz; ^13^C, 125 MHz or 100 MHz) where *δ*-values were referenced to respective solvent signals [CD_3_OD, *δ*_H_ 3.31 ppm, *δ*_C_ 49.15 ppm; DMSO-*d*_*6*_, *δ*_H_ 2.50 ppm, *δ*_C_ 39.51 ppm] (Palo Alto, CA).

#### X-ray crystallography

Colorless bulk crystals of compound **1** were obtained in CH_3_OH/H_2_O (3:1), [stored at 4 °C for 35 days, then at room temperature (28 °C) for 35 days]. X-ray diffraction data was collected at 90.0 (2) K on a Bruker D8 Venture dual-source diffractometer using graded-multilayer focused Mo*Ka* X-rays. Raw data was integrated, scaled, merged, and corrected for Lorentz-polarization effects using the APEX3 package [Bruker (2016) “APEX3” Bruker-AXS, Madison WI, USA]. Corrections for absorption were applied using SADABS [84]. The structure was solved by direct methods “SHELXT” [85] and refined against F2 by weighted full-matrix least-squares “SHELXL-2018” [86]. Hydrogen atoms were found in difference maps and subsequently placed at calculated positions and refined using a riding model. Water hydrogen atoms, however, were refined freely. Non-hydrogen atoms were refined with anisotropic displacement parameters. The final structure model was checked using an *R*-tensor [87] and Platon/checkCIF [88].

### Statistical analysis

The % cell viability in cytotoxicity assays and % growth inhibition in antimicrobial assays was calculated on Excel, each experiment was performed in triplicate and standard error was calculated.

## Supplementary Information

**Additional file 1: Fig. S1**. Morphological appearance of selected actinobacterial strains (A) strain PU-KB2–2 (*Streptomyces griseoviridis*) (B) strain PU-KB6–10 (*Streptomyces mutabilis*) (C) strain PU-KB8–2 (*Streptomyces djakartensis*) (D) strain PU-KB10–4 (*Streptomyces griseoviridis*) (E) strain PU-KB10–10 (*Streptomyces luteogriseus*) (F) strain PU-KB12–15 (*Streptomyces atrovirens*).

**Additional file 2: Table S1.** Biochemical characterization of actinobacterial strains isolated from Kubuqi Desert, China.

**Additional file 3: Table S2.** 16S rRNA gene sequence analysis and % similarity of the Kubuqi desert strains with various actinobacterial type strains.

**Additional file 4: Table S3.** Microtiter plate assay for antimicrobial activity of all actinobacterial strains against bacteria (Gram-positive and Gram-negative) and yeast along with percentage cytotoxicity against cancer cell lines.

**Additional file 5: Fig. S2.** TLC (CH_2_Cl_2_/10%MeOH) screening of the extracts produced PU-KB strains.

**Additional file 6: Fig. S3.** HPLC/UV analyses of the generated extract produced by *Streptomyces* sp. PU-KB5-11 (A-medium). HPLC-conditions: Detection wavelength 280 and 320 nm; solvent A: H_2_O/0.1% TFA; solvent B: acetonitrile; flow rate: 1.0 mL min^-1^; 0-30 min, 95-0% A (linear gradient); 30-35 min 0% A; 35-36 min 0-95% A (linear gradient); 36-40 min 95% A. UV-vis inset of full wavelength scan (190-600 nm).

**Additional file 7: Fig. S4.** HPLC/UV analyses of the generated extract produced by *Streptomyces* sp. PU-KB10-4 (A-medium). HPLC-conditions: Detection wavelength 320 and 280 nm;; solvent A: H_2_O/0.1% TFA; solvent B: acetonitrile; flow rate: 1.0 mL min^-1^; 0-30 min, 95-0% A (linear gradient); 30-35 min 0% A; 35-36 min 0-95% A (linear gradient); 36-40 min 95% A. UV-vis inset of full wavelength scan (190-600 nm).

**Additional file 8: Fig. S5.** HPLC-MS analysis of PU-KB8-2 crude extract. HPLC-conditions: solvent A: H_2_O/0.1% FA; solvent B: CH_3_CN; flow rate: 0.5 mL min^-1^; 0-30 min, 5-100% B; 30-35 min, 100% B; 35-36 min, 100-5% B; 36-40 min, 5% B; Phenomenex NX-C18 column (250 × 4.6 mm, 5 μm); 254 nm.

**Additional file 9: Fig. S6.** HPLC-MS analysis of PU-KB9-11 crude extract. HPLC-conditions: solvent A: H_2_O/0.1% FA; solvent B: CH_3_CN; flow rate: 0.5 mL min^-1^; 0-30 min, 5-100% B; 30-35 min, 100% B; 35-36 min, 100-5% B; 36-40 min, 5% B; Phenomenex NX-C18 column (250 × 4.6 mm, 5 *μ*m); 254 nm.

**Additional file 10: Fig. S7.** HPLC-MS analysis of PU-KB10-4 crude extract. HPLC-conditions: solvent A: H_2_O/0.1% FA; solvent B: CH_3_CN; flow rate: 0.5 mL min^-1^; 0-30 min, 5-100% B; 30-35 min, 100% B; 35-36 min, 100-5% B; 36-40 min, 5% B; Phenomenex NX-C18 column (250 × 4.6 mm, 5 μm); 254 nm.

**Additional file 11: Fig. S8.** HPLC-MS analysis of PU-KB12-3 crude extract. HPLC-conditions: solvent A: H_2_O/0.1% FA; solvent B: CH_3_CN; flow rate: 0.5 mL min^-1^; 0-30 min, 5-100% B; 30-35 min, 100% B; 35-36 min, 100-5% B; 36-40 min, 5% B; Phenomenex NX-C18 column (250 × 4.6 mm, 5 *μ*m); 254 nm.

**Additional file 12: Figs. S9.** Fermentation cultures of actinomycin D producing strains (A-medium, 10d, 210 rpm, 28 °C). The dark brown culture of strain PU-KB6-7 is due to high concentration of metal-complex siderophores (Fe – complex), which was consistent with the detected peak in HPLC/UV analysis figure at 18.8 min, and the low concentration of actinomycin D at 25.3 min.

**Additional file 13: Fig. S10.** HPLC/UV analyses of the generated extract produced by actinomycin D producing strains (A-medium).

**Additional file 14: Fig. S11.** HPLC-MS analysis of one selected actinomycin D producer crude extract (PU-KB7-8).

**Additional file 15: Fig. S12.** (+)-HRMS of analysis of actinomycin D.

**Additional file 16: Fig. S13.** HPLC analysis of griseoviridin (1). HPLC-conditions: solvent A: H_2_O/0.1% FA; solvent B: CH_3_CN; flow rate: 0.5 mL min^-1^; 0-30 min, 5-100% B; 30-35 min, 100% B; 35-36 min, 100-5% B; 36-40 min, 5% B; Phenomenex NX-C18 column (250 × 4.6 mm, 5 μm); 254 nm. UV-vis inset of full wavelength scan (190-600 nm). (+) and (–)-ESI-MS spectra of griseoviridin **(1)**.

**Additional file 17: Fig. S14.** ^1^H NMR spectrum (CD_3_OD, 400 MHz) of griseoviridin **(1)**.

**Additional file 18: Fig. S15.** ^13^C NMR spectrum (CD_3_OD, 100 MHz) of griseoviridin **(1)**.

**Additional file 19: Fig. S16.** APT NMR spectrum (CD_3_OD, 100 MHz) of griseoviridin **(1)**.

**Additional file 20: Fig. S17.** ^1^H (400 MHz) and ^13^C (100 MHz) NMR spectra of griseoviridin **(1)** in CD_3_OD.

**Additional file 21: Fig. S18.** NOESY spectrum (CD_3_OD, 400 MHz) of griseoviridin **(1)**.

**Additional file 22: Fig. S19.** ^1^H,^1^H-COSY spectrum (CD_3_OD, 400 MHz) of griseoviridin **(1)**.

**Additional file 23: Fig. S20.** HSQC spectrum (CD_3_OD, 400 MHz) of griseoviridin **(1)**.

**Additional file 24: Fig. S21.** HMBC spectrum (CD_3_OD, 400 MHz) of griseoviridin **(1)**.

**Additional file 25: Fig. S22.** TOCSY spectrum (CD_3_OD, 400 MHz) of griseoviridin **(1)**.

**Additional file 26: Fig. S23.** HPLC analysis of mitomycin C (2). HPLC-conditions: solvent A: H_2_O/0.1% FA; solvent B: CH_3_CN; flow rate: 0.5 mL min^-1^; 0-30 min, 5-100% B; 30-35 min, 100% B; 35-36 min, 100-5% B; 36-40 min, 5% B; Phenomenex NX-C18 column (250 × 4.6 mm, 5 μm); 254 nm. UV-vis inset of full wavelength scan (190-600 nm). (+) and (–)-ESI-MS spectra of mitomycin C **(2)**.

**Additional file 27: Fig. S24.** ^1^H NMR spectrum (CD_3_OD, 400 MHz) of mitomycin C **(2)**.

**Additional file 28: Fig. S25.**^13^C NMR spectrum (CD_3_OD, 100 MHz) of mitomycin C **(2)**.

**Additional file 29: Fig. S26.** ^1^H,^1^H-COSY spectrum (CD_3_OD, 500 MHz) of mitomycin C **(2)**.

**Additional file 30: Fig. S27.** ^1^H (400 MHz) and ^13^C (100 MHz) NMR spectra of mitomycin C **(2)** in CD_3_OD.

**Additional file 31 Fig. S28.** HPLC analysis of 4-hydroxycinnamide **(3)**. HPLC-conditions: solvent A: H_2_O/0.1% FA; solvent B: CH_3_CN; flow rate: 0.5 mL min^-1^; 0-30 min, 5-100% B; 30-35 min, 100% B; 35-36 min, 100-5% B; 36-40 min, 5% B; Phenomenex NX-C18 column (250 × 4.6 mm, 5 *μ*m); 254 nm. UV-vis inset of full wavelength scan (190-600 nm). (+) and (–)-ESI-MS spectra of 4-hydroxycinnamide **(3)**.

**Additional file 32: Fig. S29.** ^1^H NMR spectrum (CD_3_OD, 500 MHz) of 4-hydroxycinnamide **(3)**.

**Additional file 33: Fig. S30.** ^13^C NMR spectrum (CD_3_OD, 125 MHz) of 4-hydroxycinnamide **(3)**.

**Additional file 34: Fig. S31.** ^1^H (500 MHz) and ^13^C (125 MHz) NMR spectra of 4-hydroxycinnamide **(3)** in CD_3_OD.

**Additional file 35: Fig. S32.** ^1^H NMR spectrum (DMSO-*d*_6_, 400 MHz) of 4-hydroxycinnamide **(3)**.

**Additional file 36: Fig. S33.** ^13^C NMR spectrum (DMSO-*d*_6_, 100 MHz) of 4-hydroxycinnamide **(3)**.

**Additional file 37: Fig. S34.** ^1^H (400 MHz) and ^13^C (100 MHz) NMR spectra of 4-hydroxycinnamide **(3)** in DMSO-*d*_6_.

**Additional file 38: Fig. S35.** ^1^H,^1^H-COSY spectrum (DMSO-*d*_6_, 400 MHz) of 4-hydroxycinnamide **(3)**.

**Additional file 39. pdf: Fig. S36.** HSQC spectrum (DMSO-*d*_6_, 400 MHz) of 4-hydroxycinnamide **(3)**.

**Additional file 40: Fig. S37.** HMBC spectrum (DMSO-*d*_6_, 400 MHz) of 4-hydroxycinnamide **(3)**.

**Additional file 41..** Physiochemical properties of compounds **1-3**.

**Additional file 42: Fig. S38.** (A) *Streptomyces* sp. PU-KB10-4 grown on a M2-medium agar plate. (B) HPLC/UV analyses of the generated extracts produced by *Streptomyces* sp. PU-KB10-4 using three different media (A-, SG- and M2 media). SG medium was selected for scale-up as 10 L based on the high production of the major metabolite (Compound **1**).

**Additional file 43: Fig. S39.** (A) HPLC/UV analyses of the mycelium and XAD-extracts produced by *Streptomyces* sp. PU-KB10-4. Compound **1** was the major compound of the strain extract (42 mg/L). (B) Antimicrobial activity of crude extracts of PU-KB10–4 grown in three different media: the crude extracts of PU-KB10–4 obtained from A-media, M2- media and SG media showed less activity against Gram-negative bacteria (*Escherichia coli*) and huge zones of inhibition against Gram-positive bacteria (*Staphylococcus aureus*).

**Additional file 44: Fig. S40.** (A and B) Cytotoxicity and antimicrobial analysis of PU-KB10-4 fractions obtained after RP-18 column chromatography (C and D) Cytotoxicity and antimicrobial analysis of PU-KB10-4 after combining the initial column fractions.

## Data Availability

The DNA sequencing datasets generated and analyzed during the current study are available in the National Center for Biotechnology Information (NCBI) repository; GenBank, https://www.ncbi.nlm.nih.gov/ Accession number; MN813066-MN813161. The crystallographic structural data has been submitted to Cambridge Crystallographic Data Centre (CCDC) https://www.ccdc.cam.ac.uk/ as supplementary publication 2055325. All data generated or analyzed during this study are included in this article as supplementary information (Additional files 1, 2, 3, 4, 5, 6, 7, 8, 9, 10, 11, 12, 13, 14, 15, 16, 17, 18, 19, 20, 21, 22, 23, 24, 25, 26, 27, 28, 29, 30, 31, 32, 33, 34, 35, 36, 37, 38, 39, 40, 41, 42, 43 and 44).

## Abbreviations

AMR: Antimicrobial resistance
ATCC: American type culture collection
BLAST: Basic local Alignment search tool
CCDC: Cambridge Crystallographic Data Centre
DNA: Deoxyribonucleic acid
HPLC-MS: High-performance liquid chromatography mass spectrometry
HPLC-UV/vis: High-performance liquid chromatography ultraviolet/visible
NCBI: National center for biotechnology information
NMR: Nuclear magnetic resonance
OD: Optical density
TFA: Trifluoroacetic acid
TLC: Thin layer chromatography

## References

[1] Murray CJ, Ikuta KS, Sharara F, Swetschinski L, Aguilar GR, Gray A, Han C, Bisignano C, Rao P, Wool E (2022). Global burden of bacterial antimicrobial resistance in 2019: a systematic analysis. Lancet.

[2] CDC. Antibiotic resistance threats in the United States, 2019. Atlanta: US Department of Health and Human Services, Centres for Disease Control and Prevention; 2019. https://www.cdc.gov/drugresistance/biggest-threats.html. Accessed 27 Sept 2020.

[3] Long NS, Wells JE, Berry ED, Legako JF, Woerner DR, Loneragan GH, et al. Metaphylactic antimicrobial effects on occurrences of antimicrobial resistance in *Salmonella*, *Escherichia coli*, and *Enterococcus* spp. measured longitudinally from feedlot arrival to harvest in high-risk beef cattle. J App Microbiol. 2022;133(3):1940–55. 10.1111/jam.15691 PMC9546201 35766106

[4] Allsop A, Illingworth R (2002). The impact of genomics and related technologies on the search for new antibiotics. J App Microbiol.

[5] Ossai J, Khatabi B, Nybo SE, Kharel MK (2022). Renewed interests in the discovery of bioactive actinomycete metabolites driven by emerging technologies. J App Microbiol.

[6] Oren A, Garrity GM (2022). Notification that new names of prokaryotes, new combinations, and new taxonomic opinions have appeared in volume 71, part 10 of the IJSEM. Int J Syst Evol Microbiol.

[7] Bérdy J (2012). Thoughts and facts about antibiotics: where we are now and where we are heading. J Antibiot.

[8] Hutchings MI, Truman AW, Wilkinson B (2019). Antibiotics: past, present and future. Curr Opin Microbiol.

[9] Ouchari L, Boukeskasse A, Bouizgarne B, Ouhdouch Y. Antimicrobial potential of actinomycetes isolated from the unexplored hot Merzouga desert and their taxonomic diversity. Biol Open. 2019;8(2):bio035410. 10.1242/bio.035410. 10.1242/bio.035410 PMC6398458 30127092

[10] Das R, Romi W, Das R, Sharma HK, Thakur D (2018). Antimicrobial potentiality of actinobacteria isolated from two microbiologically unexplored forest ecosystems of Northeast India. BMC Microbiol.

[11] Azman A-S, Mawang C-I, Khairat J-E, AbuBakar S. Actinobacteria—a promising natural source of anti-biofilm agents. Int Microbiol. 2019;22(4):403–9. 10.1007/s10123-019-00066-4. 10.1007/s10123-019-00066-4 30847714

[12] Saleem HGM, Aftab U, Sajid I, Abbas Z, Sabri AN. Effect of crude extracts of selected actinomycetes on biofilm formation of *A. schindleri*, *M. aci*, and *B. cereus*. J Basic Microbiol. 2015;55(5):645–51. 10.1002/jobm.201400358 25138589

[13] Gozari M, Bahador N, Jassbi AR, Mortazavi MS, Hamzehei S, Eftekhar E. Isolation, distribution and evaluation of cytotoxic and antioxidant activity of cultivable actinobacteria from the Oman Sea sediments. Acta Oceanol Sin. 2019;38(12):84–90. 10.1007/s13131-019-1515-2.

[14] Davies-Bolorunduro OF, Adeleye IA, Akinleye MO, Wang PG. Anticancer potential of metabolic compounds from marine actinomycetes isolated from Lagos lagoon sediment. J Pharm Anal. 2019;9(3):201–8. 10.1016/j.jpha.2019.03.004. 10.1016/j.jpha.2019.03.004 PMC6598170 31297298

[15] Wongsariya K, Thawai C. Antifungal activity against the growth of Aflatoxin producing fungi from soil Actinobacteria. J Adv Agric Technol. 2019;6(3):200–4.

[16] Gomathi A, Gothandam KM. Investigation of anti-inflammatory and toxicity effects of mangrove-derived *Streptomyces rochei *strain VITGAP173. J Cell Biochem. 2019;120(10):17080–97. 10.1002/jcb.28969. 10.1002/jcb.28969 31104317

[17] Law JW-F, Pusparajah P, Ab Mutalib N-S, Wong SH, Goh B-H. Lee L-H. A review on mangrove actinobacterial diversity: the roles of *Streptomyces* and novel species discovery. Prog Microbes Mol Biol. 2019;2(1).

[18] Thawai C, Kittakoop P, Tanasupawat S, Suwanborirux K, Sriklung K, Thebtaranonth Y. Micromonosporin A, a novel 24-membered polyene lactam macrolide from *Micromonospora* sp. isolated from Peat Swamp Forest. Chem Biodivers. 2004;1(4):640–5. 10.1002/cbdv.200490055 17191875

[19] Sivalingam P, Hong K, Pote J, Prabakar K. Extreme environment *Streptomyces*: potential sources for new antibacterial and anticancer drug leads? Int J Microbiol. 2019;2019:20. 10.1155/2019/5283948 PMC6636559 31354829

[20] Sayed AM, Hassan MH, Alhadrami HA, Hassan HM, Goodfellow M, Rateb ME. Extreme environments: microbiology leading to specialized metabolites. J Applied Microbiol. 2020;128(3):630–57. 10.1111/jam.14386 31310419

[21] Luedemann GM. Geodermatophilus, a new genus of the *Dermatophilaceae* (Actinomycetales). J Bacteriol. 1968;96(5):1848–58. 10.1128/jb.96.5.1848-1858.1968. 10.1128/jb.96.5.1848-1858.1968 PMC315248 5726312

[22] Garrity G, Heimbuch B, Gagliardi M. Isolation of zoosporogenous actinomycetes from desert soils. J Ind Microbiol Biotechnol. 1996;17(3–4):260–67. 10.1007/bf01574700.

[23] Kurapova A, Zenova G, Sudnitsyn I, Kizilova A, Manucharova N, Norovsuren Z, et al. Thermotolerant and thermophilic actinomycetes from soils of Mongolia desert steppe zone. Microbiol. 2012;81(1):98–108. 10.1134/s0026261712010092. 22629687

[24] Meklat A, Bouras N, Zitouni A, Mathieu F, Lebrihi A, Schumann P, Spröer C, Klenk H-P, Sabaou N: *Actinopolyspora mzabensis* sp. nov., a halophilic actinomycete isolated from an Algerian Saharan soil. Int J Syst Evol Microbiol. 2013;63:3787–92. 10.1099/ijs.0.046649-0. 10.1099/ijs.0.046649-0 23667146

[25] Chanal A, Chapon V, Benzerara K, Barakat M, Christen R, Achouak W, et al. The desert of Tataouine: an extreme environment that hosts a wide diversity of microorganisms and radiotolerant bacteria. Environ Microbiol. 2006;8(3):514–25. 10.1111/j.1462-2920.2005.00921.x. 10.1111/j.1462-2920.2005.00921.x 16478457

[26] Goodfellow M, Nouioui I, Sanderson R, Xie F, Bull AT. Rare taxa and dark microbial matter: novel bioactive actinobacteria abound in Atacama Desert soils. Antonie Van Leeuwenhoek. 2018;111(8):1315–32. 10.1007/s10482-018-1088-7. 10.1007/s10482-018-1088-7 29721711

[27] Fatima A, Aftab U, Shaaban KA, Thorson JS, Sajid I. Spore forming Actinobacterial diversity of Cholistan Desert Pakistan: Polyphasic taxonomy, antimicrobial potential and chemical profiling. BMC Microbiol. 2019;19(1):1–17. 10.1186/s12866-019-1414-x. 10.1186/s12866-019-1414-x PMC6387500 30795744

[28] Cheema MT, Ponomareva LV, Liu T, Voss SR, Thorson JS, Shaaban KA, Sajid I. Taxonomic and metabolomics profiling of actinobacteria strains from Himalayan collection sites in Pakistan. Curr Microbiol. 2021;78(8):3044–57. 10.1007/s00284-021-02557-y PMC10716794 34125273

[29] Zhang Y, Cheema MT, Ponomareva LV, Ye Q, Liu T, Sajid I, Rohr Jr, She Q-B, Voss SR, Thorson JS, Himalaquinones A–G. Angucyclinone-derived metabolites produced by the Himalayan isolate *Streptomyces* sp. PU-MM59. J Nat Prod. 2021;84(7):1930–40. 10.1021/acs.jnatprod.1c00192 PMC8565601 34170698

[30] Liu S-W, Ye J-J, Lu Q-P, Cheema MT, Abbas M, Huang D-L, Sajid I, Sun C-H (2020). Motilibacter deserti sp. nov. and Motilibacter aurantiacus sp. nov., two novel actinobacteria isolated from soil of Cholistan Desert and emended description of the genus Motilibacter. Syst Appl Microbiol.

[31] Rechtschaffen D How China's Growing Deserts Are Choking The Country 2017 America https://www.forbes.com/sites/danielrechtschaffen/2017/09/18/how-chinas-growing-deserts-are-choking-the-country/?sh=49ad4e65d1b9 Accessed on 22 May 2021.

[32] Wang L, Lee X, Schultz N, Chen S, Wei Z, Fu C, et al. Response of surface temperature to afforestation in the Kubuqi Desert, Inner Mongolia. J Geophys Res. 2018;123(2):948–64. 10.1007/s12145-020-00467-4.

[33] Dong X, Chen Z, Wu M, Hu C. Long time series of remote sensing to monitor the transformation research of Kubuqi Desert in China. Earth Sci Inf. 2020;13:795–809. 10.1007/s12145-020-00467-4.

[34] Saitou N, Nei M. The neighbor-joining method: a new method for reconstructing phylogenetic trees. Mol Biol Evol. 1987;4(4):406–25. 10.1093/oxfordjournals.molbev.a040454. 10.1093/oxfordjournals.molbev.a040454 3447015

[35] Felsenstein J. Confidence limits on phylogenies: an approach using the bootstrap. Evolution. 1985;39(4):783–91. 10.2307/2408678. 10.1111/j.1558-5646.1985.tb00420.x 28561359

[36] Tamura K, Nei M, Kumar S (2004). Prospects for inferring very large phylogenies by using the neighbor-joining method. Proc Natl Acad Sci.

[37] Letunic I, Bork P (2021). Interactive tree of life (iTOL) v5: an online tool for phylogenetic tree display and annotation. Nucleic Acids Res.

[38] Oren A, Garrity GM (2021). Valid publication of the names of forty-two phyla of prokaryotes. Int J Syst Evol Microbiol.

[39] NCBI (BLAST) https://blast.ncbi.nlm.nih.gov/Blast.cgi Accessed on 10 June 2019.

[40] EZBioCloud https://www.ezbiocloud.net/ Accessed on 7 June 2019.

[41] Evans A (1961). Mitomycin C. Cancer Chemother Rep.

[42] Hossenlopp C. Experimental treatment of Cancer with mitomycin C alone or in combination with carzinophilin. J Antibiot (Tokyo). 1961;14(5):289–97. 10.11554/antibioticsa.14.5_289. 14036368

[43] Hata T, Hossenlopp C, Takita H (1961). Studies on mitomycin C, especially method of administration. Cancer Chemother Rep.

[44] Gourevitch A, Rossomano V, Lein J (1961). Differential assay for mitomycin A and C. Antibiot Chemother (Northfield, Ill).

[45] Bergsagel DE. Phase II trials of mitomycin C, AB-100, NSC-1026, L-sarcolysin, and meta-sarcolysin, in the treatment of multiple myeloma. Cancer Chemother Rep. 1962;16:261–66. 13867796

[46] Tomasz M, Chowdary D, Lipman R, Shimotakahara S, Veiro D, Walker V, et al. Reaction of DNA with chemically or enzymatically activated mitomycin C: isolation and structure of the major covalent adduct. National Acad Sci. 1986;83(18):6702–6. 10.1073/pnas.83.18.6702. 10.1073/pnas.83.18.6702 PMC386577 3018744

[47] Verweij J, Pinedo HM. Mitomycin C: mechanism of action, usefulness and limitations. Anti-Cancer Drugs. 1990;1(1):5–13. 10.1097/00001813-199010000-00002. 2131038

[48] Gibson N, Phillips RM, Ross D (1994). Mitomycin C. Cancer Chemother Biol Res Modifiers.

[49] Bradner W. Mitomycin C: a clinical update. Cancer Treat Rev. 2001;27(1):35–50. 10.1053/ctrv.2000.0202. 10.1053/ctrv.2000.0202 11237776

[50] Galm U, Hager MH, Van Lanen SG, Ju J, Thorson JS, Shen B: Antitumor antibiotics: bleomycin, enediynes, and mitomycin. Chem Rev. 2005;105(2)739–58. 10.1002/chin.200524280. 10.1021/cr030117g 15700963

[51] Luo M, , Zhang H, To KK, Wu S, Chen Z, Liang S, Fu L: Mitomycin C enhanced the efficacy of PD-L1 blockade in non-small cell lung cancer. Signal Transduct Targeted Ther. 2020;5(1):1–14. 10.1038/s41392-020-0200-4. 10.1038/s41392-020-0200-4 PMC7452895 32855386

[52] Mearza AA, Aslanides IM. Uses and complications of mitomycin C in ophthalmology. Expert Opin Drug Saf. 2007;6(1):27–32. 10.1517/14740338.6.1.27. 10.1517/14740338.6.1.27 17181449

[53] Jayaraman G, Bhaskaran R, Yu C, Young JJ, Jung LJ, Liu WT, et al. Three-dimensional structure in solution of griseoviridin, a group a antibiotic. Biochim Biophys Acta (BBA)-General Subjects. 1994;1201(2):149–56. 10.1016/0304-4165(94)90035-3. 10.1016/0304-4165(94)90035-3 7947926

[54] Bycroft BW, King TJ. Revised constitution, absolute configuration, and conformation of griseoviridin, a modified cyclic peptide antibiotic. J Chem Soc, Perkin Trans I. 1976;(19):1996–2004. 1033191

[55] Birnbaum GI, Hall SR. Structure of the antibiotic griseoviridin. J Am Chem Soc. 1976;98(7):1926–31. 10.1021/ja00423a046. 10.1021/ja00423a046 1254852

[56] Wichner D, Idris H, Houssen WE, McEwan AR, Bull AT, Asenjo JA, et al. Isolation and anti-HIV-1 integrase activity of lentzeosides A–F from extremotolerant lentzea sp. H45, a strain isolated from a high-altitude Atacama Desert soil. J Antibiotics. 2017;70(4):448–53. 10.1038/ja.2016.78. 10.1038/ja.2016.78 27353167

[57] Yassin A, Rainey F, Brzezinka H, Jahnke K-D, Weissbrodt H, Budzikiewicz H, et al. Lentzea gen. Nov., a new genus of the order Actinomycetales. Int J Syst Evol Microbiol. 1995;45(2):357–63. 10.1099/00207713-45-2-357. 10.1099/00207713-45-2-357 7537071

[58] Rateb ME, Ebel R, Jaspars M. Natural product diversity of actinobacteria in the Atacama Desert. Antonie Van Leeuwenhoek. 2018;111(8):1467–77. 10.1007/s10482-018-1030-z. 10.1007/s10482-018-1030-z 29445902

[59] Hozzein WN, Rabie W, Ali MIA. Screening the Egyptian desert actinomycetes as candidates for new antimicrobial compounds and identification of a new desert *Streptomyces* strain. African J Biotechnol. 2011;10(12):2295–301. 10.5897/AJB10.1973.

[60] Nithya K, Muthukumar C, Duraipandiyan V, Dhanasekaran D, Thajuddin N. Diversity and antimicrobial potential of culturable actinobacteria from desert soils of Saudi Arabia. J Pharm Sci Res. 2015;7(3):117.

[61] Abdelkader MS, Philippon T, Asenjo JA, Bull AT, Goodfellow M, Ebel R, et al. Asenjonamides A–C, antibacterial metabolites isolated from *Streptomyces asenjonii* strain KNN 42. f from an extreme-hyper arid Atacama desert soil. J Antibiot. 2018;71(4):425–31. 10.1038/s41429-017-0012-0 29362461

[62] Zhang W, Wei S, Zhang J, Wu W. Antibacterial activity composition of the fermentation broth of *Streptomyces djakartensis* NW35. Molecules. 2013;18(3):2763–68. 10.3390/molecules18032763 PMC6270429 23455667

[63] Susanti AE, Ratnakomala S, Mangunwardoyo W, Lisdiyanti P. Antimicrobial Activity of Lichens-Associated Actinomycetes Strain LC-23. In: Proceedings of the 2019 9th International Conference on Bioscience, Biochemistry and Bioinformatics; 2019. p. 91–6.

[64] Hosny A-EM, Sheir DH, El-Diwany AI, Abdelwahed NA, Fallarero A, Vuorela PM. Production and characterization of antimicrobial compound produced by *Streptomyces atrovirens* H33. Biolife. 2015;3(2):476–82. 10.17812/blj2015.32.17.

[65] Bartz Q. Griseoviridin and viridogrisein: isolation and characterization. Antibiotics Ann. 1955;1954:777–83.

[66] Vazquez D (1967). The streptogramin family of antibiotics. Mechanism of Action.

[67] Gu JY, Han WJ, Qi FF, Li J, Sun SW, Zhu TJ, et al. Two new 23-membered macrolactones from a terrestrial bacterium, Streptomyces sp. IMBJ01. Helv Chim Acta. 2011;94(8):1448–53.

[68] Hosoda K, Koyama N, Kanamoto A, Tomoda H. Discovery of nosiheptide, griseoviridin, and etamycin as potent anti-mycobacterial agents against Mycobacterium avium complex. Molecules. 2019;24(8):1495. 10.3390/molecules24081495. 10.3390/molecules24081495 PMC6514863 30995807

[69] Xie Y, Ma J, Qin X, Li Q, Ju J. Identification and utilization of two important transporters: SgvT1 and SgvT2, for griseoviridin and viridogrisein biosynthesis in *Streptomyces griseoviridis*. Microb Cell Factories. 2017;16(1):1–10. 10.1186/s12934-017-0792-8. 10.1186/s12934-017-0792-8 PMC5655939 29065880

[70] Kwan BW, Chowdhury N, Wood TK. Combatting bacterial infections by killing persister cells with mitomycin C. Environ Microbiol. 2015;17(11):4406–14. 10.1111/1462-2920.12873. 10.1111/1462-2920.12873 25858802

[71] Chowdhury N, Wood TL, Martínez-Vázquez M, García-Contreras R, Wood TK. DNA-crosslinker cisplatin eradicates bacterial persister cells. Biotechnol Bioeng. 2016;113(9):1984–92. 10.1002/bit.25963. 10.1002/bit.25963 26914280

[72] Escobar IE, White A, Kim W, Mylonakis E. New antimicrobial bioactivity against multidrug-resistant gram-positive Bacteria of kinase inhibitor IMD0354. Antibiot. 2020;9(10):665. 10.3390/antibiotics9100665. 10.3390/antibiotics9100665 PMC7601562 33019726

[73] Kim CS, Kim KH, Lee KR (2014). Phytochemical constituents of the leaves of Hosta longipes. Nat Prod Sci.

[74] Wang CY, Jang H-J, Han YK, Su XD, Lee SW, Rho M-C, Wang H-S, Yang SY, Kim YH (2018). Alkaloids from Tetrastigma hemsleyanum and their anti-inflammatory effects on LPS-induced RAW264. 7 cells. Molecules.

[75] Shiraishi T, Domoto T, Imai N, Shimada Y, Watanabe K. Specific inhibitors of tyrosine-specific protein kinase, synthetic 4-hydroxycinnamamide derivatives. Biochem Biophys Res Commun. 1987;147(1):322–8. 10.1016/s0006-291x(87)80124-9 2820397

[76] Vargas-Tah A, Gosset G (2015). Production of cinnamic and p-hydroxycinnamic acids in engineered microbes. Front Bioengineer Biotechnol.

[77] Shirling ET, Gottlieb D. Methods for characterization of Streptomyces species1. Int J Syst Evol Microbiol. 1966;16(3):313–40. 10.1099/00207713-16-3-313.

[78] Li W-J, Xu P, Schumann P, Zhang Y-Q, Pukall R, Xu L-H, et al. *Georgenia ruanii* sp. nov., a novel actinobacterium isolated from forest soil in Yunnan (China), and emended description of the genus Georgenia. Int J Syst Evol Microbiol. 2007;57(7):1424–1428. 10.1099/ijs.0.64749-0 17625169

[79] Jordan EM, Thompson FL, Zhang X-H, Li Y, Vancanneyt M, Kroppenstedt RM, Priest FG, Austin B (2007). Sneathiella chinensis gen. Nov., sp. nov., a novel marine alphaproteobacterium isolated from coastal sediment in Qingdao, China. Int J Syst Evol Microbiol.

[80] Yoon S-H, Ha S-M, Kwon S, Lim J, Kim Y, Seo H, Chun J (2017). Introducing EzBioCloud: a taxonomically united database of 16S rRNA gene sequences and whole-genome assemblies. Int J Syst Evol Microbiol.

[81] Wang X, Shaaban KA, Elshahawi SI, Ponomareva LV, Sunkara M, Copley GC, et al. Mullinamides a and B, new cyclopeptides produced by the Ruth Mullins coal mine fire isolate *Streptomyces* sp. RM-27–46. J antibiot (Tokyo). 2014;67(8):571–5. 10.1038/ja.2014.37 PMC4146655 24713874

[82] Wang X, Shaaban KA, Elshahawi SI, Ponomareva LV, Sunkara M, Zhang Y, Copley GC, Hower JC, Morris AJ, Kharel MK (2013). Frenolicins C–G, pyranonaphthoquinones from Streptomyces sp. RM-4-15. J Nat Prod.

[83] Saleem M, Cheema M, Hassan A, Shaukat S. Sajid I: Endophytes and plant extracts of *Carica papaya* Linn. Exhibit promisisng antibacterial and in-vitro antitmor activity. J Anim Plant Sci. 2020;30(4):1037–46. 10.36899/japs.2020.4.0118.

[84] Krause L, Herbst-Irmer R, Sheldrick GM, Stalke D. Comparison of silver and molybdenum microfocus X-ray sources for single-crystal structure determination. J Appl Crystallogr. 2015;48(1):3–10. 10.1107/s1600576714022985. 10.1107/S1600576714022985 PMC4453166 26089746

[85] Sheldrick GM. SHELXT–integrated space-group and crystal-structure determination. Acta Crystallogr A Found Adv. 2015;71(1):3–8. 10.1107/s2053273314026370. 10.1107/S2053273314026370 PMC4283466 25537383

[86] Sheldrick GM. Crystal structure refinement with SHELXL. Acta Crystallogr C Struct Chem. 2015;71(1):3–8. 10.1107/S2053229614024218. 10.1107/S2053229614024218 PMC4294323 25567568

[87] Parkin S (2000). Expansion of scalar validation criteria to three dimensions: the R tensor. Acta Crystallogr Sect A Found Crystallogr.

[88] Spek AL. Structure validation in chemical crystallography. Acta Cryst. 2009;65(2):148–55. 10.1107/S090744490804362X. 10.1107/S090744490804362X PMC2631630 19171970

