# Supplementary material for: Bioprospecting of desert actinobacteria with special emphases on griseoviridin, mitomycin C and a new bacterial metabolite producing Streptomyces sp. PU-KB10–4
Source: BMC Microbiol. 2023 Mar 15;23:69. doi: 10.1186/s12866-023-02770-8 (PMC10015687; doi:10.1186/s12866-023-02770-8)
Supplement: Supplementary file 1 — Additional file 1: Fig. S1. Morphological appearance of selected actinobacterial strains (A) strain PU-KB2–2 (Streptomyces griseoviridis) (B) strain PU-KB6–10 (Streptomyces mutabilis) (C) strain PU-KB8–2 (Streptomyces djakartensis) (D) strain PU-KB10–4 (Streptomyces griseoviridis) (E) strain PU-KB10–10 (Streptomyces luteogriseus) (F) strain PU-KB12–15 (Streptomyces atrovirens). [file 12866_2023_2770_MOESM1_ESM.pdf]

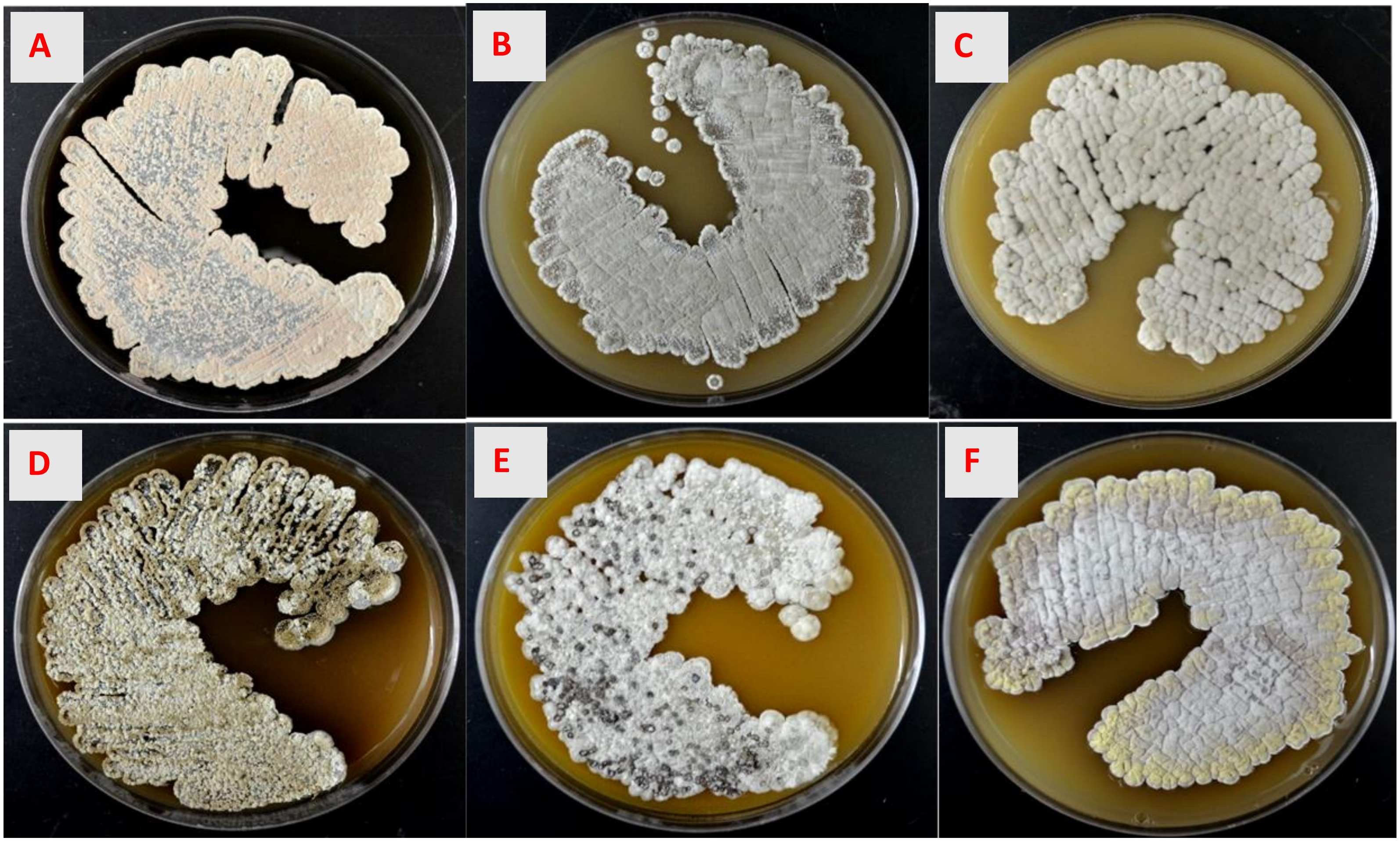

Figure S1: Morphological appearance of selected actinobacterial strains (**A**) strain PU-KB2-2 (*Streptomyces griseoviridis*) (**B**) strain PU-KB6-10 (*Streptomyces mutabilis*) (**C**) strain PU-KB8-2 (*Streptomyces djakartensis*) (**D**) strain PU-KB10-4 (*Streptomyces griseoviridis*) (**E**) strain PU-KB10-10 (*Streptomyces luteogriseus*) (**F**) strain PU-KB12-15 (*Streptomyces atrovirens*).
