# Supplementary material for: Bioprospecting of desert actinobacteria with special emphases on griseoviridin, mitomycin C and a new bacterial metabolite producing Streptomyces sp. PU-KB10–4
Source: BMC Microbiol. 2023 Mar 15;23:69. doi: 10.1186/s12866-023-02770-8 (PMC10015687; doi:10.1186/s12866-023-02770-8)
Supplement: Supplementary file 2 — Additional file 2: Table S1. Biochemical characterization of actinobacterial strains isolated from Kubuqi Desert, China. [file 12866_2023_2770_MOESM2_ESM.pdf]

Table S1: Biochemical characterization of actinobacterial strains isolated from Kubuqi Desert, China

Strains	Melanin Production	Hydrolysis		Utilization of organic acids		Organic acid formation	Oxalate utilization	Utilization of sugars						
		**E**	**U**	**C**	**M**			**Gl**	**Dx**	**Mt**	**Ma**	**Su**	**Fr**	**Ar**
PU-KB1-3	+	+	+	+	+	++	+	+	+	+	++	+	+	+
PU-KB1-4	+	+	+	+	++	++	+	+	-	+	+	-	+	-
PU-KB1-5	+	+	+	+	+	+	+	+	-	+	+	+	+	+
PU-KB1-6	+	-	+	++	+	+	-	++	+	+	++	+	-	-
PU-KB1-7	+	+	+	+	+	++	+	+	+	+	+	+	-	+
PU-KB2-1	+	+	+	+	+	+	+	+	-	+	+	+	+	+
PU-KB2-2	+	+	+	+	+	+	+	++	+	+	+	-	-	-
PU-KB2-3	+	+	+	+	+	+	+	++	+	-	+	+	-	+
PU-KB2-4	+	+	+	+	+	+	+	++	+	-	+	+	-	+
PU-KB2-11	+	+	+	+	+	++	+	++	-	+	+	+	-	+
PU-KB3-1	+	+	+	+	++	++	+	++	+	-	+	+	-	+
PU-KB3-2	+	+	+	++	++	++	+	+	-	+	+	-	+	-
PU-KB3-3	+	+	+	+	+	++	+	++	++	+	+	+	+	+
PU-KB3-5	+	+	+	++	++	++	+	++	+	+	++	-	+	+
PU-KB3-6	+	+	+	+	+	++	+	++	++	+	+	+	+	+
PU-KB5-1	+	+	+	++	+	++	+	++	+	+	++	+	+	+
PU-KB5-2	+	+	+	+	+	-	+	+	+	+	+	+	+	+
PU-KB5-4	-	-	-	++	-	++	+	++	-	+	+	-	+	+
PU-KB5-5	+	+	+	+	++	+	+	+	+	-	+	-	+	-
PU-KB5-7	+	+	+	++	++	++	+	++	+	+	+	+	+	+
PU-KB5-15	+	+	+	+	+	+	+	++	+	-	+	-	+	-
PU-KB5-17	+	++	+	+	-	++	-	++	+	+	+	+	-	+
PU-KB5-19	+	+	+	+	++	+	+	+	+	-	+	-	+	-
PU-KB5-22	+	+	+	++	+	++	+	++	+	+	++	-	+	+
PU-KB5-25	+	+	+	++	+	++	+	+	+	+	++	+	+	+
PU-KB6-2	+	+	+	++	++	++	+	+	-	+	+	+	+	-
PU-KB6-3	+	+	+	++	++	++	-	++	+	-	+	+	+	-
PU-KB6-4	+	+	+	++	++	++	+	+	-	+	+	+	+	-
PU-KB6-7	+	+	+	+	+	+	+	+	-	+	+	+	+	+
PU-KB6-8	+	+	+	++	++	++	+	++	+	+	+	+	+	-
PU-KB6-9	+	++	+	+	++	++	+	+	-	+	+	-	+	-
PU-KB6-10	+	-	+	++	+	++	+	++	-	+	+	+	+	+
PU-KB6-13	+	+	+	+	++	++	+	+	+	+	+	-	+	+
PU-KB6-14	+	+	+	++	++	++	+	++	-	+	+	+	+	+
PU-KB7-1	+	++	+	+	++	++	+	++	-	+	++	+	+	+
PU-KB7-2	+	-	+	+	-	++	-	++	-	+	++	+	-	-
PU-KB7-4	+	+	+	++	++	+	+	++	-	+	+	+	+	-
PU-KB7-5	+	-	+	+	++	+	+	++	+	+	+	+	+	+
PU-KB7-6	+	-	+	+	++	+	+	++	+	-	+	-	+	-
PU-KB7-7	+	+	+	+	+	+	+	++	-	+	+	+	+	+
PU-KB7-8	+	+	+	+	+	++	+	++	-	+	+	+	+	+
PU-KB7-9	+	-	-	+	+	-	+	++	++	+	-	-	+	+
PU-KB7-10	+	-	+	+	++	+	+	++	+	+	+	+	+	+
PU-KB7-13	+	+	+	++	++	+	+	+	+	+	+	+	+	-
PU-KB8-1	+	+	+	++	++	-	+	++	+	+	-	+	-	+
PU-KB8-2	+	+	+	+	++	+	-	++	++	-	-	+	-	-

PU-KB9-1	+	-	+	+	++	-	+	+	++	+	+	-	+	+
PU-KB9-2	+	++	+	++	+	++	+	++	-	+	++	-	+	-
PU-KB9-3	+	-	+	+	++	-	+	+	++	+	+	-	+	+
PU-KB9-5	+	+	+	-	++	++	+	++	-	-	+	-	+	-
PU-KB9-7	+	-	+	+	++	+	+	++	+	-	+	-	+	-
PU-KB9-8	+	-	+	+	++	+++	+	+	++	+	+	-	+	+
PU-KB9-9	+	+	+	-	++	+	+	+	+	+	++	-	-	+
PU-KB9-10	+	+	+	+	+	+	+	-	-	-	+	-	-	-
PU-KB9-11	+	-	+	+	++	+++	+	+	++	+	+	-	+	+
PU-KB9-12	+	+	+	+	++	++	+	++	-	+	++	+	+	+
PU-KB9-13	+	+	+	+	+	+	+	+	-	+	+	+	+	+
PU-KB10-1	+	++	+	++	-	++	+	++	+	+	+	+	+	+
PU-KB10-2	+	+	+	+	+	++	+	+	-	+	+	+	+	+
PU-KB10-3	+	+	+	-	-	+	+	+	-	+	+	+	+	+
PU-KB10-4	+	+	+	+	+	++	+	++	+	+	+	+	+	+
PU-LB10-5	+	++	+	+	++	++	+	+	-	+	+	-	+	-
PU-KB10-6	+	-	+	++	+	++	+	++	-	+	+	+	+	+
PU-KB10-7	+	+	+	+	-	+	+	++	+	+	++	+	-	+
PU-KB10-8	+	+	+	++	++	++	+	+	-	+	+	+	+	-
PU-KB10-11	+	+	+	+	+	++	+	++	+	+	+	+	+	+
PU-KB10-13	+	-	+	-	-	++	+	+	+	+	+	+	+	-
PU-KB11-2	+	+	+	-	-	+	+	+	-	+	+	+	+	+
PU-KB11-3	-	-	+	-	+	+	+	++	+	+	+	-	+	+
PU-KB11-4	+	++	+	-	+	++	-	++	+	+	++	-	+	+
PU-KB11-5	+	+	+	+	+	++	-	+	+	-	++	+	-	+
PU-KB12-1	+	+	+	+	-	++	+	++	+	+	++	-	-	+
PU-KB12-2	+	+	+	++	++	++	+	++	+	+	++	+	+	-
PU-KB12-3	+	-	+	++	++	+	+	+	+	+	++	+	+	-
PU-KB12-4	+	+	+	+	++	++	+	++	-	+	++	+	+	-
PU-KB12-5	+	+	+	+	+	+	-	+	-	+	+	-	+	+
PU-KB12-7	+	+	+	+	-	++	+	++	+	+	++	-	-	+
PU-KB12-8	+	-	+	+	++	+++	+	+	++	+	+	-	+	+
PU-KB12-9	+	+	+	+	-	++	+	++	-	+	++	+	+	-
PU-KB12-10	+	+	-	-	+	-	+	++	+	+	+	+	+	-
PU-KB12-12	+	+	+	++	++	++	+	+	+	+	++	-	+	+
PU-KB12-13	+	-	+	++	++	+	+	++	+	+	++	+	-	-
PU-KB12-15	+	+	+	+	+	+	+	++	-	+	+	+	+	+
PU-KB12-16	+	-	+	++	++	++	-	++	+	+	++	+	+	-
PU-KB12-17	+	+	+	+	-	++	+	++	+	+	++	-	-	+

Key: *E* Esculin, *U* Urea, *C* Trisodium citrate, *M* sodium Malanate, *Gl* Glucose, *Dx* D-xylose, *Mt* Mannitol, *Ma* Mannose, *Su* Sucrose, *Fr* Fructose, *A*Arabinose, (++) = strong positive, (+) = positive, (-) = negative
