# Supplementary material for: Bioprospecting of desert actinobacteria with special emphases on griseoviridin, mitomycin C and a new bacterial metabolite producing Streptomyces sp. PU-KB10–4
Source: BMC Microbiol. 2023 Mar 15;23:69. doi: 10.1186/s12866-023-02770-8 (PMC10015687; doi:10.1186/s12866-023-02770-8)
Supplement: Supplementary file 3 — Additional file 3: Table S2. 16S rRNA gene sequence analysis and % similarity of the Kubuqi desert strains with various actinobacterial type strains. [file 12866_2023_2770_MOESM3_ESM.pdf]

Table S2: 16S rRNA gene sequence analysis and % similarity of the Kubuqi desert strains with various actinobacterial type strains

Strains	Sequence size (base pair)	GenBank accession numbers	Closely related type strains	Similarity (%)
PU-KB1-1	940	MN813066	*Streptomyces xinjiangensis*	99.57
PU-KB1-2	1004	MN813067	*Streptomyces kurssanovii*	99.00
PU-KB1-4	1042	MN813068	*Streptomyces cellostaticus*	99.71
PU-KB1-5	1061	MN813069	*Streptomyces iakyrus*	100.0
PU-KB1-6	1063	MN813070	*Streptomyces pseudovenezuelae*	99.15
PU-KB1-7	1015	MN813071	*Streptomyces yokosukanensis*	99.11
PU-KB1-9	960	MN813072	*Streptomyces xinjiangensis*	99.38
PU-KB1-10	955	MN813073	*Streptomyces griseoviridis*	99.90
PU-KB1-11	956	MN813074	*Streptomyces misionensis*	99.90
PU-KB2-1	946	MN813075	*Streptomyces coerulescens*	99.37
PU-KB2-2	954	MN813076	*Streptomyces griseoviridis*	99.58
PU-KB2-3	954	MN813077	*Streptomyces atrovirens*	99.50
PU-KB2-4	911	MN813078	*Streptomyces atrovirens*	99.89
PU-KB3-1	910	MN813079	*Streptomyces atrovirens*	99.89
PU-KB3-2	960	MN813080	*Streptomyces atrovirens*	99.90
PU-KB3-3	900	MN813081	*Streptomyces hawaiiensis*	99.33
PU-KB3-4	900	MN813082	*Streptomyces fumanus*	99.56
PU-KB3-5	780	MN813083	*Streptomyces bungoensis*	100.0
PU-KB3-6	873	MN813084	*Streptomyces hawaiiensis*	99.54
PU-KB5-1	835	MN813085	*Streptomyces atrovirens*	99.88
PU-KB5-2	834	MN813086	*Streptomyces paradoxus*	100.0
PU-KB5-3	900	MN813087	*Streptomyces atrovirens*	99.82
PU-KB5-4	818	MN813088	*Pseudomonas jessenii*	99.76
PU-KB5-5	811	MN813089	*Streptomyces olivaceoviridis*	99.51
PU-KB5-6	842	MN813090	*Streptomyces atrovirens*	99.05
PU-KB5-8	858	MN813091	*Streptomyces hydrogenans*	99.53
PU-KB5-9	761	MN813092	*Streptomyces badius*	99.47
PU-KB5-10	833	MN813093	*Streptomyces paradoxus*	99.88
PU-KB5-11	732	MN813094	*Streptomyces asenjonii*	99.73
PU-KB5-13	740	MN813095	*Lentzea albida*	99.19
PU-KB5-14	787	MN813096	*Lentzea albida*	99.24
PU-KB5-17	840	MN813097	*Streptomyces atrovirens*	99.88
PU-KB5-18	876	MN813098	*Streptomyces atrovirens*	99.32
PU-KB5-19	673	MN813099	*Streptomyces olivaceoviridis*	99.41
PU-KB5-20	819	MN813100	*Streptomyces olivaceoviridis*	99.51
PU-KB5-21	728	MN813101	*Streptomyces bobili*	100.0
PU-KB5-22	797	MN813102	*Lentzea albida*	99.25
PU-KB5-24	849	MN813103	*Lentzea albida*	99.29
PU-KB5-25	911	MN813104	*Streptomyces lateritius*	99.78
PU-KB6-1	600	MN813105	*Streptomyces deserti*	100.0
PU-KB6-2	840	MN813106	*Streptomyces atrovirens*	99.64
PU-KB6-3	887	MN813107	*Streptomyces ambofaciens*	99.89
PU-KB6-4	876	MN813108	*Streptomyces atrovirens*	99.75
PU-KB6-6	866	MN813109	*Streptomyces rochei*	100.0
PU-KB6-7	822	MN813110	*Streptomyces atrovirens*	99.88
PU-KB6-8	936	MN813111	*Streptomyces atrovirens*	99.89

PU-KB6-9	873	MN813112	*Streptomyces rochei*	100.0
PU-KB6-10	826	MN813113	*Streptomyces mutabilis*	99.88
PU-KB6-11	840	MN813114	*Streptomyces atrovirens*	99.29
PU-KB6-13	863	MN813115	*Streptomyces atrovirens*	99.88
PU-KB7-2	857	MN813116	*Streptomyces indoligenes*	99.77
PU-KB7-3	785	MN813117	*Streptomyces paradoxus*	99.87
PU-KB7-4	745	MN813118	*Streptomyces atrovirens*	99.87
PU-KB7-5	876	MN813119	*Streptomyces viridochromogenes*	99.65
PU-KB7-6	848	MN813120	*Streptomyces levis*	100.0
PU-KB7-7	840	MN813121	*Streptomyces atrovirens*	99.88
PU-KB7-8	869	MN813122	*Streptomyces atrovirens*	99.88
PU-KB7-10	903	MN813123	*Streptomyces viridochromogenes*	99.67
PU-KB8-1	898	MN813124	*Streptomyces hawaiiensis*	99.67
PU-KB8-2	1391	MN813125	*Streptomyces djakartensis*	99.50
PU-KB9-1	809	MN813126	*Streptomyces paradoxus*	100.0
PU-KB9-3	819	MN813127	*Streptomyces paradoxus*	100.0
PU-KB9-4	804	MN813128	*Streptomyces Albogriseolus*	100.0
PU-KB9-5	795	MN813129	*Streptomyces achromogenes subsp. Achromogenes*	100.0
PU-KB9-6	955	MN813130	*Streptomyces Albogriseolus*	100.0
PU-KB9-7	829	MN813131	*Streptomyces levis*	100.0
PU-KB9-8	772	MN813132	*Streptomyces paradoxus*	100.0
PU-KB9-9	825	MN813133	*Streptomyces djakartensis*	99.76
PU-KB9-10	896	MN813134	*Streptomyces collinus*	99.89
PU-KB9-11	820	MN813135	*Streptomyces paradoxus*	100.0
PU-KB9-13	910	MN813136	*Streptomyces iakyrus*	100.0
PU-KB10-2	901	MN813137	*Streptomyces griseoviridis*	99.89
PU-KB10-3	835	MN813138	*Streptomyces griseochromogenes*	99.76
PU_KB10-4	889	MN813139	*Streptomyces griseoviridis*	100.0
PU-KB10-5	899	MN813140	*Streptomyces rochei*	99.89
PU-KB10-6	892	MN813141	*Streptomyces luteus*	100.0
PU-KB10-7	872	MN813142	*Streptomyces alboflavus*	98.97
PU-KB10-8	830	MN813143	*Streptomyces atrovirens*	99.76
PU-KB10-10	791	MN813144	*Streptomyces luteogriseus*	99.87
PU-KB10-11	833	MN813145	*Streptomyces griseoviridis*	100.0
PU-KB11-2	885	MN813146	*Streptomyces griseochromogenes*	99.32
PU-KB11-3	810	MN813147	*Streptomyces nigra*	100.0
PU-KB11-4	816	MN813148	*Streptomyces nogalater*	99.88
PU-KB11-5	862	MN813149	*Streptomyces djakartensis*	99.19
PU-KB12-1	841	MN813150	*Streptomyces olivaceoviridis*	99.52
PU-KB12-3	802	MN813151	*Streptomyces wuyuanensis*	100.0
PU-KB12-4	923	MN813152	*Streptomyces nogalater*	99.89
PU-KB12-5	764	MN813153	*Streptomyces ambofaciens*	99.87
PU-KB12-7	797	MN813154	*Streptomyces olivaceoviridis*	99.50
PU-KB12-8	813	MN813155	*Streptomyces paradoxus*	100.0
PU-KB12-10	816	MN813156	*Streptomyces wuyuanensis*	100.0
PU-KB12-12	809	MN813157	*Streptomyces europaeiscabiei*	100.0
PU-KB12-14	735	MN813158	*Streptomyces paradoxus*	100.0
PU-KB12-15	836	MN813159	*Streptomyces para*	99.88
PU-KB12-16	893	MN813160	*Streptomyces wuyuanensis*	100.0
PU-KB12-17	794	MN813161	*Streptomyces olivaceoviridis*	99.50
