# Supplementary material for: Bioprospecting of desert actinobacteria with special emphases on griseoviridin, mitomycin C and a new bacterial metabolite producing Streptomyces sp. PU-KB10–4
Source: BMC Microbiol. 2023 Mar 15;23:69. doi: 10.1186/s12866-023-02770-8 (PMC10015687; doi:10.1186/s12866-023-02770-8)
Supplement: Supplementary file 4 — Additional file 4: Table S3. Microtiter plate assay for antimicrobial activity of all actinobacterial strains against bacteria (Gram-positive and Gram-negative) and yeast along with percentage cytotoxicity against cancer cell lines. [file 12866_2023_2770_MOESM4_ESM.pdf]

Table S3: Microtiter plate assay for antimicrobial activity of all actinobacterial strains against bacteria (Gram-positive and Gram-negative) and yeast along with percentage cytotoxicity against cancer cell lines

**Strains**	**Average values of Antibacterial assay (triplicates) at OD_{600} (%age inhibition)**						**Cytotoxicity (%)**	
	**E**	**S**	**B**	**M**	**St**	**Sa**	**A549**	**PC3**
PU-KB-1-3	23.7	45.4	79.1	0.0	0.0	65.7	0.0	0.0
PU-KB-1-5	40.8	43.8	94.6	34.8	0.0	52.7	0.0	15.6
PU-KB-2-1	32.2	43.6	94.5	90.1	90.2	57.0	0.0	0.0
PU-KB-2-2	35.4	48.8	74.5	92.3	0.0	63.7	54.0	4.5
PU-KB-2-3	31.4	38.8	95.1	87.8	89.0	64.4	70.5	68.7
PU-KB-2-4	35.0	47.2	94.2	83.6	87.0	68.0	70.3	69.3
PU-KB-3-2	30.7	45.9	93.9	78.8	82.6	68.0	69.9	68.0
PU-KB-3-3	30.4	43.8	60.6	67.7	3.0	67.4	0.0	0.0
PU-KB-5-2	25.4	41.1	93.4	73.2	90.6	64.0	0.0	0.0
PU-KB-5-4	19.0	35.2	57.9	0.0	0.0	66.7	0.0	0.0
PU-KB-5-5	18.3	41.8	67.8	0.0	0.0	64.4	0.0	0.0
PU-KB-5-7	33.5	37.5	93.8	0.0	0.0	54.0	0.0	0.0
PU-KB-5-11	54.9	36.6	93.9	53.3	0.0	55.7	0.0	40.1
PU-KB-5-12	30.1	36.6	55.1	0.0	0.0	57.0	6.2	8.2
PU-KB-5-15	25.0	40.8	57.5	0.0	0.0	60.0	0.0	3.2
PU-KB-5-17	33.8	42.6	94.2	87.3	88.6	64.0	72.3	68.0
PU-KB-5-19	23.7	47.0	94.3	26.6	0.0	65.4	98,2	0.0
PU-KB-5-22	12.5	41.4	66.6	0.0	0.0	65.0	0.0	6.6
PU-KB-6-1	50.1	55.7	59.9	48.6	0.0	59.7	0.0	33.9
PU-KB-6-2	34.1	43.8	66.0	84.2	25.3	65.0	73.5	70.1
PU-KB-6-3	24.8	58.9	93.1	56.3	0.0	64.7	0.0	3.6
PU-KB-6-5	48.1	40.7	74.9	43.1	0.0	51.7	0.0	0.0
PU-KB-6-7	60.2	52.8	94.2	78.7	88.4	52.7	71.4	73.7
PU-KB-6-9	34.8	50.2	74.4	45.8	0.0	62.7	0.0	0.0
PU-KB-6-10	32.3	48.3	74.4	83.0	29.1	63.0	57.8	70.5
PU-KB-6-13	30.4	58.3	81.1	22.1	0.0	56.4	0.0	0.0
PU-KB-7-4	42.0	35.9	944	60.3	86.9	50.7	70.9	72.4
PU-KB-7-5	38.4	38.7	70.8	0.0	0.0	51.4	3.5	35.9
PU-KB-7-6	43.6	52.8	74.6	40.8	0.0	57.4	0.0	0.0
PU-KB-7-7	39.3	53.1	94.0	82.4	85.2	57.4	74.4	72.8
PU-KB-7-8	40.2	36.7	92.1	66.8	87.9	55.0	74.8	72.8
PU-KB-8-1	50.6	39.3	94.3	92.9	87.9	49.7	0.0	3.5
PU-KB-8-2	43.0	49.7	94.4	80.2	85.0	55.0	74.3	71.6
PU-KB-9-1	31.5	40.7	93.0	91.4	46.2	57.7	1.5	0.0
PU-KB-9-2	31.7	40.4	65.5	38.4	0.0	59.7	1.5	0.0
PU-KB-9-4	29.6	33.5	68.3	0.0	0.0	59.4	9.0	12.6
PU-KB-9-5	26.6	37.7	50.7	55.0	0.0	70.7	98.0	98.4
PU-KB-9-6	30.7	40.0	64.8	45.2	0.0	59.0	2.9	0.0
PU-KB-9-7	25.0	40.6	94.2	75.5	0.0	61.7	2.2	1.6
PU-KB-9-8	19.9	39.5	69.7	27.7	14.9	59.4	9.8	14.4
PU-KB-9-9	46.5	44.7	94.2	79.8	78.3	52.0	83.1	65.3

PU-KB-9-10	45.2	38.0	55.5	4.0	0.0	50.4	0.0	3.2
PU-KB-9-11	43.2	50.9	91.2	66.1	0.0	61.7	0.0	24.3
PU-KB-9-12	47.0	36.7	92.2	42.9	21.1	53.4	0.0	11.9
PU-KB-9-13	31.8	37.0	74.3	36.7	0.0	61.3	3.3	0.0
PU-KB-9-14	39.2	44.6	751	42.8	0.0	58.2	2.2	17.1
PU-KB-10-1	32.6	37.2	59.3	23.2	0.0	61.5	28.7	52.5
PU-KB-10-2	31.7	42.0	76.3	91.4	0.0	64.5	7.6	9.4
PU-KB-10-3	26.5	38.0	62.0	0.0	0.0	62.4	0.0	0.0
PU-KB-10-4	78.6	88.4	92.2	83.2	85.9	62.2	16.2	38.9
PU-KB-10-5	21.0	39.8	72.9	94.4	40.2	58.1	3.6	8.9
PU-KB-10-6	40.3	44.1	94.2	93.8	35.4	49.1	2.8	10.4
PU-KB-10-7	49.6	49.2	94.3	94.4	92.7	69.1	0.0	23.3
PU-KB-10-8	46.4	42.2	94.2	87.0	0.0	70.0	0.0	2.6
PU-KB-10-9	38.7	45.6	94.6	41.3	13.4	63.6	96.4	95.7
PU-KB-10-10	35.4	47.6	79.8	36.0	0.0	62.7	16.9	19.3
PU-KB-10-11	47.8	69.1	91.1	60.0	84.2	62.5	11	37.6
PU-KB-11-2	25.5	45.3	93.6	40.9	0.0	62.5	0.3	0.0
PU-KB-11-3	25.5	39.2	57.4	39.0	0.0	62.5	0.6	1.2
PU-KB-11-4	18.9	43.1	71.2	47.7	0.0	67.5	0.7	0.0
PU-KB-11-5	23.1	40.5	66.0	54.9	0.0	59.4	0.0	29.3
PU-KB-12-1	36.3	41.6	94.1	0.0.1	0.0	51.8	0.0	0.0
PU-KB-12-3	29.8	38.8	94.2	88.8	90.3	50.2	0.0	27.8
PU-KB-12-7	38.4	36.4	95.7	0.0	0.0	53.5	0.0	0.0
PU-KB-12-8	47.8	37.4	93.5	77.0	0.0	55.4	8.4	67.1
PU-KB-12-12	47.7	44.7	68.8	72.5	0.0	59.8	99.3	99.0
PU-KB-12-13	37.6	46.1	72.1	45.1	0.0	64.8	0.0	2.4
PU-KB-12-15	36.8	44.7	94.3	71.5	87.9	63.0	75.7	69.6
PU-KB-12-17	16.6	41.1	94.2	31.8	0.0	57.0	0.0	0.0
Ampicillin	95.2	95.1	97.6	97.2	98.2	100.1	-	-
Kanamycin	98.8	97.8	93.2	92.4	93.6	100.0	-	-
Blank	-	-	-	-	-	-	0.0	0.0
Actinomycin D	-	-	-	-	-	-	99.8	99.6
DMSO	-	-	-	-	-	-	0.5	1.2

Key: E *Escherichia coli* (NRRL B-3708), **S** *Salmonella enterica* (ATCC 10708), **B** *Bacillus subtilis* (ATCC 6633), **M** *Micrococcus luteus* (NRRL B-287), **St** *Staphylococcus aureus* (ATCC 6538), **Sa** *Saccharomyces cerevisiae* (ATCC 204508).
