# Supplementary material for: Bioprospecting of desert actinobacteria with special emphases on griseoviridin, mitomycin C and a new bacterial metabolite producing Streptomyces sp. PU-KB10–4
Source: BMC Microbiol. 2023 Mar 15;23:69. doi: 10.1186/s12866-023-02770-8 (PMC10015687; doi:10.1186/s12866-023-02770-8)
Supplement: Supplementary file 5 — Additional file 5: Fig. S2. TLC (CH2Cl2/10%MeOH) screening of the extracts produced PU-KB strains. [file 12866_2023_2770_MOESM5_ESM.pdf]

TLC analysis of PU-KB bacterial extracts

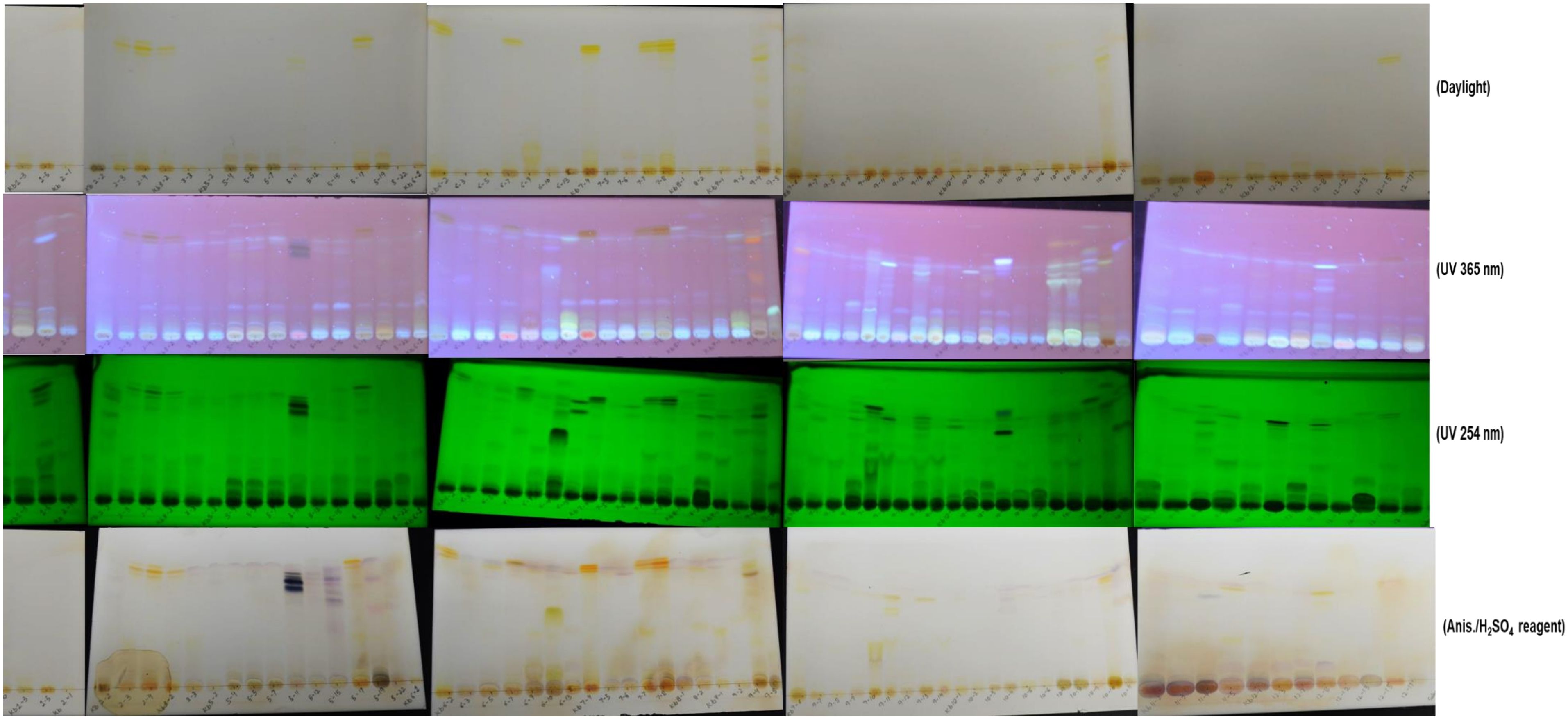

Figure S2. TLC (CH_2Cl_2/10%MeOH) screening of the extracts produced PU-KB strains.
