# Supplementary material for: Bioprospecting of desert actinobacteria with special emphases on griseoviridin, mitomycin C and a new bacterial metabolite producing Streptomyces sp. PU-KB10–4
Source: BMC Microbiol. 2023 Mar 15;23:69. doi: 10.1186/s12866-023-02770-8 (PMC10015687; doi:10.1186/s12866-023-02770-8)
Supplement: Supplementary file 7 — Additional file 7: Fig. S4. HPLC/UV analyses of the generated extract produced by Streptomyces sp. PU-KB10-4 (A-medium). HPLC-conditions: Detection wavelength 320 and 280 nm;; solvent A: H2O/0.1% TFA; solvent B: acetonitrile; flow rate: 1.0 mL min-1; 0-30 min, 95-0% A (linear gradient); 30-35 min 0% A; 35-36 min 0-95% A (linear gradient); 36-40 min 95% A. UV-vis inset of full wavelength scan (190-600 nm). [file 12866_2023_2770_MOESM7_ESM.pdf]

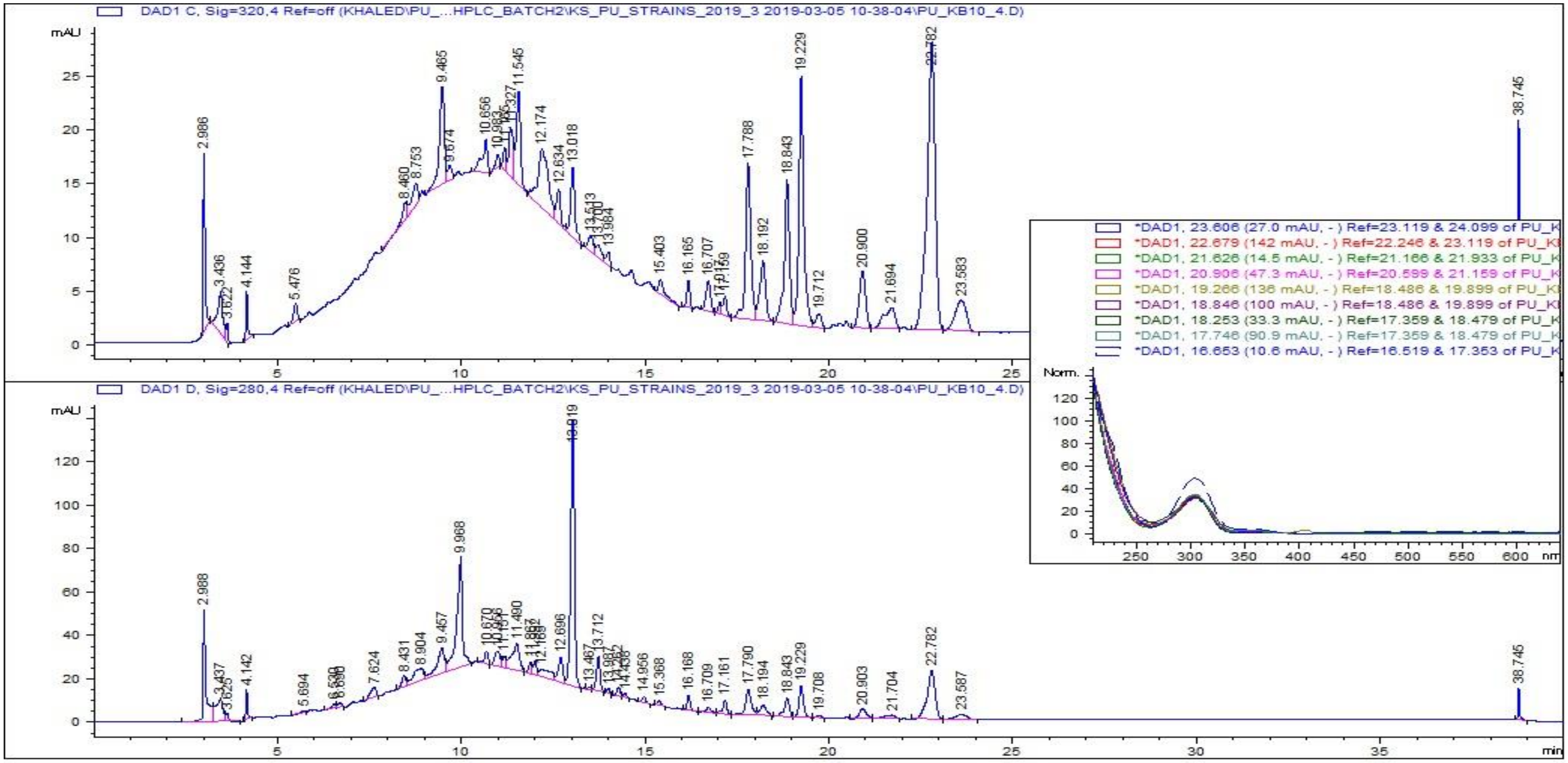

Figure S4: HPLC/UV analyses of the generated extract produced by *Streptomyces* sp. PU-KB10-4 (A-medium). HPLC-conditions: Detection wavelength 320 and 280 nm; solvent A: H_2O/0.1% TFA; solvent B: acetonitrile; flow rate: 1.0 mL min^{-1}; 0-30 min, 95-0% A (linear gradient); 30-35 min 0% A; 35-36 min 0-95% A (linear gradient); 36-40 min 95% A. UV-vis inset of full wavelength scan (190-600 nm).
