# Supplementary figures and images for: Bioprospecting of desert actinobacteria with special emphases on griseoviridin, mitomycin C and a new bacterial metabolite producing Streptomyces sp. PU-KB10–4
Source: BMC Microbiol. 2023 Mar 15;23:69. doi: 10.1186/s12866-023-02770-8 (PMC10015687; doi:10.1186/s12866-023-02770-8)

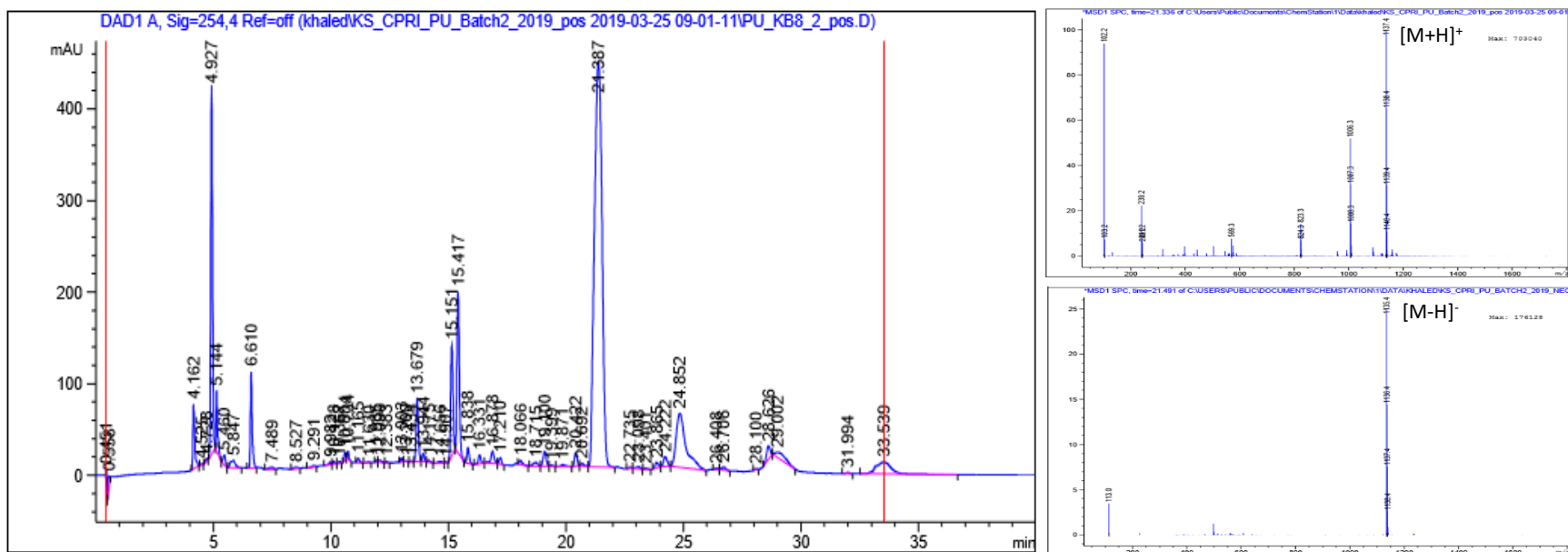

Supplement: Supplementary file 8 — Additional file 8: Fig. S5. HPLC-MS analysis of PU-KB8-2 crude extract. HPLC-conditions: solvent A: H2O/0.1% FA; solvent B: CH3CN; flow rate: 0.5 mL min-1; 0-30 min, 5-100% B; 30-35 min, 100% B; 35-36 min, 100-5% B; 36-40 min, 5% B; Phenomenex NX-C18 column (250 × 4.6 mm, 5 μm); 254 nm. [file 12866_2023_2770_MOESM8_ESM.pdf]

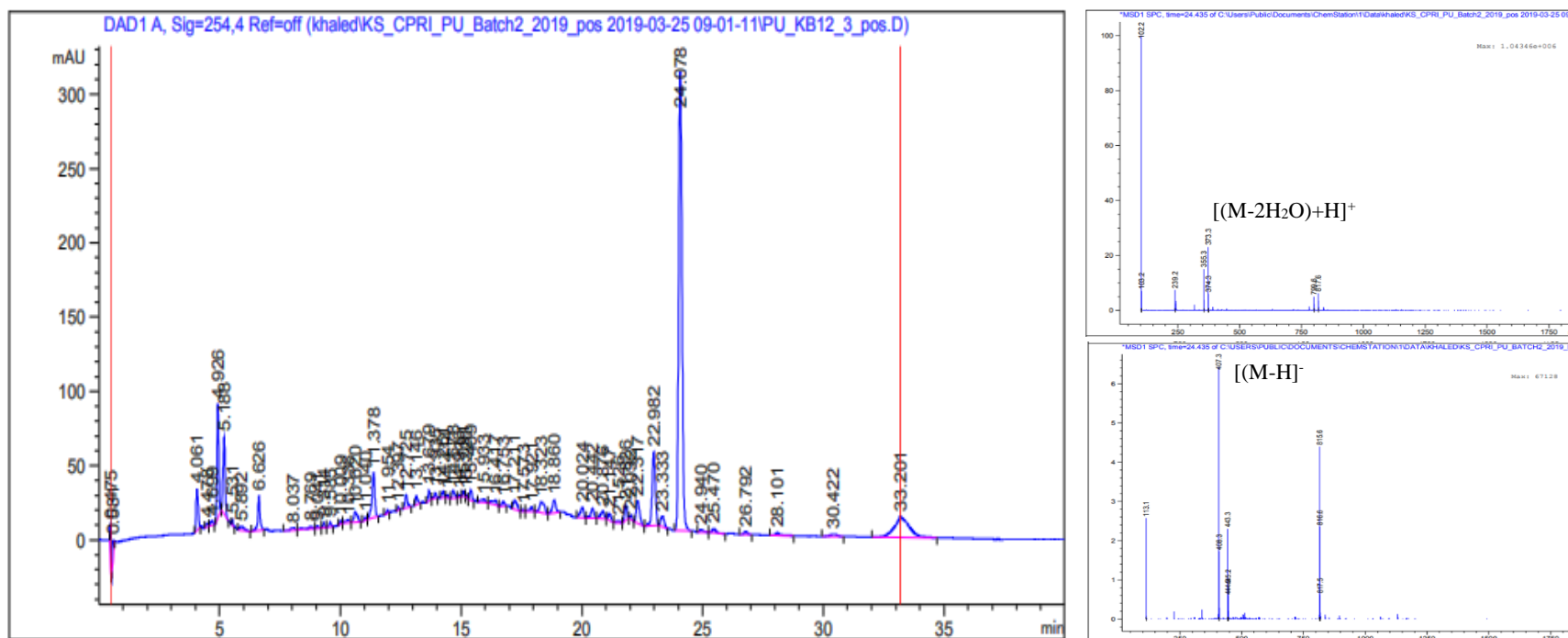

Supplement: Supplementary file 11 — Additional file 11: Fig. S8. HPLC-MS analysis of PU-KB12-3 crude extract. HPLC-conditions: solvent A: H2O/0.1% FA; solvent B: CH3CN; flow rate: 0.5 mL min-1; 0-30 min, 5-100% B; 30-35 min, 100% B; 35-36 min, 100-5% B; 36-40 min, 5% B; Phenomenex NX-C18 column (250 × 4.6 mm, 5 μm); 254 nm. [file 12866_2023_2770_MOESM11_ESM.pdf]

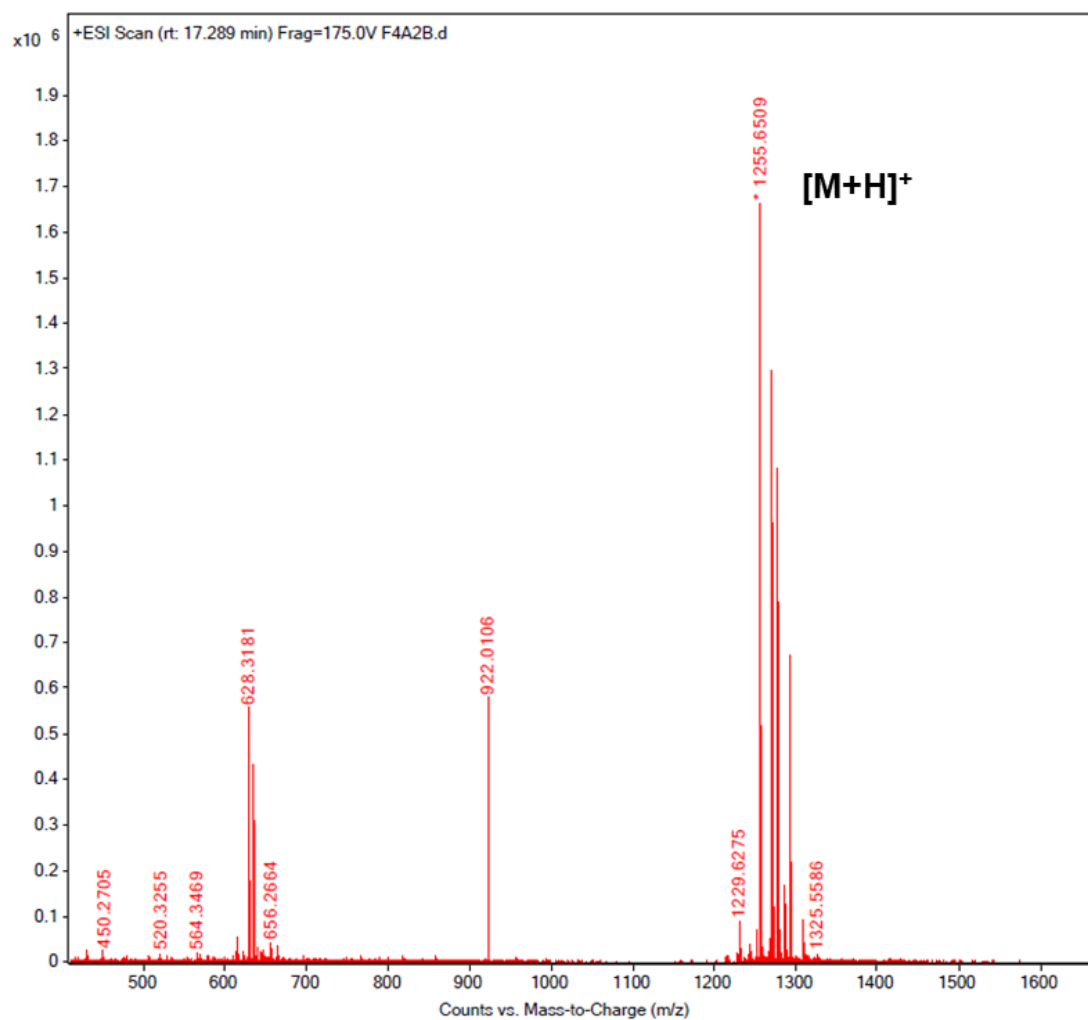

**Figure S12.** (+)-HRMS of analysis of actinomycin D.

Supplement: Supplementary file 15 — Additional file 15: Fig. S12. (+)-HRMS of analysis of actinomycin D. [file 12866_2023_2770_MOESM15_ESM.pdf]

## 1D and 2D NMR spectrum of griseoviridin (1)

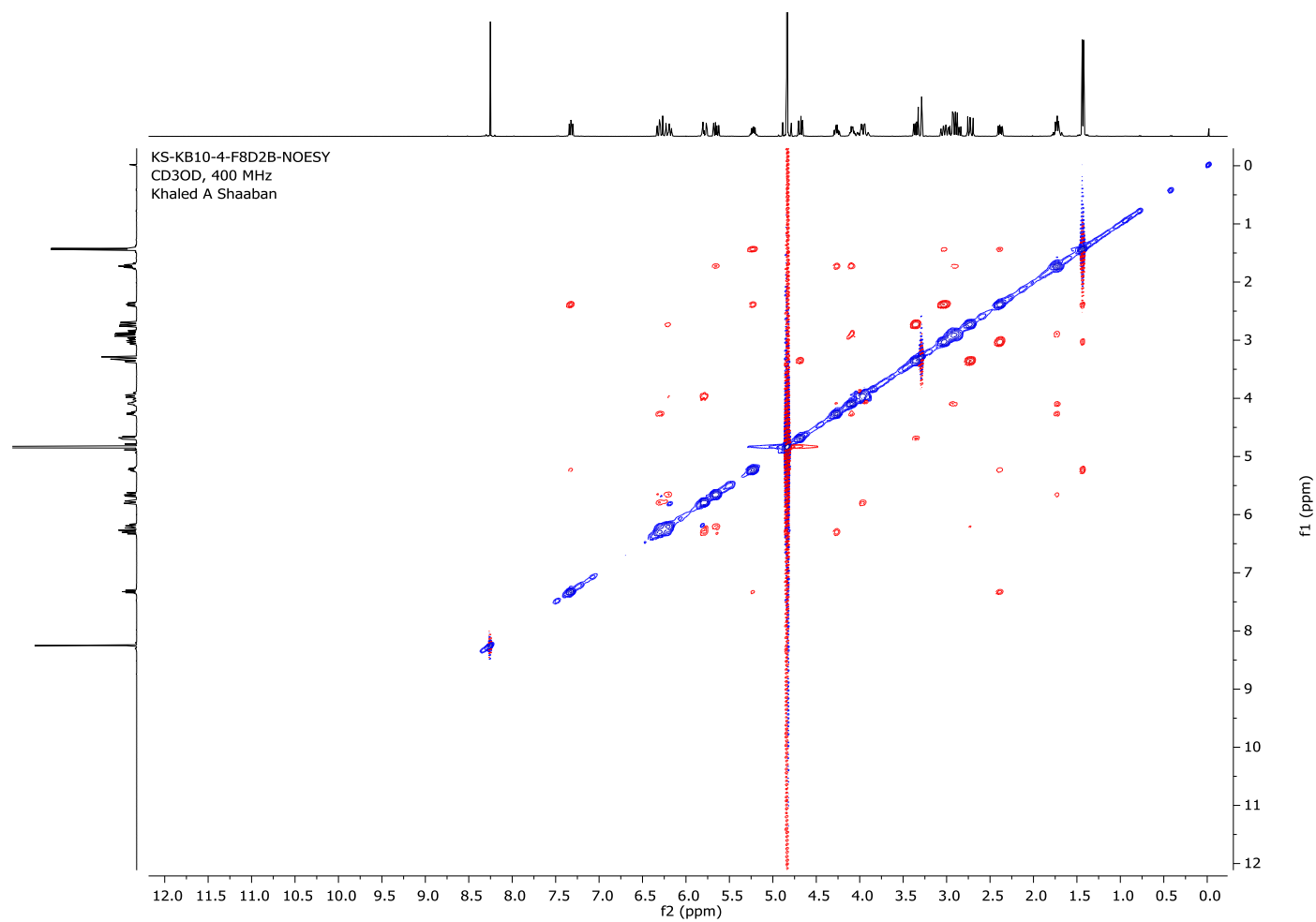

**Figure S18:** NOESY spectrum (CD<sub>3</sub>OD, 400 MHz) of griseoviridin (**1**).

Supplement: Supplementary file 21 — Additional file 21: Fig. S18. NOESY spectrum (CD3OD, 400 MHz) of griseoviridin (1). [file 12866_2023_2770_MOESM21_ESM.pdf]

## 1D and 2D NMR spectrum of griseoviridin (1)

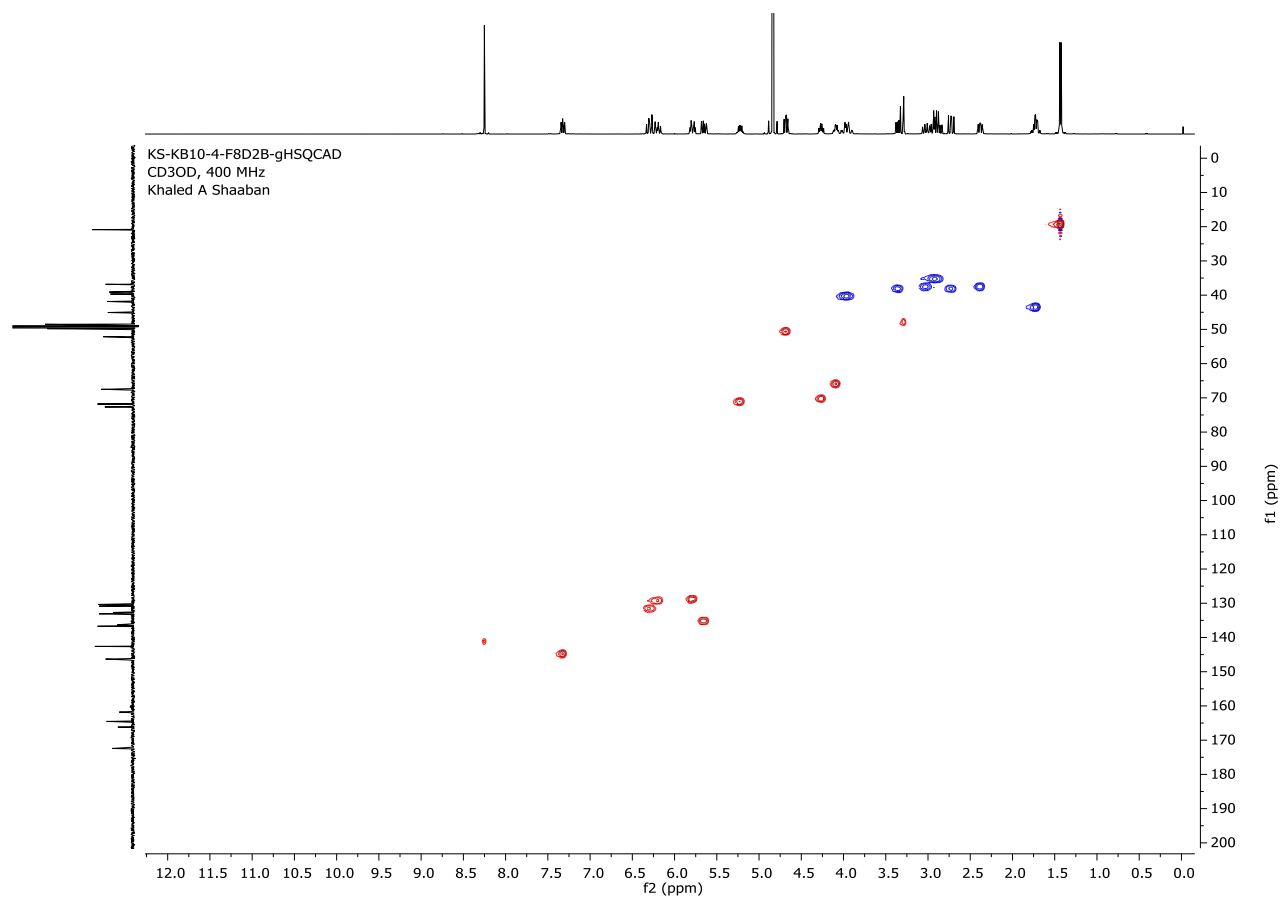

**Figure S20:** HSQC spectrum (CD<sub>3</sub>OD, 400 MHz) of griseoviridin (1).

Supplement: Supplementary file 23 — Additional file 23: Fig. S20. HSQC spectrum (CD3OD, 400 MHz) of griseoviridin (1). [file 12866_2023_2770_MOESM23_ESM.pdf]

## 1D and 2D NMR spectrum of griseoviridin (1)

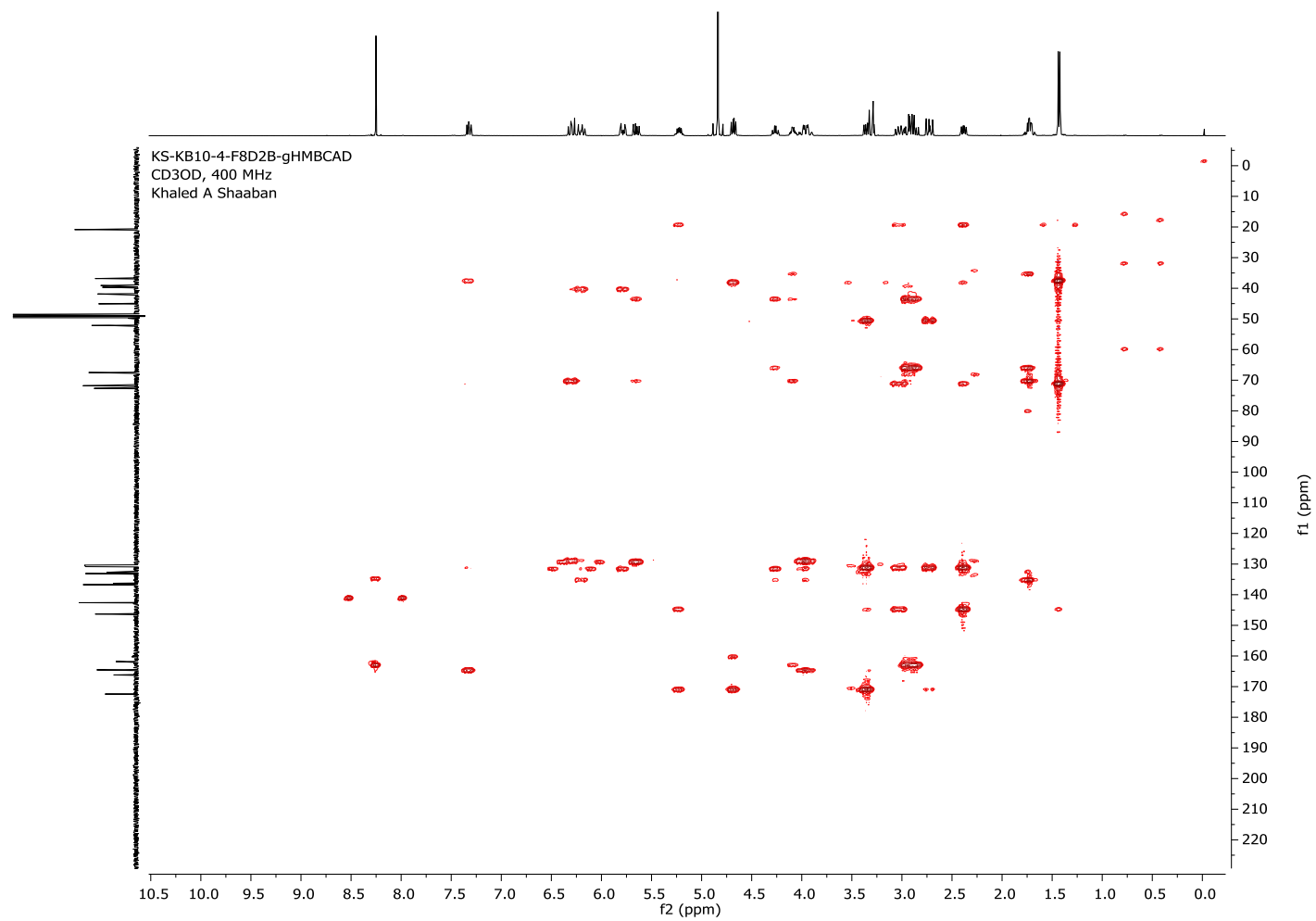

**Figure S21:** HMBC spectrum (CD<sub>3</sub>OD, 400 MHz) of griseoviridin (1).

Supplement: Supplementary file 24 — Additional file 24: Fig. S21. HMBC spectrum (CD3OD, 400 MHz) of griseoviridin (1). [file 12866_2023_2770_MOESM24_ESM.pdf]

## 1D and 2D NMR spectrum of griseoviridin (1)

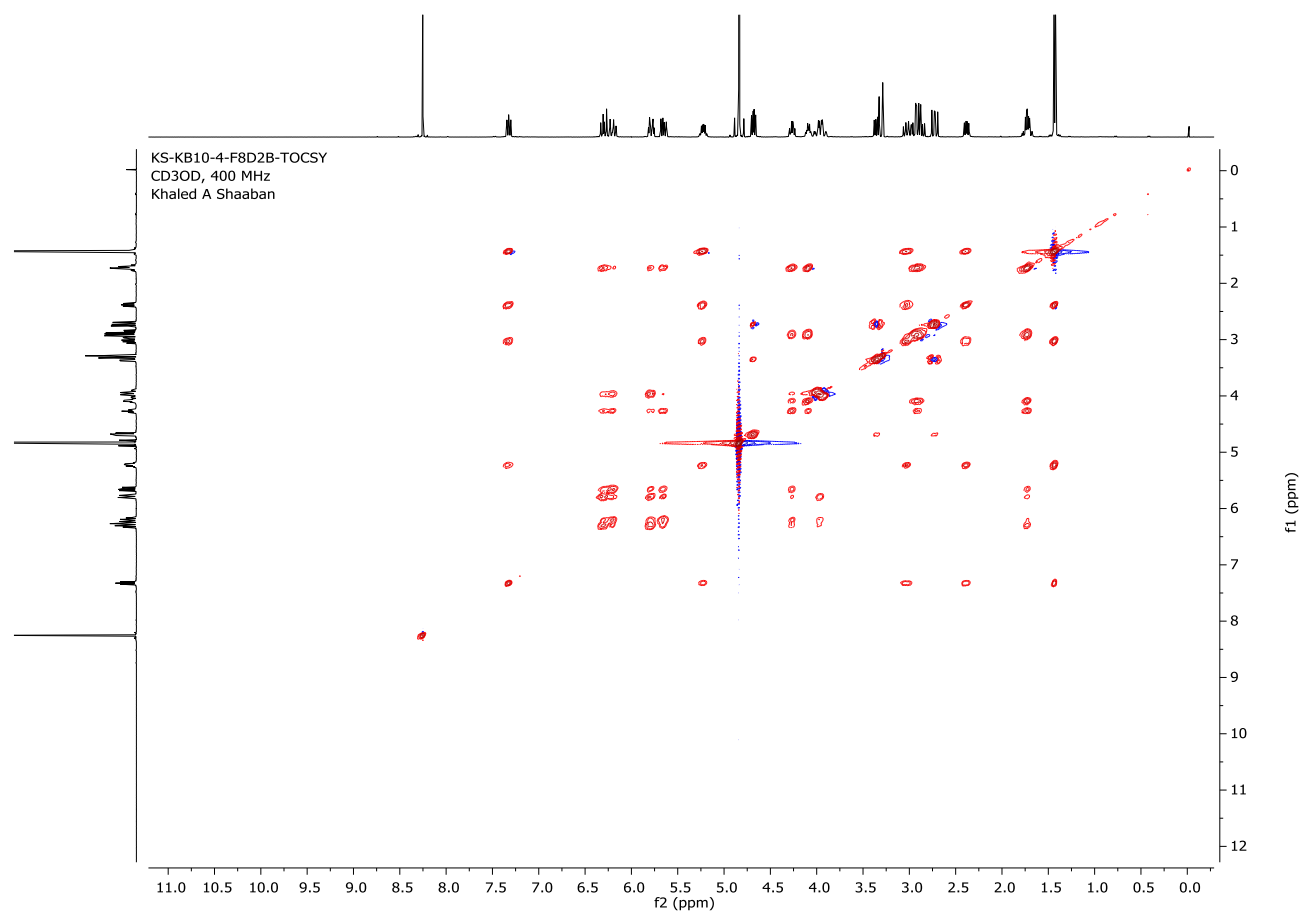

**Figure S22:** TOCSY spectrum (CD<sub>3</sub>OD, 400 MHz) of griseoviridin (**1**).

Supplement: Supplementary file 25 — Additional file 25: Fig. S22. TOCSY spectrum (CD3OD, 400 MHz) of griseoviridin (1). [file 12866_2023_2770_MOESM25_ESM.pdf]

## 1D and 2D NMR spectrum of mitomycin C (2)

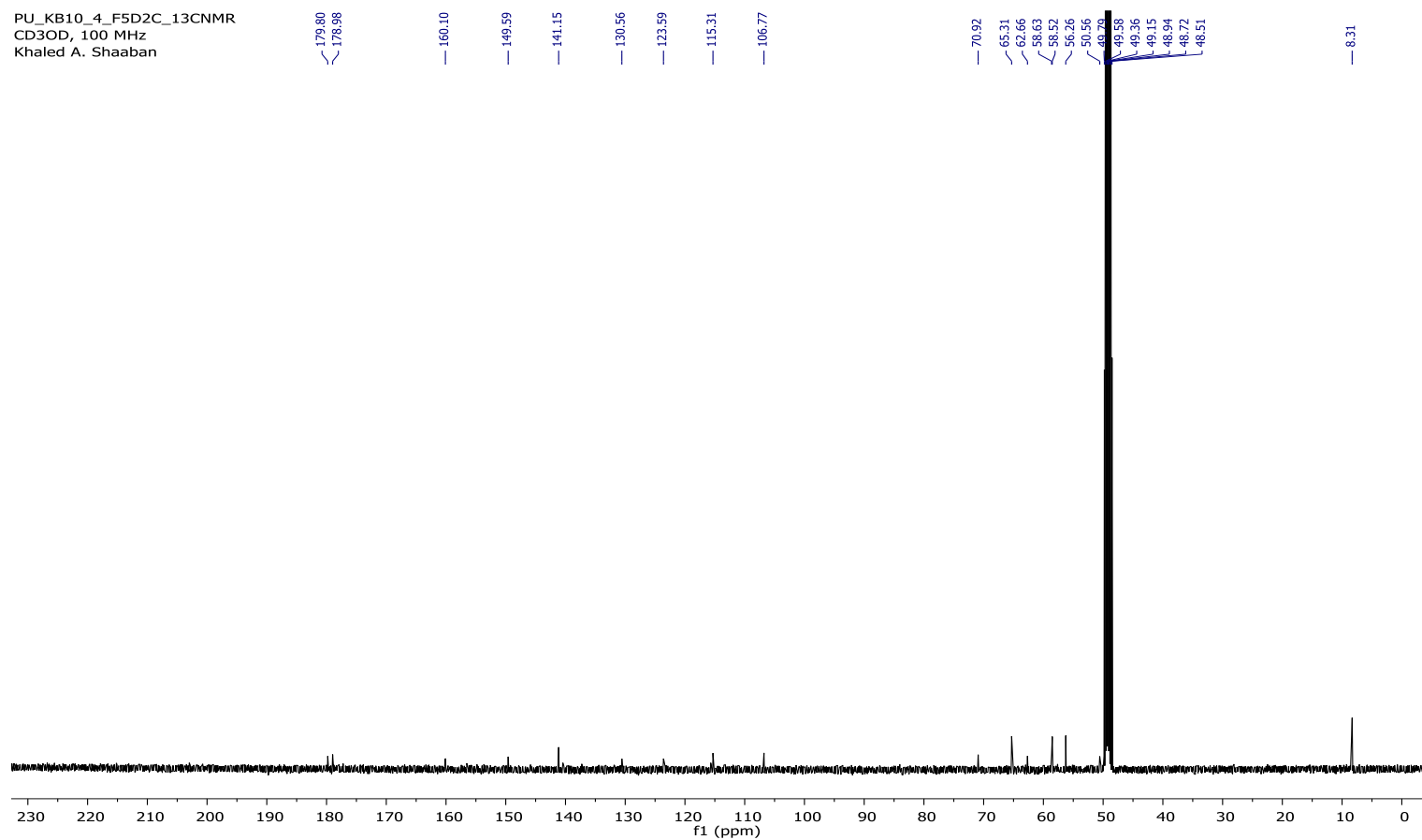

**Figure S25:**  $^{13}\text{C}$  NMR spectrum ( $\text{CD}_3\text{OD}$ , 100 MHz) of mitomycin C (2).

Supplement: Supplementary file 28 — Additional file 28: Fig. S25.13C NMR spectrum (CD3OD, 100 MHz) of mitomycin C (2). [file 12866_2023_2770_MOESM28_ESM.pdf]

### 1D and 2D NMR spectrum of 4-hydroxycinnamide (3)

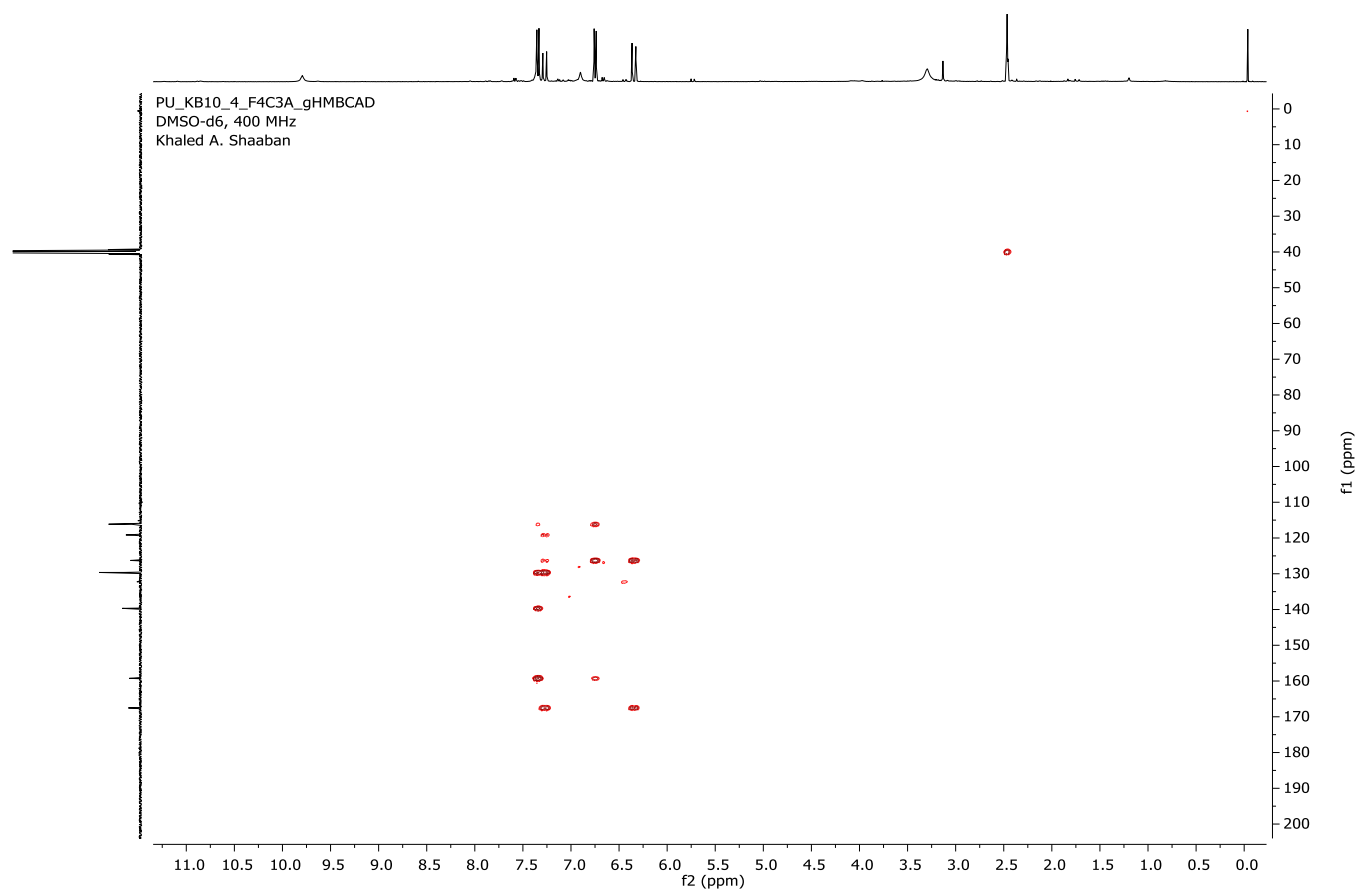

**Figure S37:** HMBC spectrum (DMSO- $d_6$ , 400 MHz) of 4-hydroxycinnamide (**3**).

Supplement: Supplementary file 40 — Additional file 40: Fig. S37. HMBC spectrum (DMSO-d6, 400 MHz) of 4-hydroxycinnamide (3). [file 12866_2023_2770_MOESM40_ESM.pdf]
