# Supplementary material for: Bioprospecting of desert actinobacteria with special emphases on griseoviridin, mitomycin C and a new bacterial metabolite producing Streptomyces sp. PU-KB10–4
Source: BMC Microbiol. 2023 Mar 15;23:69. doi: 10.1186/s12866-023-02770-8 (PMC10015687; doi:10.1186/s12866-023-02770-8)
Supplement: Supplementary file 12 — Additional file 12: Figs. S9. Fermentation cultures of actinomycin D producing strains (A-medium, 10d, 210 rpm, 28 °C). The dark brown culture of strain PU-KB6-7 is due to high concentration of metal-complex siderophores (Fe – complex), which was consistent with the detected peak in HPLC/UV analysis figure at 18.8 min, and the low concentration of actinomycin D at 25.3 min. [file 12866_2023_2770_MOESM12_ESM.pdf]

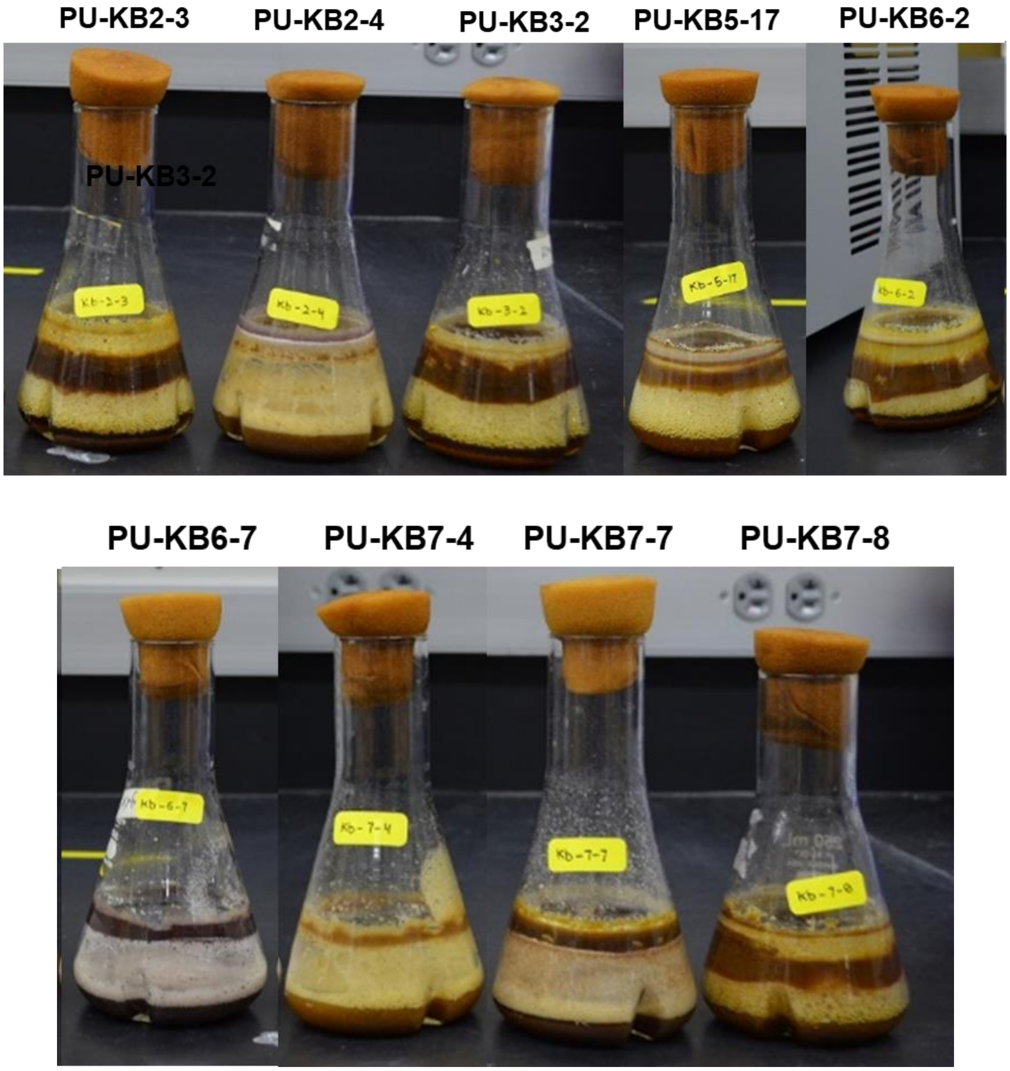

Figures S9. Fermentation cultures of actinomycin D producing strains (A-medium, 10d, 210 rpm, 28 ºC). The dark brown culture of strain PU-KB6-7 is due to high concentration of metal-complex siderophores (Fe – complex), which was consistent with the detected peak in HPLC/UV analysis figure at 18.8 min, and the low concentration of actinomycin D at 25.3 min.
