# Supplementary material for: Bioprospecting of desert actinobacteria with special emphases on griseoviridin, mitomycin C and a new bacterial metabolite producing Streptomyces sp. PU-KB10–4
Source: BMC Microbiol. 2023 Mar 15;23:69. doi: 10.1186/s12866-023-02770-8 (PMC10015687; doi:10.1186/s12866-023-02770-8)
Supplement: Supplementary file 13 — Additional file 13: Fig. S10. HPLC/UV analyses of the generated extract produced by actinomycin D producing strains (A-medium). [file 12866_2023_2770_MOESM13_ESM.pdf]

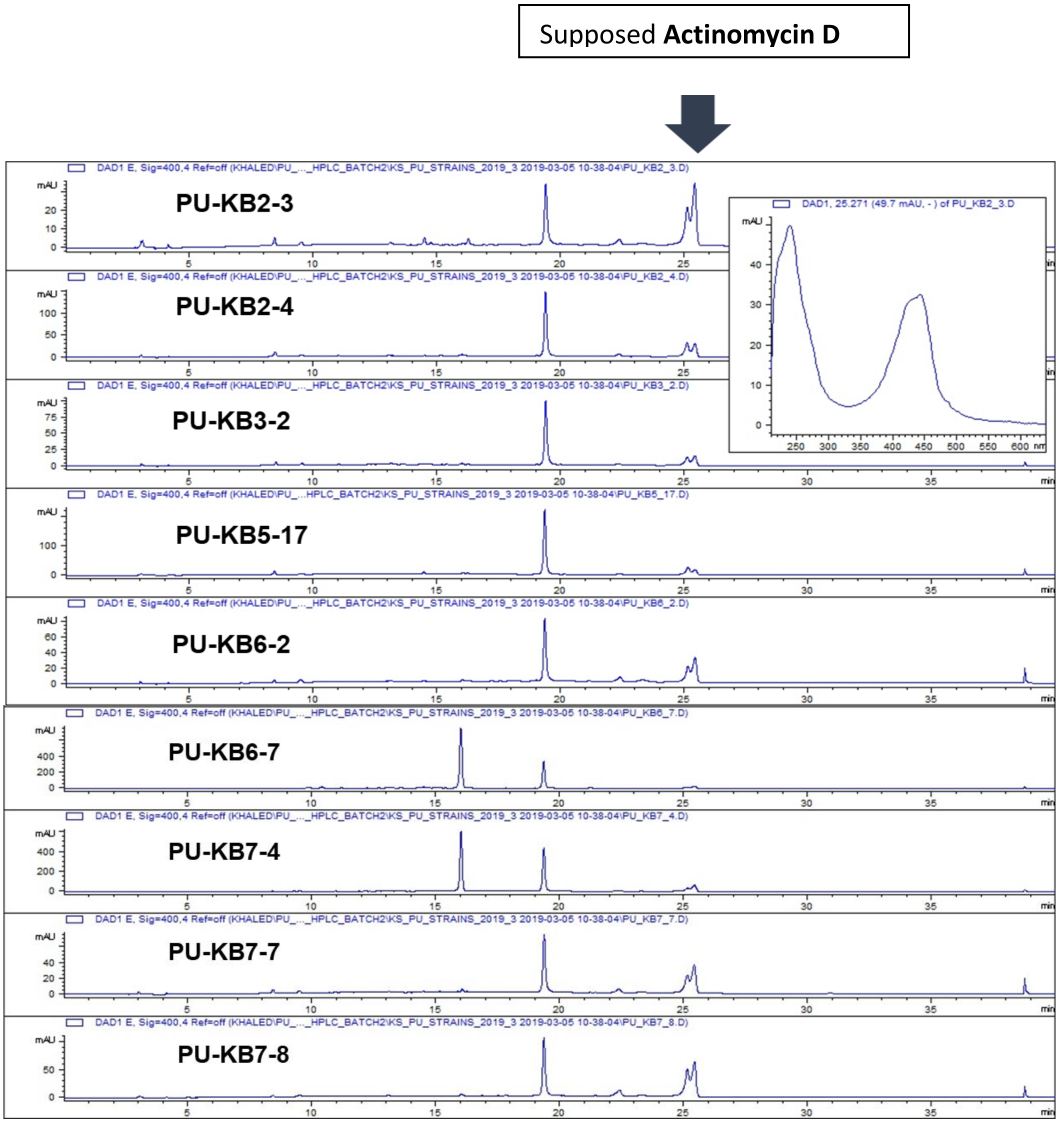

Figure S10: HPLC/UV analyses of the generated extract produced by actinomycin D producing strains (A-medium).
