# Supplementary material for: Bioprospecting of desert actinobacteria with special emphases on griseoviridin, mitomycin C and a new bacterial metabolite producing Streptomyces sp. PU-KB10–4
Source: BMC Microbiol. 2023 Mar 15;23:69. doi: 10.1186/s12866-023-02770-8 (PMC10015687; doi:10.1186/s12866-023-02770-8)
Supplement: Supplementary file 14 — Additional file 14: Fig. S11. HPLC-MS analysis of one selected actinomycin D producer crude extract (PU-KB7-8). [file 12866_2023_2770_MOESM14_ESM.pdf]

Actinomycin D
$C_{62}H_{86}N_{12}O_{16}$ (1254)

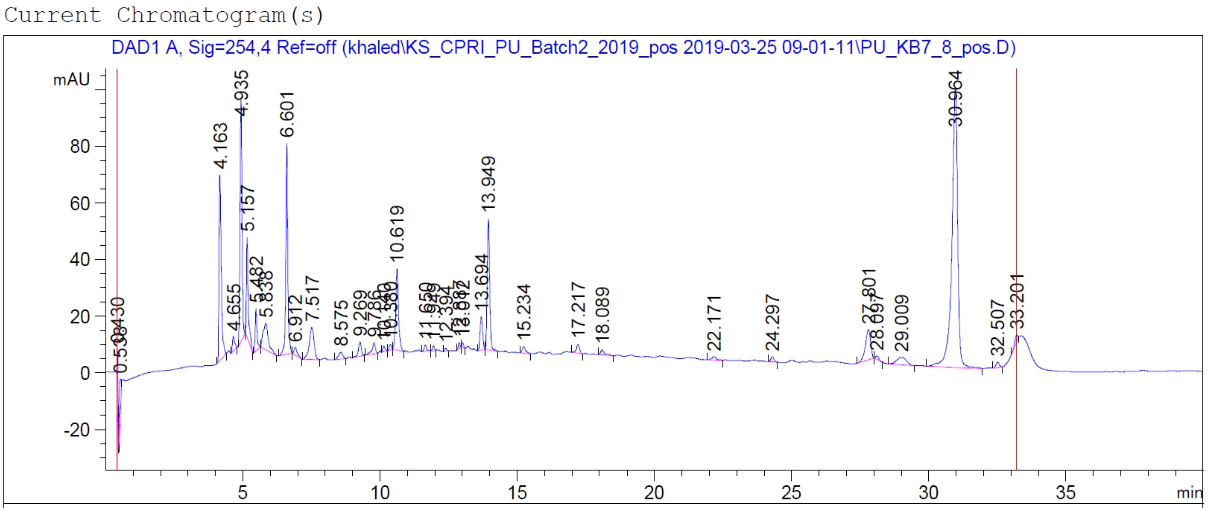

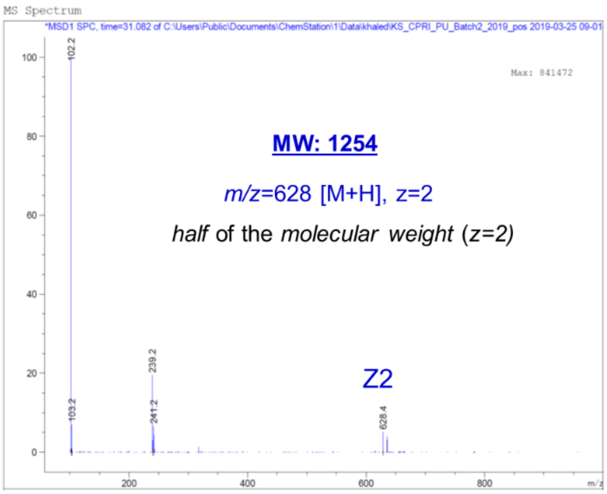

Figure S11: HPLC-MS analysis of one selected actinomycin D (**4**) producer crude extract (PU-KB7-8).
