# Supplementary material for: Bioprospecting of desert actinobacteria with special emphases on griseoviridin, mitomycin C and a new bacterial metabolite producing Streptomyces sp. PU-KB10–4
Source: BMC Microbiol. 2023 Mar 15;23:69. doi: 10.1186/s12866-023-02770-8 (PMC10015687; doi:10.1186/s12866-023-02770-8)
Supplement: Supplementary file 20 — Additional file 20: Fig. S17. 1H (400 MHz) and 13C (100 MHz) NMR spectra of griseoviridin (1) in CD3OD. [file 12866_2023_2770_MOESM20_ESM.pdf]

1D and 2D NMR spectrum of griseoviridin (1)

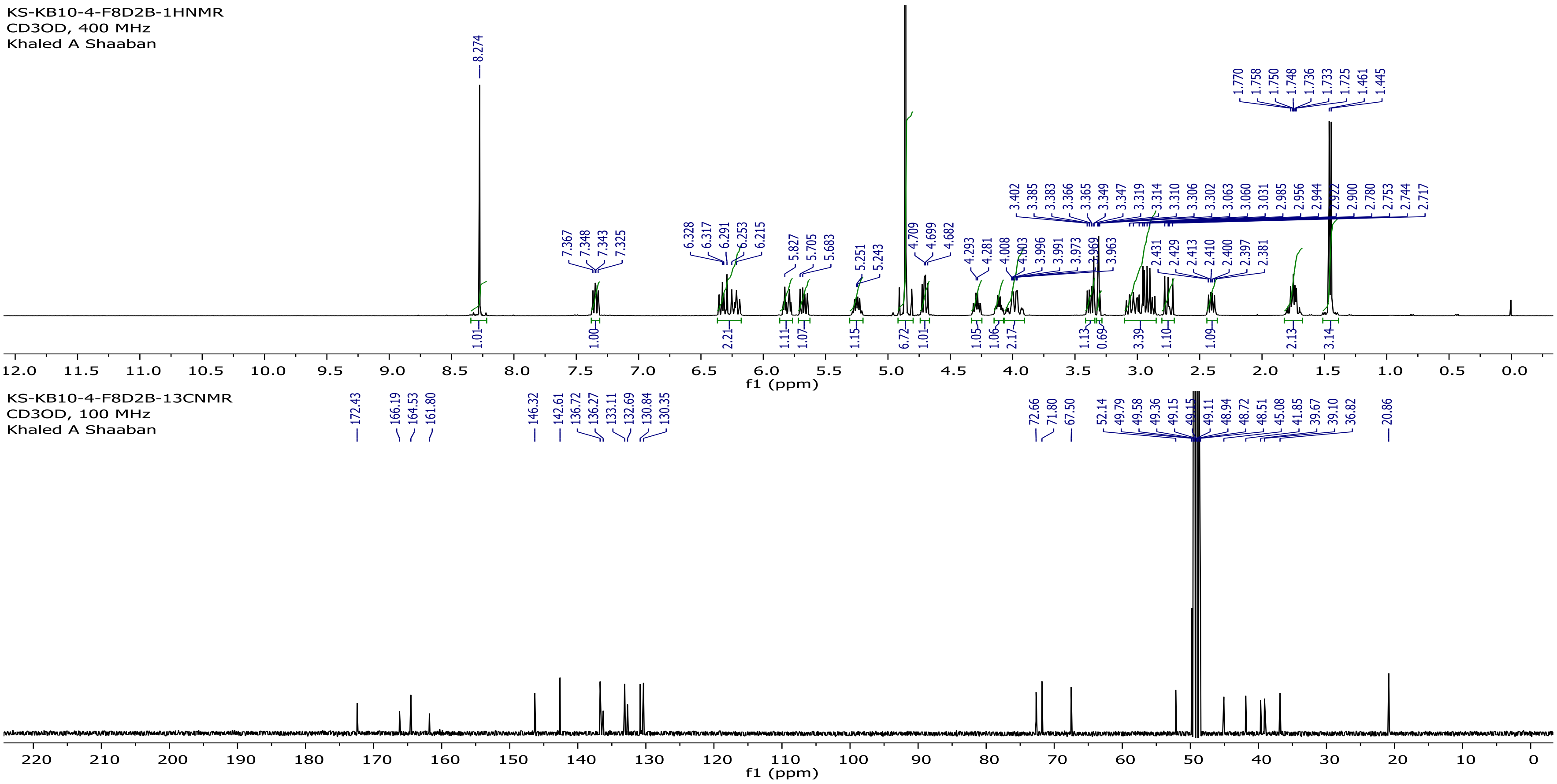

Figure S17: ^{1}H (400 MHz) and ^{13}C (100 MHz) NMR spectra of griseoviridin (**1**) in CD_3OD.
