# Supplementary material for: Bioprospecting of desert actinobacteria with special emphases on griseoviridin, mitomycin C and a new bacterial metabolite producing Streptomyces sp. PU-KB10–4
Source: BMC Microbiol. 2023 Mar 15;23:69. doi: 10.1186/s12866-023-02770-8 (PMC10015687; doi:10.1186/s12866-023-02770-8)
Supplement: Supplementary file 22 — Additional file 22: Fig. S19. 1H,1H-COSY spectrum (CD3OD, 400 MHz) of griseoviridin (1). [file 12866_2023_2770_MOESM22_ESM.pdf]

1D and 2D NMR spectrum of griseoviridin (1)

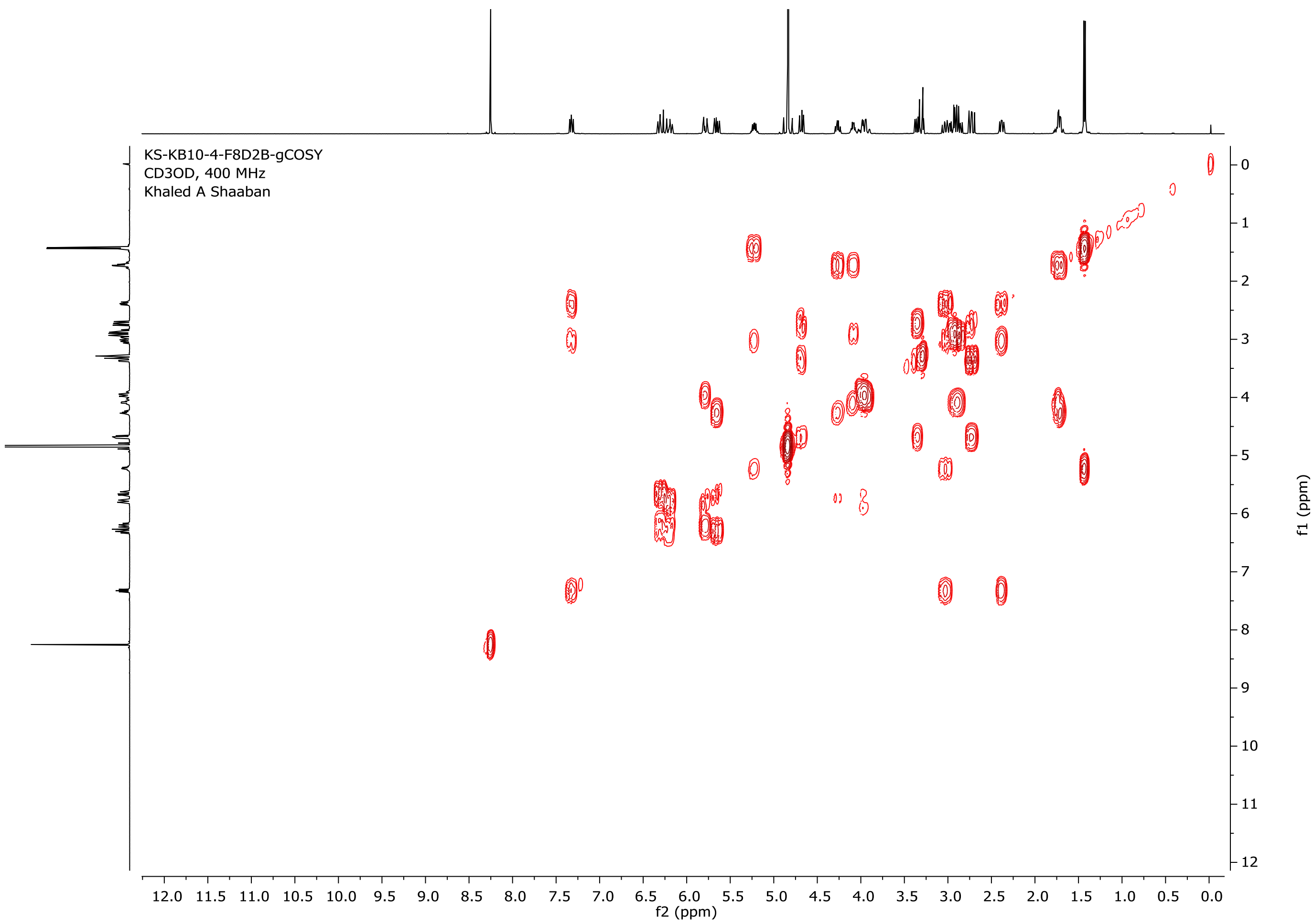

Figure S19: $^{1}H,^{1}H$-COSY spectrum (CD_3OD, 400 MHz) of griseoviridin (**1**).
