# Supplementary material for: Bioprospecting of desert actinobacteria with special emphases on griseoviridin, mitomycin C and a new bacterial metabolite producing Streptomyces sp. PU-KB10–4
Source: BMC Microbiol. 2023 Mar 15;23:69. doi: 10.1186/s12866-023-02770-8 (PMC10015687; doi:10.1186/s12866-023-02770-8)
Supplement: Supplementary file 29 — Additional file 29: Fig. S26. 1H,1H-COSY spectrum (CD3OD, 500 MHz) of mitomycin C (2). [file 12866_2023_2770_MOESM29_ESM.pdf]

# 1D and 2D NMR spectrum of mitomycin C (2)

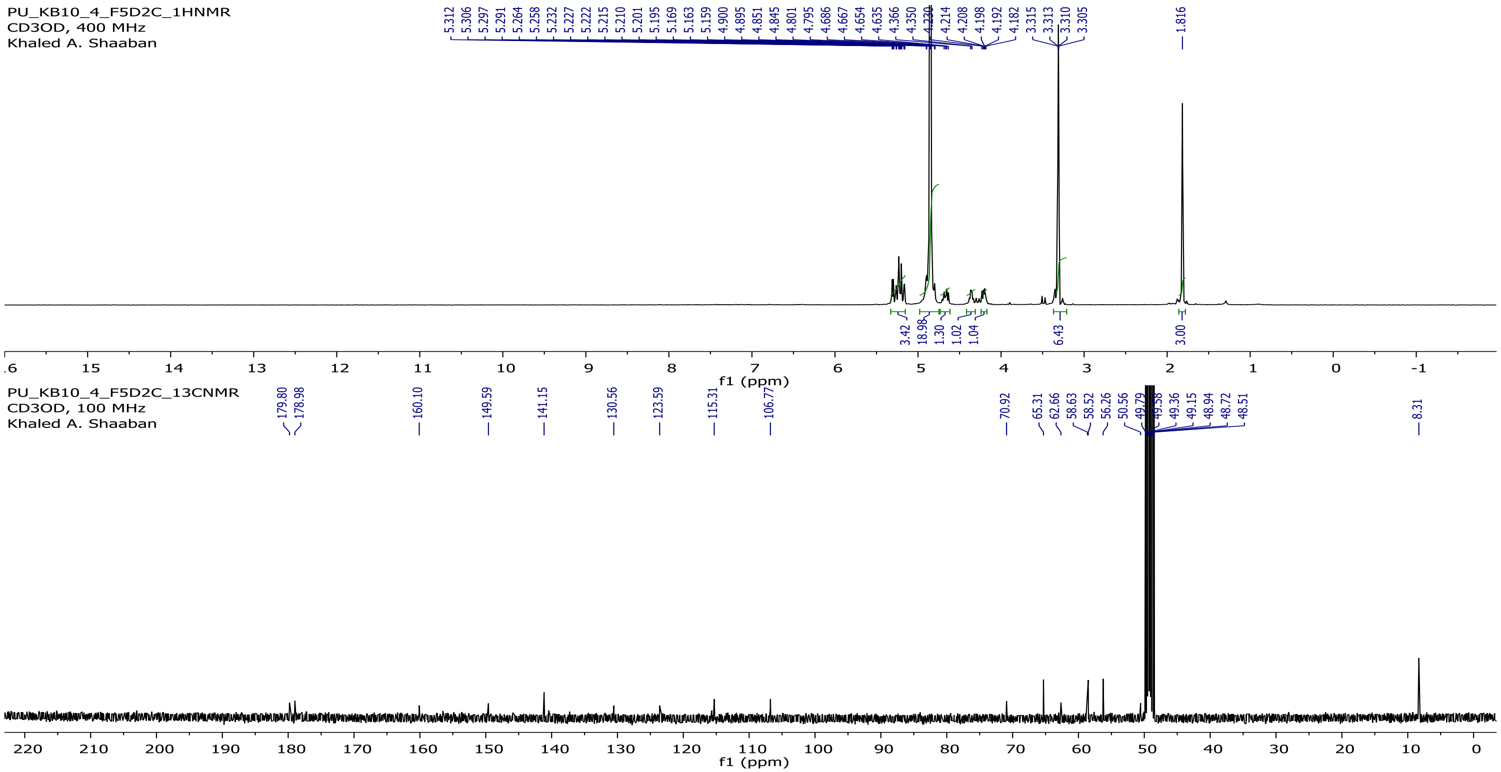


**Figure S26:** $^{1}H,^{1}H$-COSY spectrum ($CD_3OD$, 500 MHz) of mitomycin C (**2**).
