# Supplementary material for: Bioprospecting of desert actinobacteria with special emphases on griseoviridin, mitomycin C and a new bacterial metabolite producing Streptomyces sp. PU-KB10–4
Source: BMC Microbiol. 2023 Mar 15;23:69. doi: 10.1186/s12866-023-02770-8 (PMC10015687; doi:10.1186/s12866-023-02770-8)
Supplement: Supplementary file 30 — Additional file 30: Fig. S27. 1H (400 MHz) and 13C (100 MHz) NMR spectra of mitomycin C (2) in CD3OD. [file 12866_2023_2770_MOESM30_ESM.pdf]

1D and 2D NMR spectrum of mitomycin C (2)

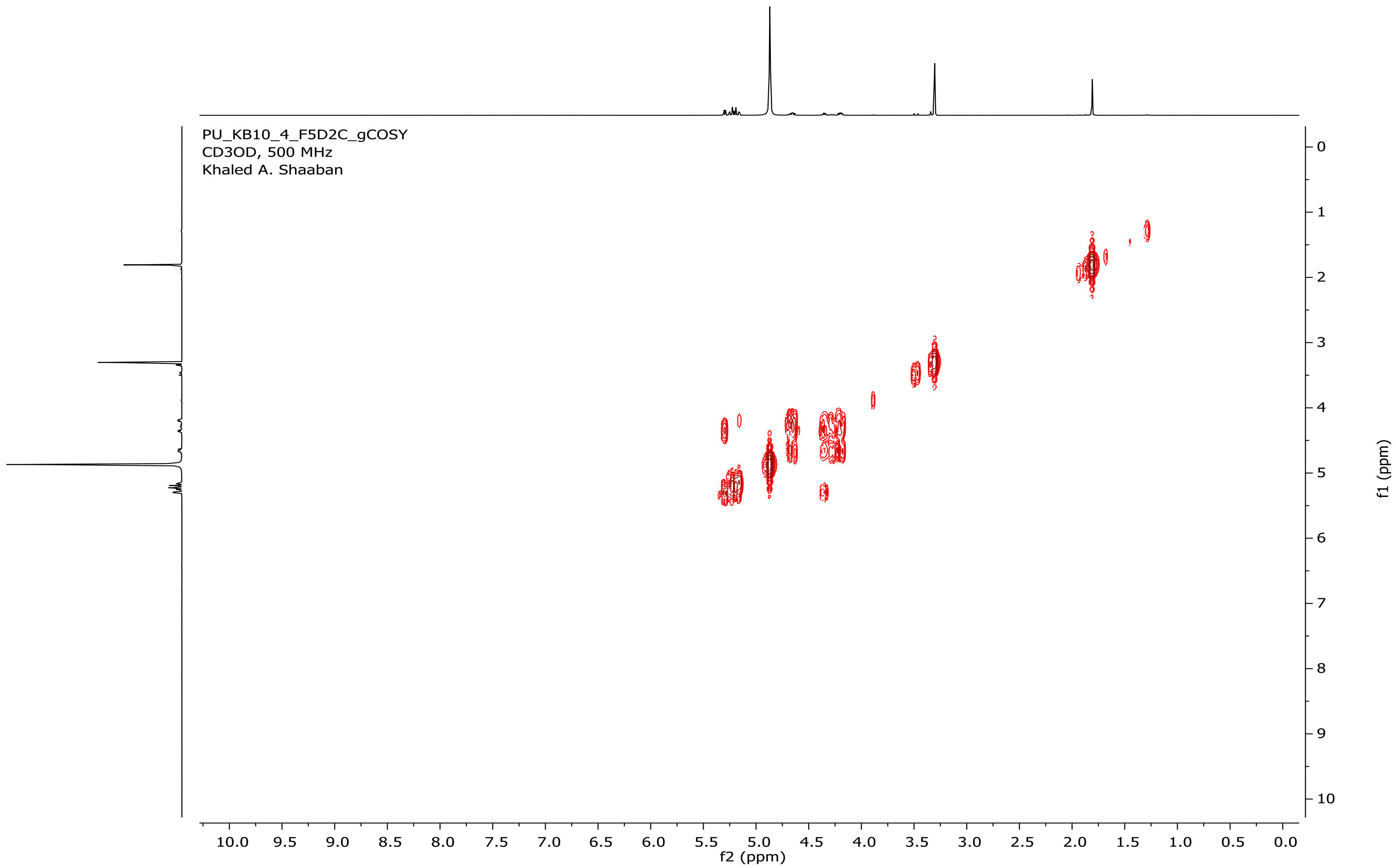

Figure S27: ^{1}H (400 MHz) and ^{13}C (100 MHz) NMR spectra of mitomycin C (**2**) in CD_3OD.
