# Supplementary material for: Bioprospecting of desert actinobacteria with special emphases on griseoviridin, mitomycin C and a new bacterial metabolite producing Streptomyces sp. PU-KB10–4
Source: BMC Microbiol. 2023 Mar 15;23:69. doi: 10.1186/s12866-023-02770-8 (PMC10015687; doi:10.1186/s12866-023-02770-8)
Supplement: Supplementary file 31 — Additional file 31 Fig. S28. HPLC analysis of 4-hydroxycinnamide (3). HPLC-conditions: solvent A: H2O/0.1% FA; solvent B: CH3CN; flow rate: 0.5 mL min-1; 0-30 min, 5-100% B; 30-35 min, 100% B; 35-36 min, 100-5% B; 36-40 min, 5% B; Phenomenex NX-C18 column (250 × 4.6 mm, 5 μm); 254 nm. UV-vis inset of full wavelength scan (190-600 nm). (+) and (–)-ESI-MS spectra of 4-hydroxycinnamide (3). [file 12866_2023_2770_MOESM31_ESM.pdf]

HPLC of 4-hydroxycinnamide (3)

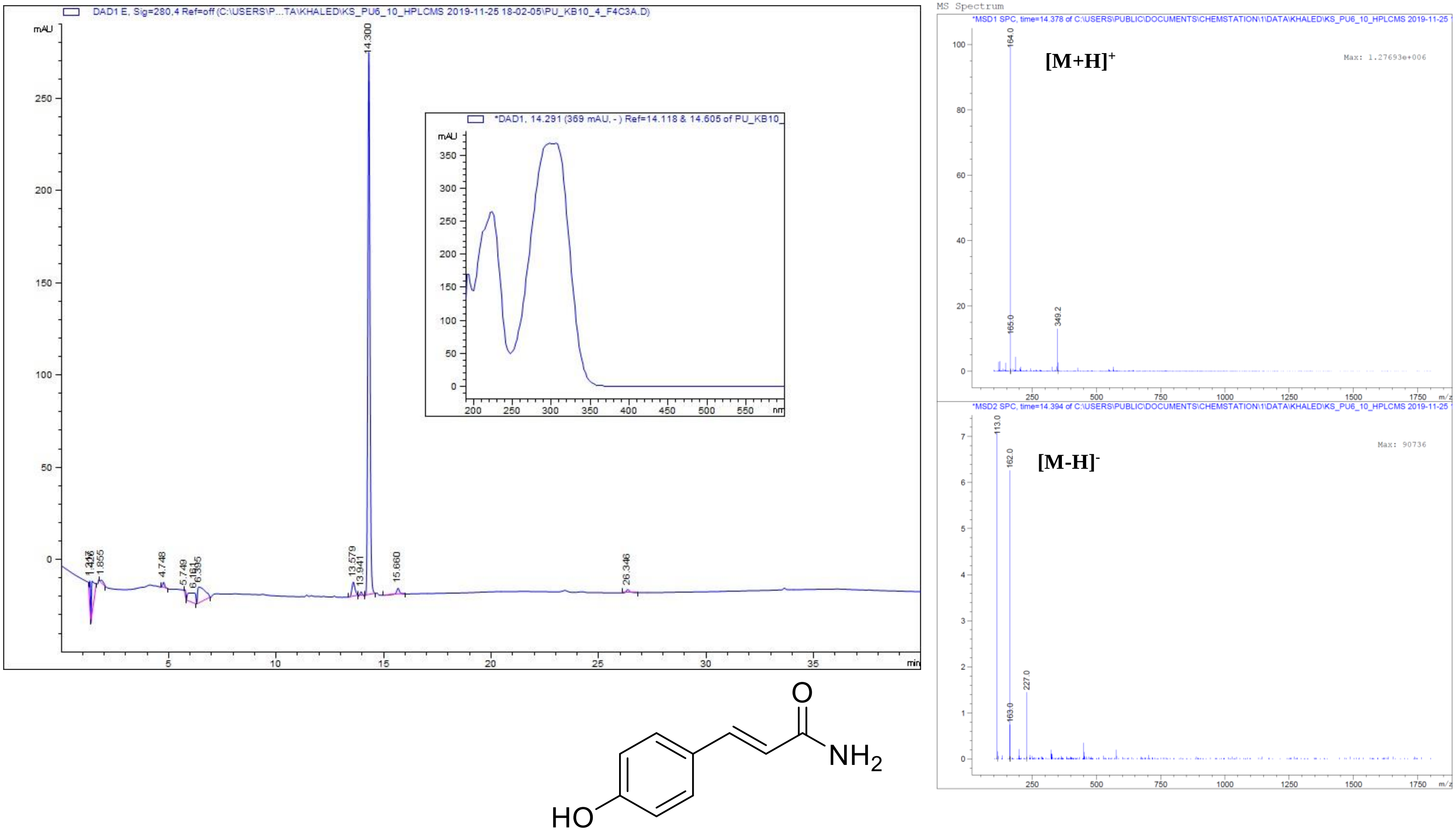

Figure S28: HPLC analysis of 4-hydroxycinnamide (**3**). HPLC-conditions: solvent A: H_2O/0.1% FA; solvent B: CH_3CN; flow rate: 0.5 mL min^{-1}; 0-30 min, 5-100% B; 30-35 min, 100% B; 35-36 min, 100-5% B; 36-40 min, 5% B; Phenomenex NX-C18 column (250 × 4.6 mm, 5 μm); 254 nm. UV-vis inset of full wavelength scan (190-600 nm). (+) and (–)-ESI-MS spectra of 4-hydroxycinnamide (**3**).
