# Supplementary material for: Bioprospecting of desert actinobacteria with special emphases on griseoviridin, mitomycin C and a new bacterial metabolite producing Streptomyces sp. PU-KB10–4
Source: BMC Microbiol. 2023 Mar 15;23:69. doi: 10.1186/s12866-023-02770-8 (PMC10015687; doi:10.1186/s12866-023-02770-8)
Supplement: Supplementary file 33 — Additional file 33: Fig. S30. 13C NMR spectrum (CD3OD, 125 MHz) of 4-hydroxycinnamide (3). [file 12866_2023_2770_MOESM33_ESM.pdf]

1D and 2D NMR spectrum of 4-hydroxycinnamide (3)

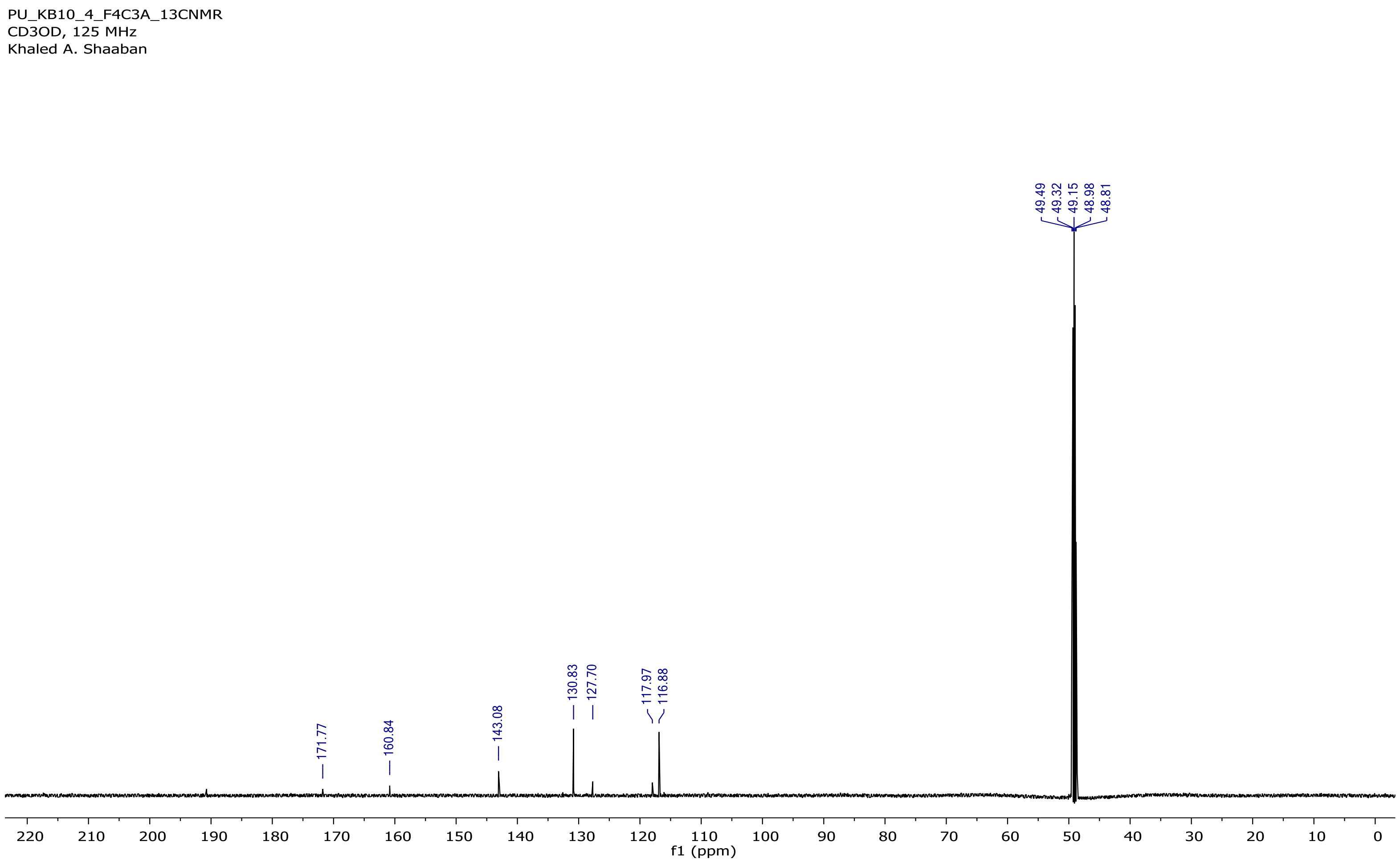

Figure S30: ^{13}C NMR spectrum (CD_3OD, 125 MHz) of 4-hydroxycinnamide (**3**).
