# Supplementary material for: Bioprospecting of desert actinobacteria with special emphases on griseoviridin, mitomycin C and a new bacterial metabolite producing Streptomyces sp. PU-KB10–4
Source: BMC Microbiol. 2023 Mar 15;23:69. doi: 10.1186/s12866-023-02770-8 (PMC10015687; doi:10.1186/s12866-023-02770-8)
Supplement: Supplementary file 34 — Additional file 34: Fig. S31. 1H (500 MHz) and 13C (125 MHz) NMR spectra of 4-hydroxycinnamide (3) in CD3OD. [file 12866_2023_2770_MOESM34_ESM.pdf]

1D and 2D NMR spectrum of 4-hydroxycinnamide (3)

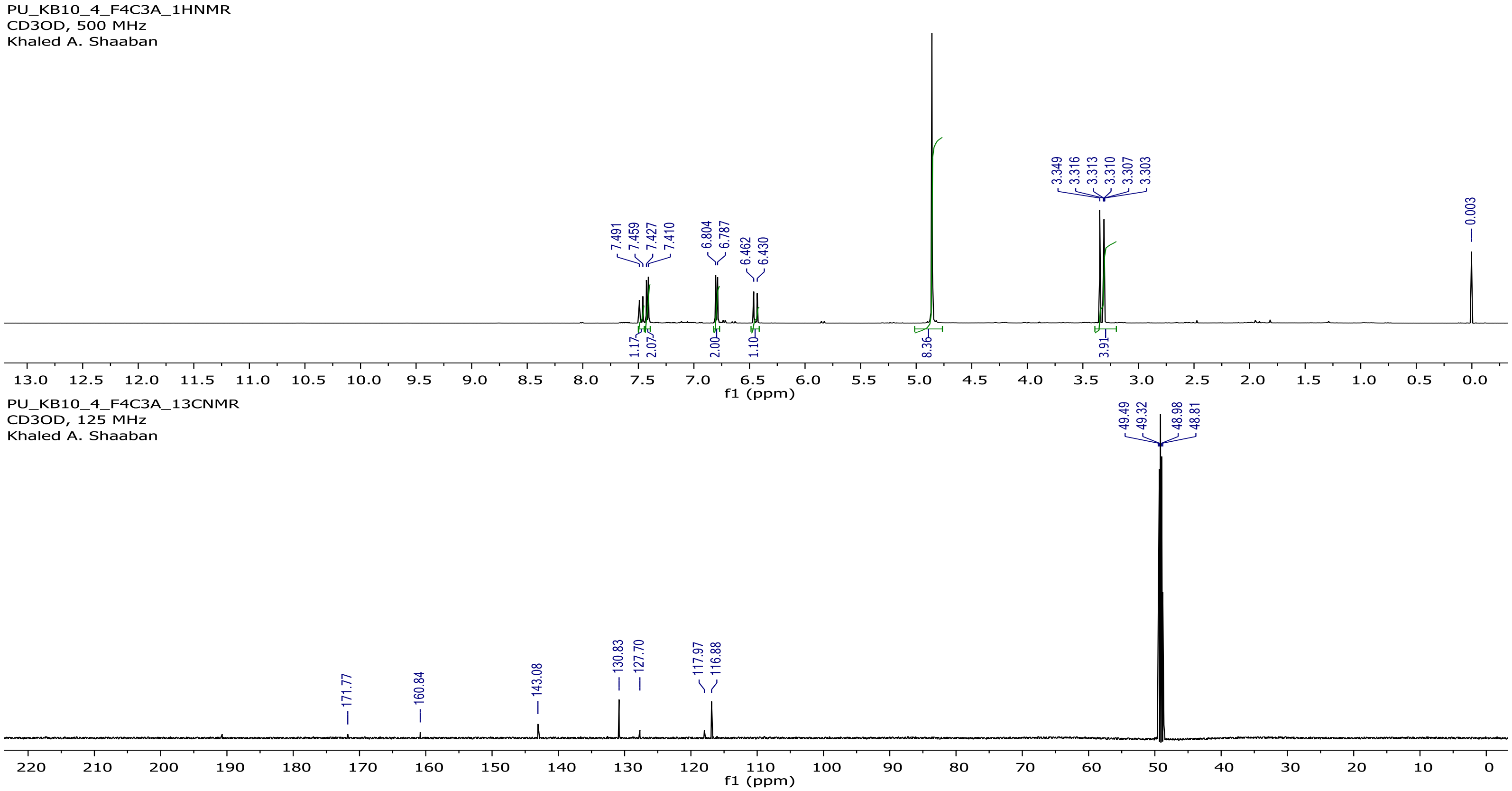

Figure S31: ^{1}H (500 MHz) and ^{13}C (125 MHz) NMR spectra of 4-hydroxycinnamide (**3**) in CD_3OD.
