# Supplementary material for: Bioprospecting of desert actinobacteria with special emphases on griseoviridin, mitomycin C and a new bacterial metabolite producing Streptomyces sp. PU-KB10–4
Source: BMC Microbiol. 2023 Mar 15;23:69. doi: 10.1186/s12866-023-02770-8 (PMC10015687; doi:10.1186/s12866-023-02770-8)
Supplement: Supplementary file 41 — Additional file 41.. Physiochemical properties of compounds 1-3. [file 12866_2023_2770_MOESM41_ESM.pdf]

**Physicochemical Properties of Compounds 1-3**

**Griseoviridin (1).** $C_{22}H_{27}N_3O_7S$ (477); white powder; *HPLC-R*$_t$ = 17.43 min (Supplementary Information, Figure S15); UV/vis $\lambda_{max}$ 220, 290 (sh) nm; $^1$H NMR ($CD_3OD$, 400 MHz) and $^{13}$C NMR ($CD_3OD$, 100 MHz), see Supplementary Information, Table S4; (–)-ESI-MS: *m/z* 476 [M – H]$^–$, 522 [M + HCOO]$^–$; (+)-ESI-MS: *m/z* 460 [(M-$H_2O$) + H]$^+$. See Supplementary Information, Figures S16-S24.

**Mitomycin C (2).** $C_{15}H_{18}N_4O_5$ (334); red solid; *HPLC-R*$_t$ = 13.50 min (Supplementary Information, Figure S25); UV/vis $\lambda_{max}$ 215, 250 (sh), 355 nm; $^1$H NMR ($CD_3OD$, 400 MHz) and $^{13}$C NMR ($CD_3OD$, 100 MHz), see Supplementary Information, Figures S26-S29; (–)-ESI-MS: *m/z* 333 [M – H]$^–$; (+)-ESI-MS: *m/z* 335 [M + H]$^+$.

**4-Hydroxycinnamide (3).** $C_9H_9NO_2$ (163); pale-yellow solid; *HPLC-R*$_t$ = 14.30 min (Supplementary Information, Figure S30); UV/vis $\lambda_{max}$ 200, 221, 250, 318 nm; $^1$H NMR ($CD_3OD$, 500 MHz) $\delta$ 7.48 (d, 1H, *J*=15.7 Hz, 1′-H), 7.42 (d, 2H, *J*=8.6 Hz, 3-H/5-H), 6.80 (d, 2H, *J*=8.5 Hz, 2-H/6-H), 6.45 (d, 1H, *J*=15.8 Hz, 2′-H); $^{13}$C NMR ($CD_3OD$, 125 MHz) $\delta$ 171.8 (C-3′), 160.8 (C-1), 143.1 (CH-1′), 130.8 (CH-3/CH-5), 127.7 (C-4), 118.0 (CH-2′), 116.9 (CH-2/CH-6); $^1$H NMR (DMSO-$d_6$, 400 MHz) $\delta$ 9.83 (brs, 1H, 1-OH), 7.38/6.93 (brs, 2H, 3′-$NH_2$), 7.38 (d, 2H, *J*=8.6 Hz, 3-H/5-H), 7.31 (d, 1H, *J*=15.8 Hz, 1′-H), 6.78 (d, 2H, *J*=8.6 Hz, 2-H/6-H), 6.38 (d, 1H, *J*=15.8 Hz, 2′-H); $^{13}$C NMR (DMSO-$d_6$, 100 MHz) $\delta$ 167.1 (C-3′), 158.8 (C-1), 139.2 (CH-1′), 129.2 (CH-3/CH-5), 125.8 (C-4), 118.7 (CH-2′), 115.7 (CH-2/CH-6); (–)-ESI-MS: *m/z* 162 [M – H]$^–$; (+)-ESI-MS: *m/z* 164 [M + H]$^+$.
