# Supplementary material for: Bioprospecting of desert actinobacteria with special emphases on griseoviridin, mitomycin C and a new bacterial metabolite producing Streptomyces sp. PU-KB10–4
Source: BMC Microbiol. 2023 Mar 15;23:69. doi: 10.1186/s12866-023-02770-8 (PMC10015687; doi:10.1186/s12866-023-02770-8)
Supplement: Supplementary file 42 — Additional file 42: Fig. S38. (A) Streptomyces sp. PU-KB10-4 grown on a M2-medium agar plate. (B) HPLC/UV analyses of the generated extracts produced by Streptomyces sp. PU-KB10-4 using three different media (A-, SG- and M2 media). SG medium was selected for scale-up as 10 L based on the high production of the major metabolite (Compound 1). [file 12866_2023_2770_MOESM42_ESM.pdf]

Media optimization of *Streptomyces* sp. PU-KB10-4

(A) ***Streptomyces*** **sp. PUKB10-4**

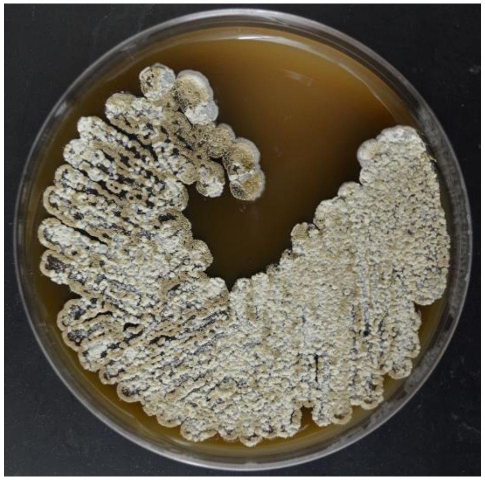

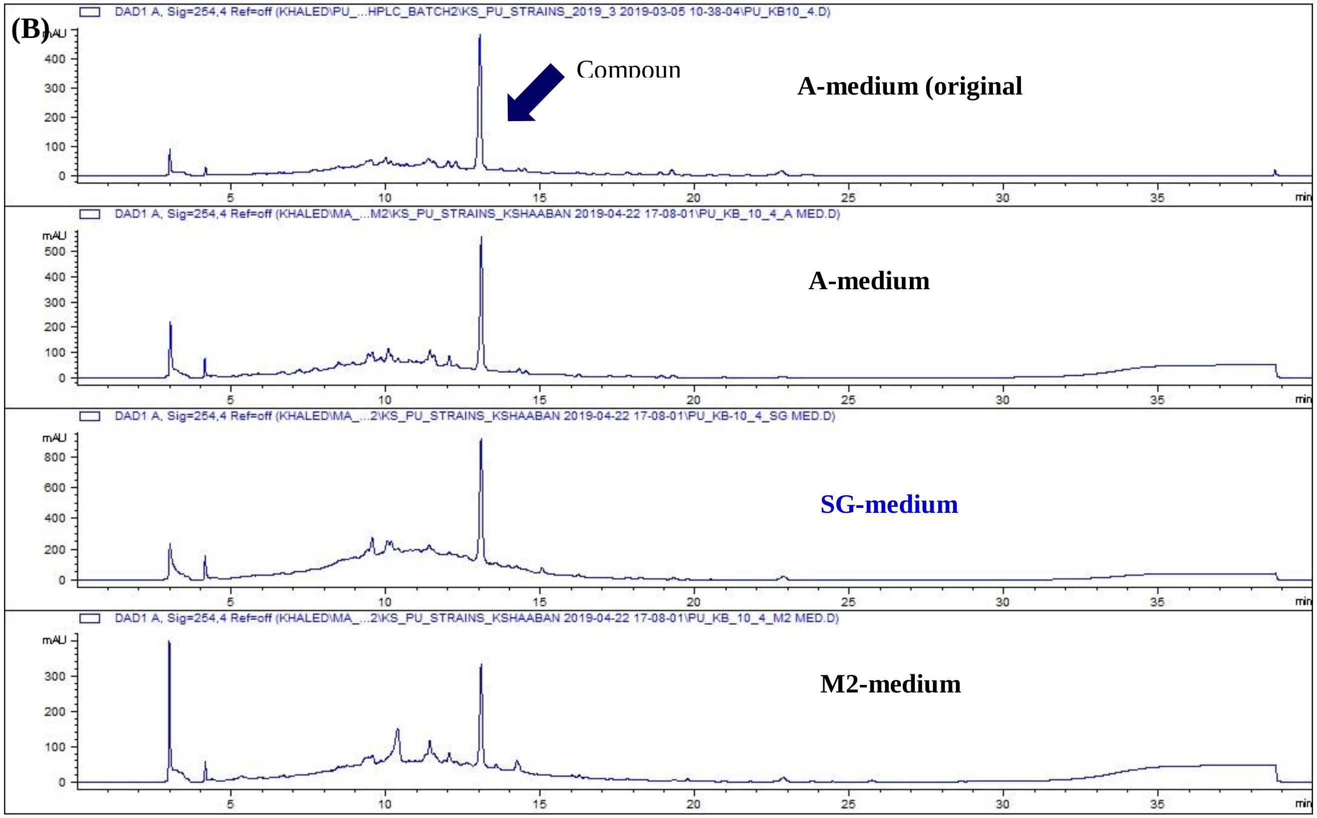

Figure S38. (A)*Streptomyces* sp. PU-KB10-4 grown on a M2-medium agar plate. **(B)** HPLC/UV analyses of the generated extracts produced by *Streptomyces* sp. PU-KB10-4 using three different media (A-, SG- and M2 media). SG medium was selected for scale-up as 10 L based on the high production of the major metabolite (Compound **1**).
