# Supplementary material for: Bioprospecting of desert actinobacteria with special emphases on griseoviridin, mitomycin C and a new bacterial metabolite producing Streptomyces sp. PU-KB10–4
Source: BMC Microbiol. 2023 Mar 15;23:69. doi: 10.1186/s12866-023-02770-8 (PMC10015687; doi:10.1186/s12866-023-02770-8)
Supplement: Supplementary file 43 — Additional file 43: Fig. S39. (A) HPLC/UV analyses of the mycelium and XAD-extracts produced by Streptomyces sp. PU-KB10-4. Compound 1 was the major compound of the strain extract (42 mg/L). (B) Antimicrobial activity of crude extracts of PU-KB10–4 grown in three different media: the crude extracts of PU-KB10–4 obtained from A-media, M2- media and SG media showed less activity against Gram-negative bacteria (Escherichia coli) and huge zones of inhibition against Gram-positive bacteria (Staphylococcus aureus). [file 12866_2023_2770_MOESM43_ESM.pdf]

(A)

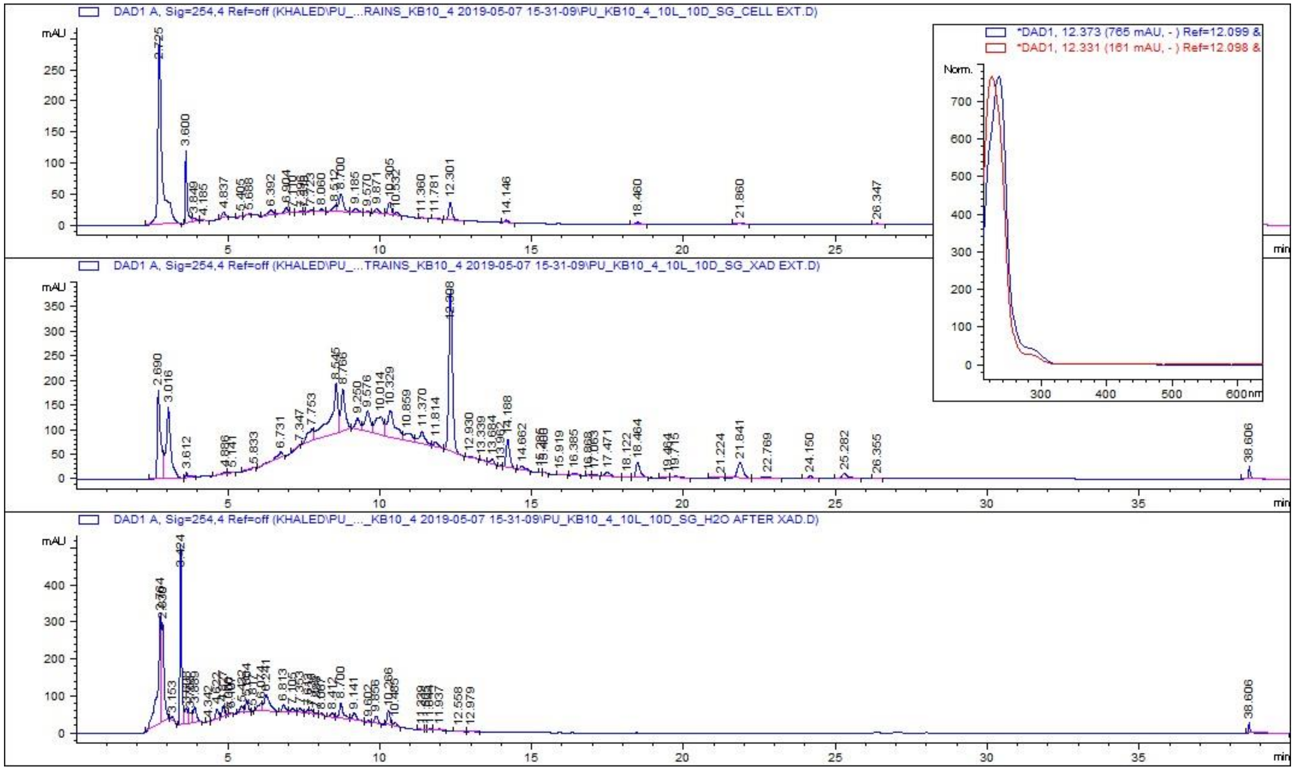

(B)

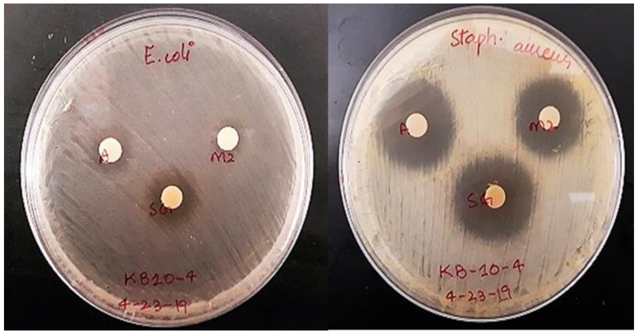

Figure S39: (A) HPLC/UV analyses of the mycelium and XAD-extracts produced by *Streptomyces* sp. PU-KB10-4. Compound **1** was the major compound of the strain extract (42 mg/L). **(B)** Antimicrobial activity of crude extracts of PU-KB10-4 grown in three different media: the crude extracts of PU-KB10-4 obtained from A-media, M2- media and SG media showed less activity against Gram-negative bacteria (*Escherichia coli*) and huge zones of inhibition against Gram-positive bacteria (*Staphylococcus aureus*).
