# Supplementary material for: Bioprospecting of desert actinobacteria with special emphases on griseoviridin, mitomycin C and a new bacterial metabolite producing Streptomyces sp. PU-KB10–4
Source: BMC Microbiol. 2023 Mar 15;23:69. doi: 10.1186/s12866-023-02770-8 (PMC10015687; doi:10.1186/s12866-023-02770-8)
Supplement: Supplementary file 44 — Additional file 44: Fig. S40. (A and B) Cytotoxicity and antimicrobial analysis of PU-KB10-4 fractions obtained after RP-18 column chromatography (C and D) Cytotoxicity and antimicrobial analysis of PU-KB10-4 after combining the initial column fractions. [file 12866_2023_2770_MOESM44_ESM.pdf]

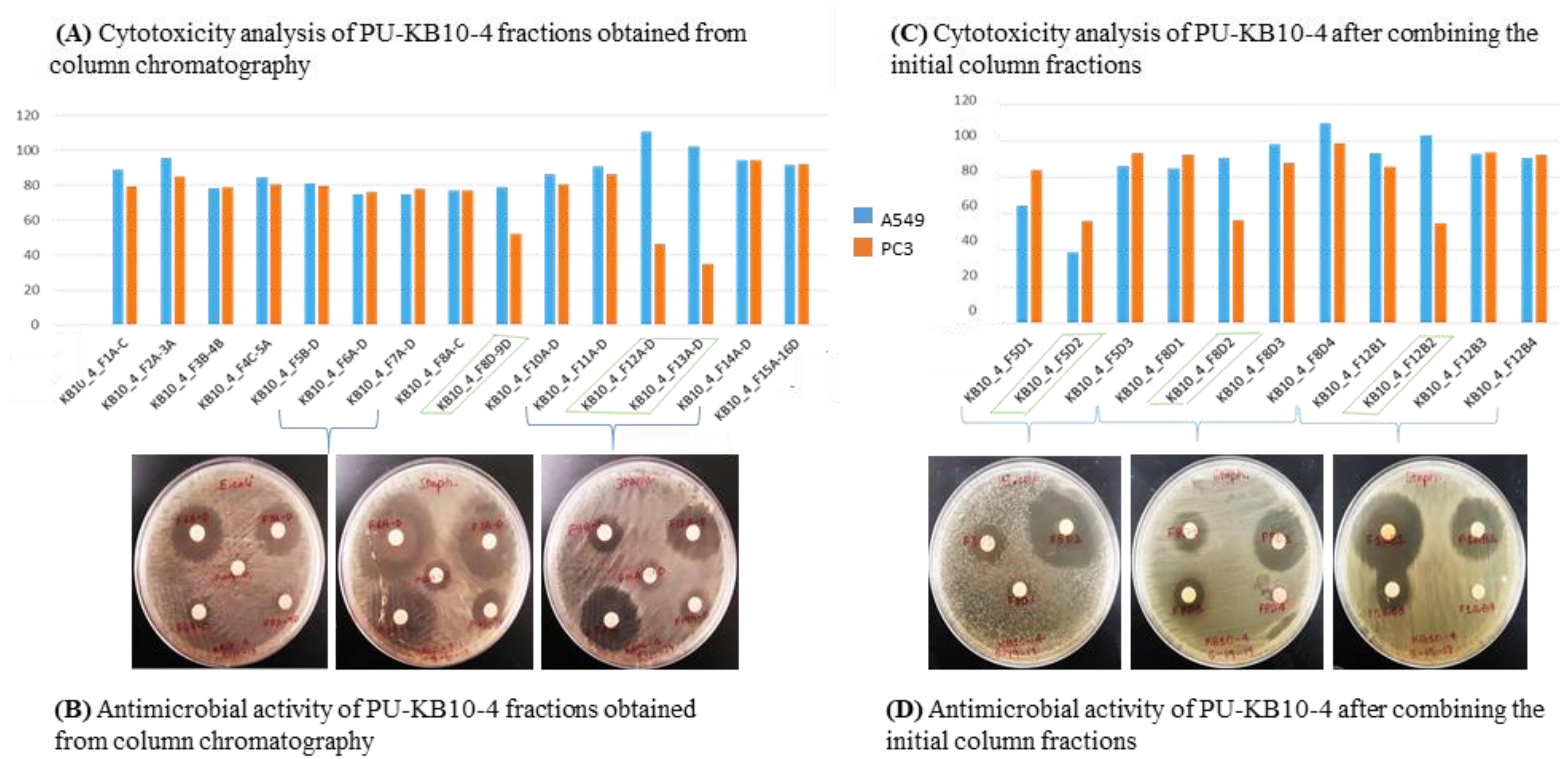

Figure S40: (A and B) Cytotoxicity and antimicrobial analysis of PU-KB10-4 fractions obtained after RP-18 column chromatography **(C and D)** Cytotoxicity and antimicrobial analysis of PU-KB10-4 after combining the initial column fractions
